# Clinical efficacy of therapeutic footwear with a rigid rocker sole in the prevention of recurrence in patients with diabetes mellitus and diabetic polineuropathy: A randomized clinical trial

**DOI:** 10.1371/journal.pone.0219537

**Published:** 2019-07-11

**Authors:** Mateo López-Moral, José Luis Lázaro-Martínez, Esther García-Morales, Yolanda García-Álvarez, Francisco Javier Álvaro-Afonso, Raúl J. Molines-Barroso

**Affiliations:** Diabetic Foot Unit, Facultad de Medicina, Universidad Complutense de Madrid, Instituto de Investigación Sanitaria del Hospital Clínico San Carlos (IdISSC), Madrid, Spain; IRCCS E. Medea, ITALY

## Abstract

**Background:**

Therapeutic footwear becomes the first treatment line in the prevention of diabetic foot ulcer and future complications of diabetes. Previous studies and the International Working Group on the Diabetic Foot have described therapeutic footwear as a protective factor to reduce the risk of re-ulceration. In this study, we aimed to analyze the efficacy of a rigid rocker sole to reduce the recurrence rate of plantar ulcers in patients with diabetic foot.

**Methods:**

Between June 2016 and December 2017, we conducted a randomized controlled trial in a specialized diabetic foot unit.

**Participants and intervention:**

Fifty-one patients with diabetic neuropathy who had a recently healed plantar ulcer were randomized consecutively into the following two groups: therapeutic footwear with semi-rigid sole (control) or therapeutic footwear with a rigid rocker sole (experimental). All patients included in the study were followed up for 6 months (one visit each 30 ± 2 days) or until the development of a recurrence event.

**Main outcome and measure:**

Primary outcome measure was recurrence of ulcers in the plantar aspect of the foot.

**Findings:**

A total of 51 patients were randomized to the control and experimental groups. The median follow-up time was 26 [IQR—4.4—26.1] weeks for both groups. On an intention-to-treat basis, 16 (64%) and 6 (23%) patients in the control and experimental groups had ulcer recurrence, respectively. Among the group with >60% adherence to therapeutic footwear, multivariate analysis showed that the rigid rocker sole improved ulcer recurrence-free survival time in diabetes patients with polyneuropathy and DFU history (P = 0.019; 95% confidence interval, 0.086–0.807; hazard ratio, 0.263).

**Conclusions:**

We recommend the use of therapeutic footwear with a rigid rocker sole in patients with diabetes with polyneuropathy and history of diabetic foot ulcer to reduce the risk of plantar ulcer recurrence.

**Trial registration:**

ClinicalTrials.gov NCT02995863.

## Introduction

Diabetic foot ulcer (DFU) is one of the most common complications of diabetes mellitus. The lifetime incidence of DFU in patients with diabetes is approximately 19% to 34% [1]. Moreover, approximately 20% of DFU with moderate or severe infection lead different levels of amputation [2]. Patients with a history of DFU have a 2.5 times higher risk of death than those without history of DFU [3]. The mortality rate increases to 70% at 5 years after undergoing an amputation [4]. The costs of the treatment for diabetic foot complication are more expensive than those for more common cancer complications [5], amounting to $176 billion annually in the United States [6].

DFUs are caused by repetitive stress in patients with peripheral neuropathy, foot deformities, and peripheral arterial disease [1]. The DFU recurrence rate is estimated at 40% after the first year of ulcer healing and 60% within 3 years, and it increases to 65% after 5 years [1].

Almost 50% of DFUs appear on the plantar surface of the foot due to the deformity and the high levels of plantar pressure in the metatarsal heads [7].

Previous studies have reported that use of therapeutic footwear is a protective factor in reducing the risk of reulceration [8–10]. Additionally, use of rocker soles has been reported to show good results in reducing plantar pressure, preventing the first DFU, and decreasing DFU recurrence [11–14]. Several studies [10,11] have demonstrated that using semi-rigid soles is better than using standard therapeutic footwear in terms of avoiding future complications.

Uccioli et al. [11] compared the performance of semi-rigid rocker soles with that of standard footwear for reducing plantar ulcers and demonstrated that semi-rigid rocker soles reduced ulcer recurrence on the metatarsal heads. A specific density rocker outsole might be more effective in decreasing plantar pressure and consequently in reducing plantar recurrence. In fact, this result has been observed in other orthopedic treatment as different compositions of plantar orthosis has been demonstrated as a factor to reduce plantar pressure [15]. However, the different material densities in therapeutic footwear have not been studied. To the best of our knowledge, no randomized clinical trial (RCT) has clinically compared the recurrence or reulceration rate of the diabetic foot according to material density of the therapeutic footwear. Therefore, the principal aim of this study was to analyze in an intention-to-treat analysis the clinical efficacy of a rigid rocker sole in the reduction of the recurrence rate of plantar ulcers in patients with diabetic foot.

## Research design and methods

### Study design

We performed a randomized and controlled parallel (1:1) clinical trial of patients with diabetes between June 2016 and December 2017. This study was approved by a local ethics committee (Hospital Clínico San Carlos, Madrid, Spain) on October 2016 (approval no.: 16/408–P). Each patient provided written informed consent before inclusion.

### Participants

We enrolled patients from an outpatient specialized diabetic foot unit. All patients included in the study were followed for 6 months (one visit each 30 ± 2 days) or until the development of a recurrence event. Mechanical properties of insoles and therapeutic footwear are thought to be impaired after 6 months of usage [16].

In every visit, the principal investigator performed debridement of high-risk points in the forefoot areas, such as the callus and hiperquerathosis; in addition, the insole and the therapeutic footwear were examined. Adherence to treatment and activity questionnaires were given to the patients at this point of the study. When a recurrent ulcer appeared before scheduled follow-up visit, the patients were instructed to return to the diabetic foot unit.

Inclusion criteria were confirmed type 1 or type 2 diabetes, age > 18 years, loss of protective foot sensation as a result of peripheral neuropathy, and previous foot ulcer under the metatarsal head. Exclusion criteria were ulcer during examination, transmetatarsal or major amputation (below or above the knee), history of rheumatoid disease, other causes of neuropathy, critical limb ischemia as defined by the TASC II guideline [17], Charcot foot, need for a custom-made footwear due to severe foot deformity, and need for walking aids.

Patients who underwent offloading surgery were also excluded from the study, with offloading surgery defined as a “procedure performed to reduce risk of ulceration or re-ulceration in person with loss of protective sensation” [18]. Patients who rejected the therapeutic shoe for esthetic purposes were also excluded, based on a study reporting that patient dissatisfaction with the prescribed footwear results in low usage [19].

The present study is registered in ClinicalTrial.gov (Registration no.: NCT02995863).

### Therapeutic footwear

**Fig 1:**
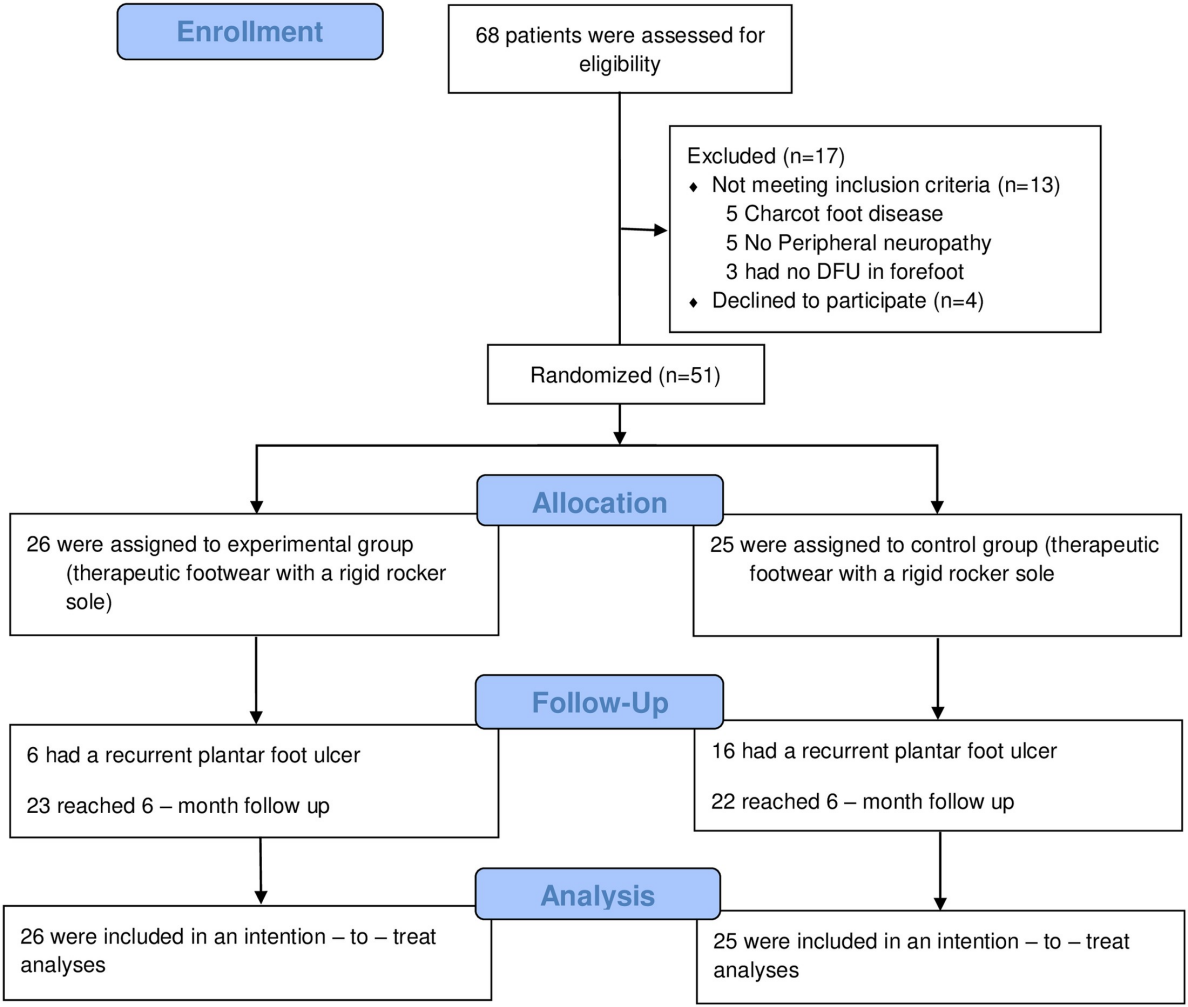
Study flow diagram.

The patients were randomly assigned to the control or experimental group. A study flow diagram is shown in (Fig 1).

Both groups wore a therapeutic footwear (Podartis s.r.l Unipersonale—Crocceta del Montello (TV), Italy) with the same general characteristics: high toe box; enough width to accommodate toe deformities such as claw or hammer toes, wide heel, and laces or buckles for fasteners. Furthermore, the shoes were free of seams, folds, and hollows. The depth of the shoe was 14 or 16 mm greater than the standard footwear to avoid friction in the vicinity of the heel counter and particularly to help the inclusion of an insole.

**Fig 2:**
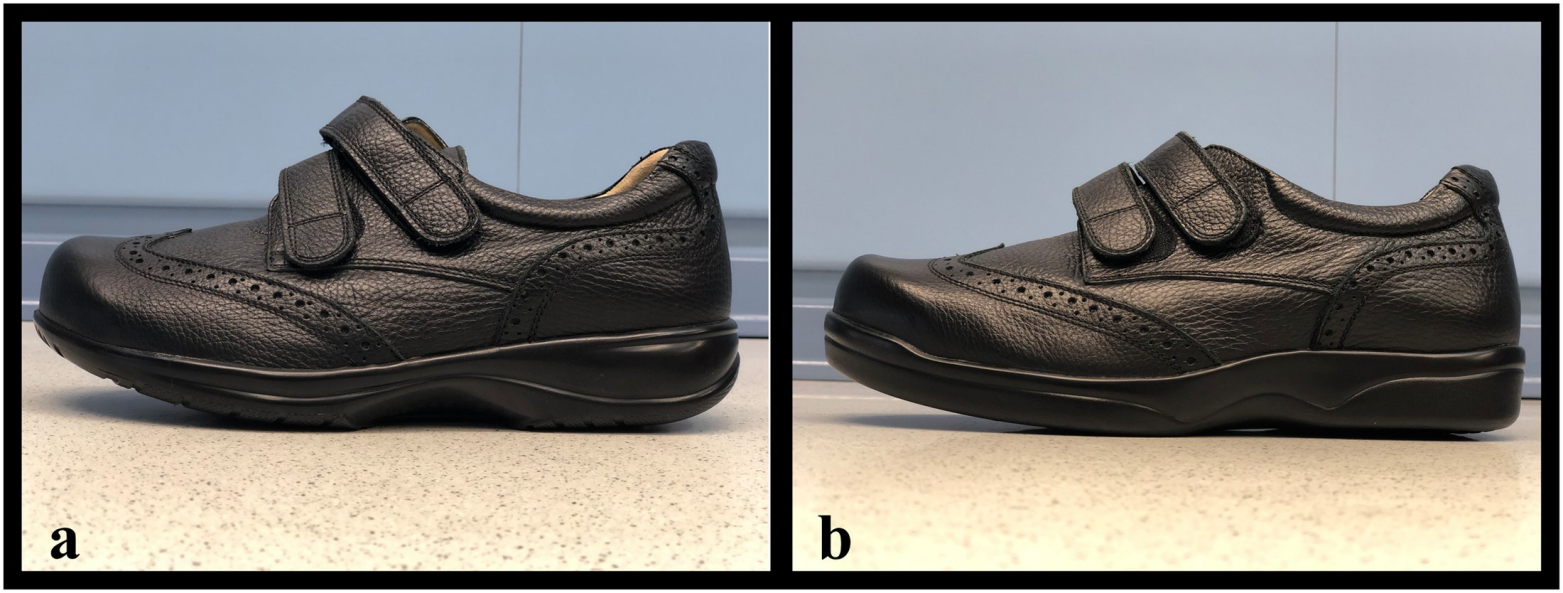
Sample of rigid and semi rigid sole footwear. (A) Rigid shoe (B). Semi-rigid shoe.

Samples of footwear with rigid and semi-rigid soles are shown in Fig 2.

In addition, all patients had a multilayer orthosis (40 shore degrees base of ethyl vinyl acetate, the dorsal cover was made of poron) with a cut-out in the affected metatarsal head. The material of the shoe was thin and made of a soft skin, while the heel counter was rigid, maintaining the heel in a proper position. All shoes had a rocker sole. The rocker sole was previously described as the anteroposterior rocker, and the starting point of the propulsion is behind the metatarsal heads to reduce plantar pressures on the forefoot [20]. The rocker angle was defined as the 20° angle between the floor and sole under the metatarsal heads.

**Fig 3:**
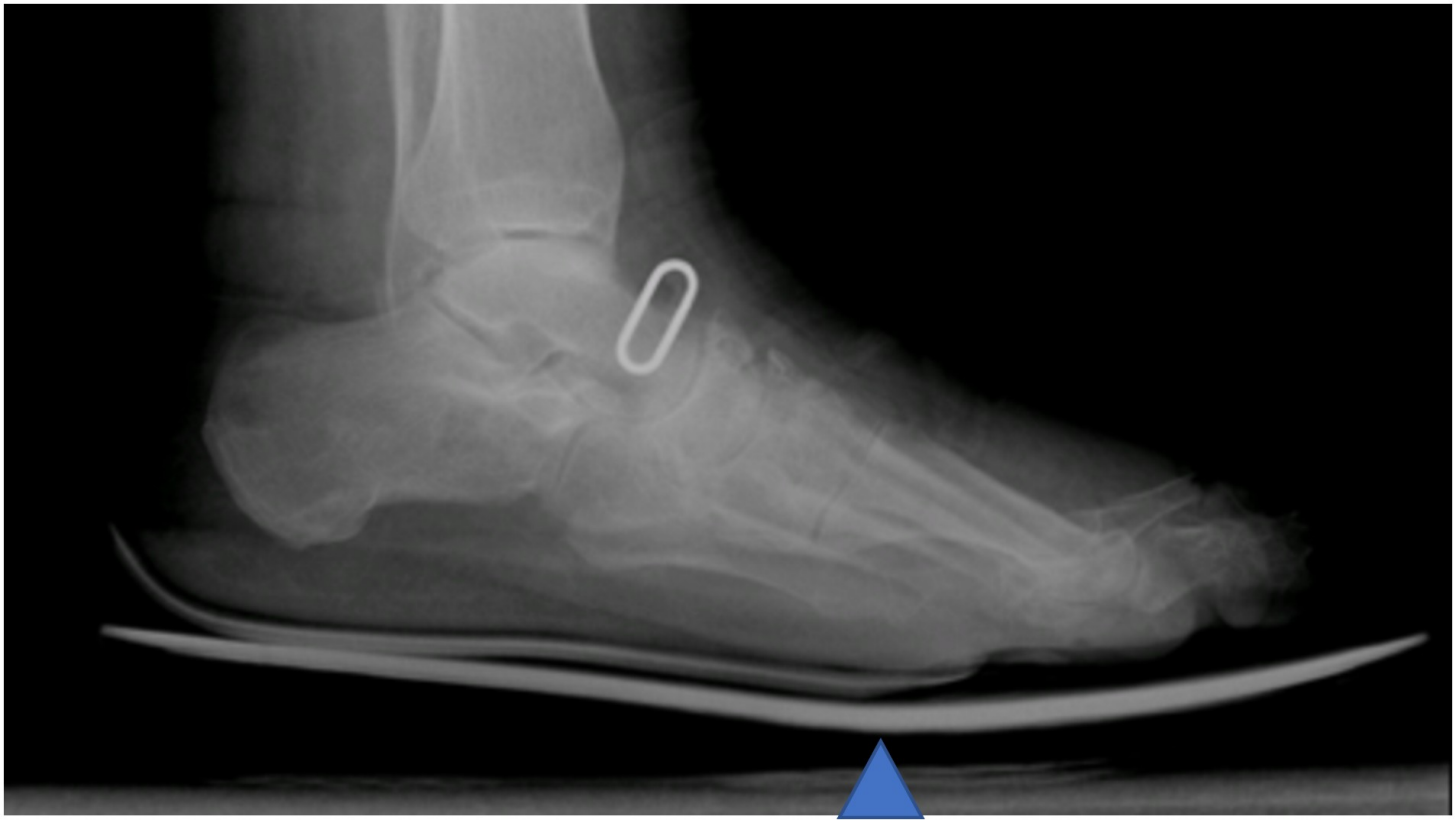
Weight-bearing lateral X-ray of the shod feet.

The principal investigator ensured the concordance between the footwear size and feet length and the proper position of the rocker behind the metatarsal heads using a weight-bearing lateral X-ray of the shod feet for all patients in both study groups (Fig 3).

Meanwhile, the sole density was different between the two groups, with participants of the control group wearing a semi-rigid rocker sole (Wellwalk technology with Vibram strips), whereas those in the experimental group wore a rigid (composite fiber) rocker sole.

### Outcome measures

The primary outcome measure was ulcer recurrence defined according to the guidelines of the International Working Group and Infectious Diseases Society of America as full-thickness wound involving the foot or ankle [21]. Two different clinicians that were blinded to the randomization diagnosed plantar ulceration.

Loss of protective sensation was confirmed by the inability to sense the pressure of a 10-g Semmes–Weinstein monofilament at three plantar foot sites and/or a vibration perception threshold >25 V as assessed via the biothesiometer (Me.Te.Da. s.r.l., Via Silvio Pellico, 4, 63074 San Benedetto del Tronto AP, Italy) [22]. Peripheral arterial disease (PAD) was defined as absence of both distal pulses and/or an ankle brachial index (ABI) of <0.9 [23]. In patients whose ABI was >1.4 or those with uncertain diagnostic findings, a systolic toe pressure of <55 mmHg, systolic ankle pressure <70 mmHg, or a toe brachial index <0.7 was considered to indicate PAD [23].

Forefoot deformities were considered when the foot presents some of the following conditions: hallux valgus, Taylor’s bunion, metatarsal head bone prominences, or toe contractures such as hammer-toe, claw-toe, or mallet-toe deformities [24–26].

The following range of joint mobility was measured using a two-armed goniometer: ankle joint, the first metatarsophalangeal joint (MPJ), and the subtalar joint.

Ankle dorsiflexion was examined with the patient on supine position, by placing the subtalar joint in neutral position, and forcefully dorsiflexing at the ankle joint, measuring the angle formed between the bisections of the fibula and lateral foot, which were marked previously on the patient’s skin [24,25,27,28].

Dorsiflexion of the first MPJ was recorded with the seated patient in resting position (first MPJ ROM) and with the standing patient in weight-bearing position (first MPJ ROMw-b) [24,29].

Finally, subtalar joint measurement ranges were examined with the patient in the prone position, holding the calcaneus with one hand and the talar head/neck with the thumb and index finger of the other hand. Adduction (inversion ROM) and abduction ranges of movements (eversion ROM) with the hand on the calcaneus were applied [24]. The analysis of the foot position was stratified into neutral, pronated, or supinated according to the Foot Posture Index (FPI) described previously [30]. Foot type, presence of deformities and joint mobility were recorded for the same clinician (MLM). In addition, a physical activity questionnaire (IPAQ) [31] was used to assess the activity level of each enrolled patient in all seven physician consultations.

Therapeutic footwear adherence was evaluated using a questionnaire in each consultation [32], and patients who achieved more than 60% of adherence to therapeutic footwear were included in a statistical sub-analysis [33]. At footwear delivery, the study investigator randomly assigned subjects using the website Randomization.com (http://www.randomization.com) in a balanced design (1:1) to (1) therapeutic footwear with a semi-rigid sole or to (2) therapeutic footwear with a rigid sole.

### Statistical analyses

In an intention-to-treat analyses, a univariate analysis for risk factors associated with ulcer location were performed using Chi-square test for categorical variables and the Student t-test for quantitative variables using SPSS version 20.0 (SPSS, Chicago, IL, USA).

The strength of difference in the effect size was calculated by Phi coefficient for chi-square test and r coefficient for non-parametric test considering the values >0.01 as a small effect, >0.30 as a medium effect, and >0.50 as a large effect. Cohen’s d was calculated as the effect size for parametric test using the effect size calculator (http://www.uccs.edu/~lbecker/) and considering the values >0.2, >0.5, and >0.8 as small, moderate, and large effects, respectively [34].

Relative risk reduction ($\text{RRR}=\frac{\text{Experimental}\;\text{group}\;\text{risk}-\text{Control}\;\text{group}\;\text{risk}}{\text{Control}\;\text{group}\;\text{risk}}$) and number needed to treat ($\text{NNT}=\frac{1}{\text{Experimental}\;\text{group}\;\text{risk}-\text{Control}\;\text{group}\;\text{risk}}$) were used to estimate the size of the effect.

Differences in survival between the subgroup (>60% adherence to treatment) were evaluated using the log-rank test and were expressed using Kaplan-Meier curves. Continuous and categorical variables with P<0.10 were selected as covariates in the univariate analysis to develop a Cox proportional hazards survival model for determining ulcer recurrence-free survival time; these variables were expressed using hazard ratios with a forward stepwise selection method. P values <0.05 were considered statistically significant, with confidence intervals of 95%.

A 3-year follow-up study of patients with diabetic foot ulcer and high degree of comorbidities [35] showed a recurrence rate of 48.2%. As a relevant risk reduction, we assumed a difference in the recurrence rate of 20% in the rigid outsole group on the basis of what we considered a relevant risk reduction compared with the semi-rigid outsole footwear. With 0.05 setting (one-sided), power of 0.80 in a ×2 analysis, and an anticipated loss to follow-up of 20%, we intended to include 138 patients. Because of the low recruitment rate, the actual sample size was 51, which, on an intention-to-treat basis, yielded powers of 0.68 (one-sided) and 0.54 (two-sided) [10].

## Results

A total of 51 patients were included in the study, and they were randomly assigned to two different groups: the control group (n = 25 patients) and experimental group (n = 26 patients). Data on demographics, diabetes, and foot complications were collected at baseline (Table 1).

**Table 1:** Patients baseline characteristics (N = 51).

(N = 51 patients)	Control group n = 25	Experimental group n = 26
Male n (%)	23 (92)	24 (92)
Female n (%)	2 (8)	2 (8)
Type 1 DM n (%)	1 (4)	0 (0)
Type 2 DM n (%)	24 (96)	26 (100)
Retinopathy n (%)	8 (38)	13 (50)
Nephropathy n (%)	3 (12)	2 (8)
Foot deformity n (%)	18 (72)	22 (85)
Previous Amputation n (%)	9 (36)	13 (50)
Mean age ± SD (years)	60 ± 8.6	61 ± 8.1
Body mass index (kg/cm^2^), mean ± SD	30.07 ± 4.24	28.71 ± 4.97
Ankle mobility joint (degrees), mean ± SD	88.12 ± 4.52	89.54 ± 5.58
Glycated hemoglobin mmol/mol (%), mean ± SD	7.50 ± 1.97	7.52 ± 1.17
PAD n (%)	8 (32)	5 (19)
Foot Posture Index	2.76 ± 5.29	1.66 ± 4.86
Diabetes mellitus (years), mean ± SD	17 ± 10.0	14 ± 8.4
Hallux mobility joint (degrees), mean ± SD	29.44 ± 19.00	28.66 ± 18.58

DM, diabetes mellitus; PAD, Peripheral arterial disease; SD, standard deviation. Control group: the semi-rigid rocker sole group; experimental group: the rigid rocker sole footwear group.

In the intention-to-treat univariate analysis, statistical significance was only observed with the treatment groups (control/experimental allocation). Other variables were not associated with the risk of recurrent events (Table 2).

**Table 2:** Univariate analyses.

(N = 51 patients)	Non-ulcerated group n = 29	Ulcerated group n = 22	P-value	Effect size
Male n (%)	28 (97)	19 (86)	0.180	0.188^a^
Female n (%)	1 (3)	3 (14)		
Type 1 DM n (%)	1 (3)	0 (0)	0.379	0.123 ^a^
Type 2 DM n (%)	28 (97)	22 (100)		
Retinopathy n (%)	12 (41)	9 (41)	0.973	-0.005 ^a^
Nephropathy n (%)	3 (10)	2 (9)	0.881	-0.021 ^a^
Foot deformity n (%)	23 (79)	17 (77)	0.861	-0.025 ^a^
Previous Amputation n (%)	15 (52)	7 (32)	0.155	-0.199 ^a^
Mean age ± SD (years)	61 ± 8.1	60 ± 8.7	0.775	-0.004 ^b^
Body mass index (kg/cm^2^), mean ± SD	29.59 ± 4.56	29.10 ± 4.83	0.717	0.052 ^b^
Ankle mobility joint (degrees), mean ± SD	89.55 ± 4.50	87.91 ± 5.75	0.275	0.156 ^b^
Glycated hemoglobin mmol/mol (%), mean ± SD	7.39 ± 1.18	7.67 ± 2.04	0.579	-0.083 ^b^
PAD n (%)	6 (21)	7 (32)	0.366	0.126 ^a^
Foot Posture Index	2.48 ± 5.04	1.82 ± 5.16	0.648	0.064 ^b^
Diabetes mellitus (years), mean ± SD	15 ± 10.0	17 ± 8.3	0.563	-0.108 ^b^
Hallux mobility joint (degrees), mean ± SD	27.55 ± 19.24	31.00 ± 17.98	0.514	-0.092 ^b^
Control group n (%)	9 (31)	16 (73)	0.003*	-0.413 ^a^
Experimental group n (%)	20 (69)	6 (27)		

DM, diabetes mellitus; PAD, peripheral arterial disease; SD, standard deviation.

^a^ For categorical variables: chi-square test, as the Phi coefficient: 0.01 represents a small effect, 0.30 represents a medium effect, and 0.50 represents a large effect.

^b^ For normally distributed variables: Student’s t-test was used for independent samples; effect size was given by Cohen’s d: >0.2 for a small effect, >0.5 for a moderate effect, and >0.8 for a large effect; d is positive if the mean difference is in the predicted direction.

*P <0.05 indicates significant association.

The results of the IPAQ questionnaire showed that all patients had a low degree of physical activity (<600 min of walking per week).

All patients included in the study were followed for 6 months or until they had a recurrence event; in addition, all patients attended all scheduled follow-up visits. Twenty-nine patients (56.8%) completed the 6 months of follow-up, and they did not have any recurrence. Twenty-two patients (43.2%) had a recurrent foot ulcer during a 6-month follow-up period (Table 2). Therefore, the median follow-up time of the sample was 26 [IQR—4.4—26.1] weeks.

A total of six patients in the experimental group (23%) and 16 (64%) in the control groups had recurrence. Six patients (37.5%) in the control group had recurrence in the first metatarsal head, 2 (12.5%) in the second metatarsal head, 1 (6.25%) in the third metatarsal head, and 4 (25%) in the fourth metatarsal head, and 2 (12.5%) patient had a recurrence in the interphalangeal joint of the hallux and 1 (6.25%) in the fifth metatarsal head. For the experimental group, 1 (16.6%) patient had a recurrence in the second metatarsal head, 2 (33.3%) in the third metatarsal head, 1 (16.6%) in the fourth metatarsal head, and 2 (33.3%) patients had a recurrence in the fifth metatarsal head.

The RRR for recurrence via the use of rigid rocker sole compared with the semi-rigid rocker sole was 64%, and the NNT was 2.43.

Table 3 shows the univariate analysis in the subgroup of patients who showed more than 60% adherence to therapeutic footwear.

**Table 3:** Univariate analysis of patients showing high adherence to therapeutic footwear.

(N = 46 patients)	Non-ulcerated group n = 28	Ulcerated group n = 18	P-value	Effect size
Male n (%)	27 (96)	16 (89)	0.312	0.149 ^a^
Female n (%)	1 (4)	2 (11)		
Type 1 DM n (%)	1 (4)	0 (0)	0.418	0.120 ^a^
Type 2 DM n (%)	27 (96)	18 (100)		
Retinopathy n (%)	11 (39)	6 (33)	0.683	-0.060 ^a^
Nephropathy n (%)	3 (11)	2 (11)	0.966	0.006 ^a^
Foot deformity n (%)	22 (79)	13 (72)	0.622	-0.073 ^a^
Previous Amputation n (%)	14 (50)	3 (17)	0.022*	-0.337 ^a^
Mean age ± SD (years)	61 ± 8.2	59 ± 9.3	0.677	-0.064 ^b^
Body mass index (kg/cm^2^), mean ± SD	29.68 ± 4.61	29.77 ± 4.97	0.948	-0.009 ^b^
Ankle mobility joint (degrees), mean ± SD	89.43 ± 4.53	88.28 ± 6.23	0.504	0.104 ^b^
Glycated hemoglobin mmol/mol (%), mean ± SD	7.45 ± 1.17	7.69 ± 2.25	0.680	-0.066 ^b^
PAD n (%)	6 (21)	7 (39)	0.199	0.189
Foot Posture Index	2.36 ± 5.09	1.72 ± 5.50	0.696	0.060 ^b^
Diabetes mellitus (years), mean ± SD	15 ± 10.0	17 ± 9.2	0.505	-0.103 ^b^
Hallux mobility joint (degrees), mean ± SD	28.54 ± 18.84	33.89 ± 17.74	0.336	-0.144 ^b^
Control group n (%)	9 (32)	14 (78)	0.003*	-0.445 ^a^
Experimental group n (%)	19 (68)	4 (22)		

DM, diabetes mellitus; PAD, peripheral arterial disease; SD, standard deviation

^a^ For categorical variables: Chi-square test, as the Phi coefficient: where an effect size of 0.01 representing small effect, 0.30 medium effect, and 0.50 large effect.

^b^ For normally distributed variables: for independent sample, Student’s t-test; effect size as the Cohen’s d: representing effect size values >0.2 as small effect, >0.5 as moderate effect, and >0.8 as large effect; d is positive if the mean difference is in the predicted direction.

*P <0.05 indicates significant association.

High adherence was a priori defined as >60% of steps in therapeutic footwear; low adherence was a priori defined as <60% of steps in therapeutic footwear.

**Fig 4:**
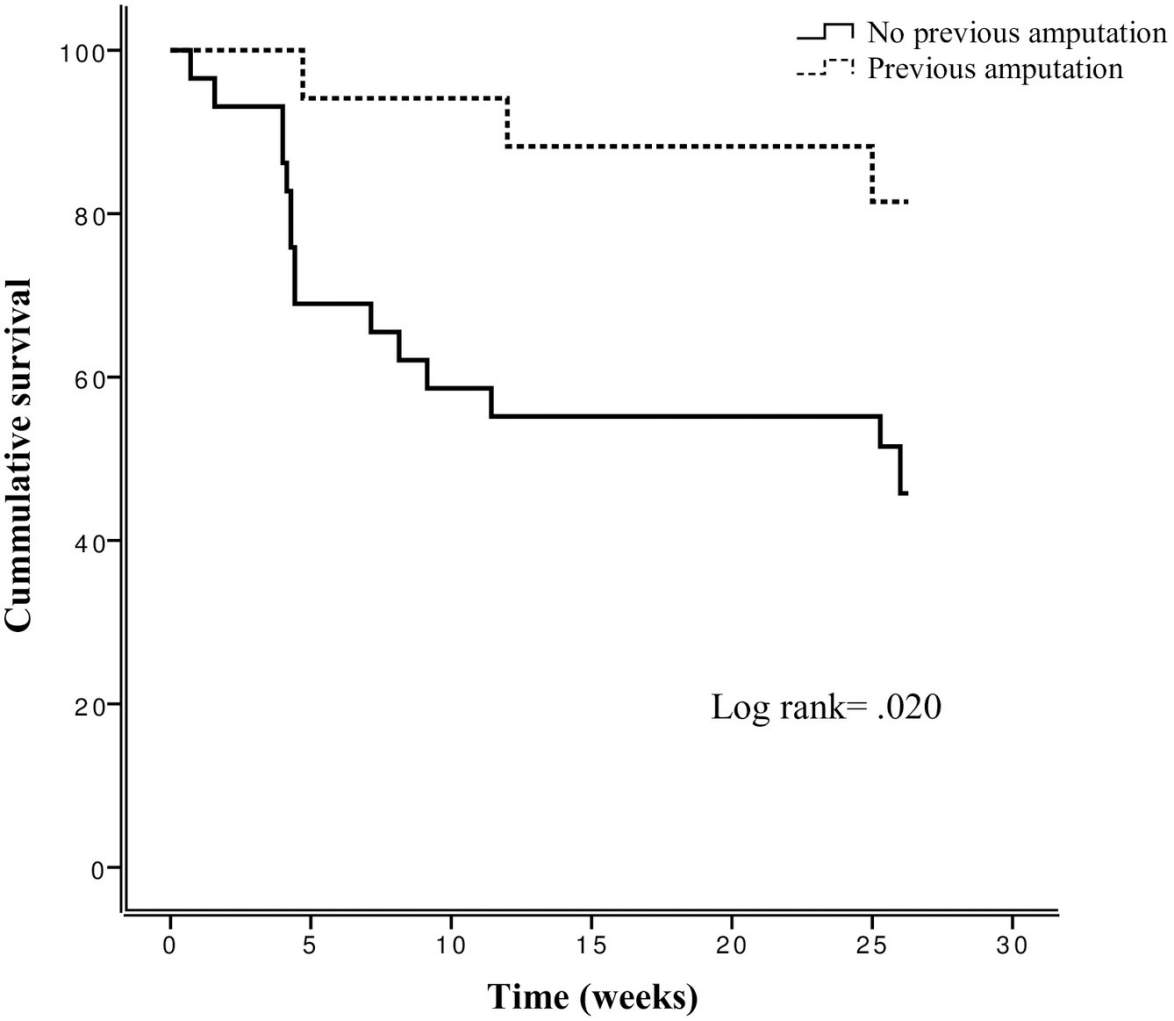
Kaplan-Meier plots for previous amputation on cumulative of survival of foot ulcer recurrence over 6 months of follow-up in patients with high adherence to therapeutic footwear (N = 46).

Fig 4 shows the association between previous amputation and recurrence-free survival time (P = 0.020) in patients with high adherence to therapeutic footwear (N = 46).

Ulcer survival curves were also significantly different between the study groups (P = 0.005). The control and experimental groups showed recurrence-free survival median times of 25 [IQR—4.7—26.0] weeks and 26 [IQR—25.8—26.1] weeks, respectively.

**Fig 5:**
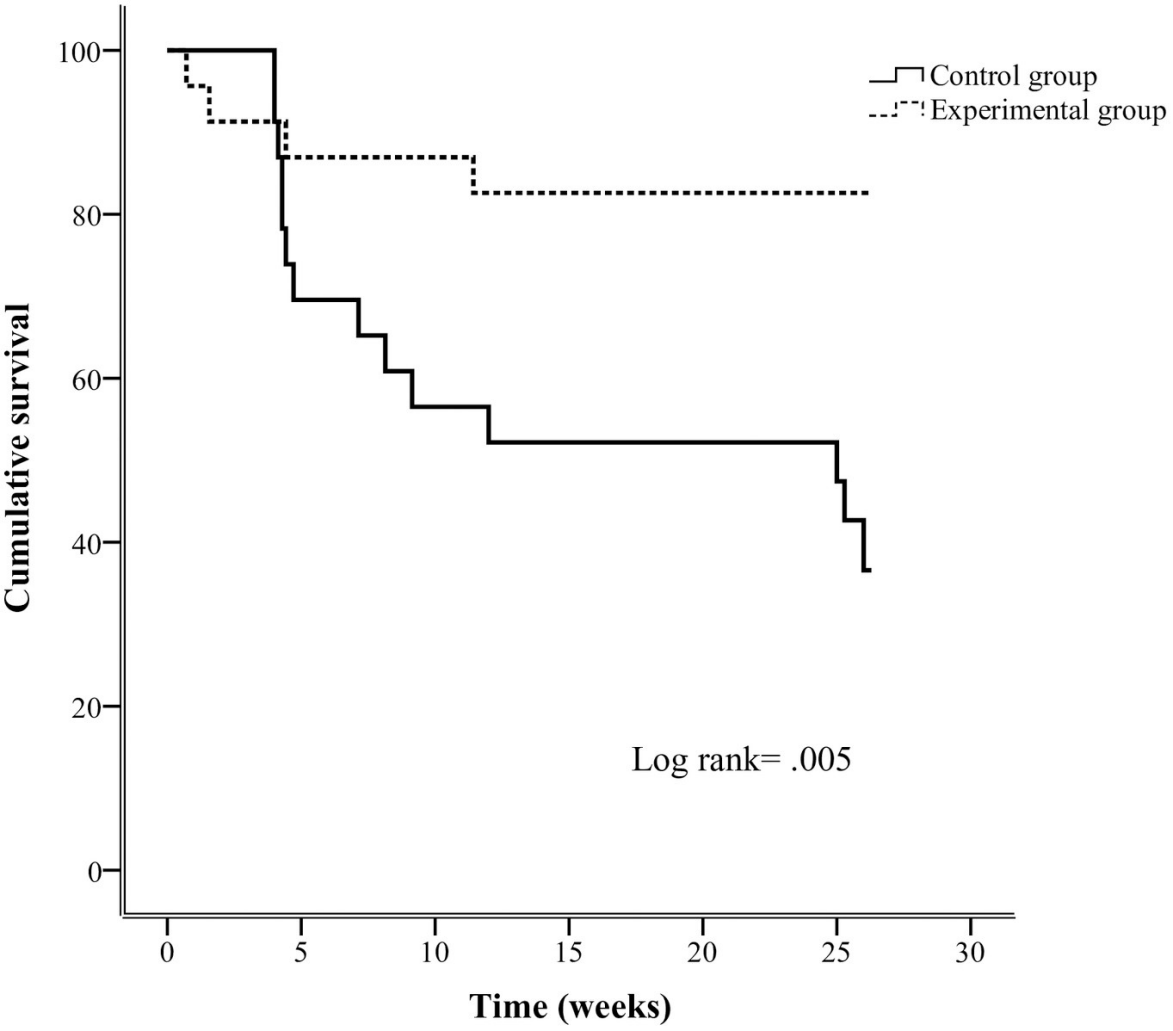
Kaplan-Meier plots for randomized groups on cumulative survival of foot ulcer recurrence over 6 months of follow-up in patients with high adherence to therapeutic footwear (N = 46).

Fig 5 shows the association between randomized groups and time to recurrence-free survival time (P = 0.005) in patients with high adherence to therapeutic footwear (N = 46).

Variables with P<0.1 in the univariate analysis (previous amputation and randomization) were included in the Cox multivariate model. The type of rocker sole was the only variable associated with ulcer recurrence-free survival time in the Cox multivariate model; use of rigid rocker soles was found to be a protective factor against recurrence development in diabetes patients with polyneuropathy and DFU history (P = 0.019; 95% confidence interval 0.086–0.807; hazard ratio 0.263).

## Discussion

Our results showed that compared with a semi-rigid rocker sole, a rigid rocker sole reduces the risk of recurrence in patients with previous history of plantar ulcer in the metatarsal heads. Based on the results of the RRR, patients who used a rigid sole had a 64% lower risk of developing a recurrence compared with patients who used a semi-rigid sole.

The incidence of plantar foot ulcer recurrence in the experimental group (23%) was similar to that found (27.7%) in a previous footwear trial performed by Uccioli L et al [11]. In this study they evaluated 69 patients considered at high risk of foot ulceration, but they excluded patients who underwent minor amputation. In our study, 64% of the control group patients developed recurrent ulcers; this might be due to deformities and previous minor amputations of the foot, which showed statistical significance in the univariate analysis; it is true that previous studies have found lower recurrence rates [35–37], but they did not include the type of therapeutic footwear used during the follow-up of those patients; thus, we believe that it is difficult to compare our results with others. We suggest the possible inclusion of rigid outsole shoes might contribute to the lower ulcer recurrence rates in previous studies.

Biomechanics of the foot is a factor related with the risk of reulceration. When comparing the same population with the same orthopedic treatment and background of DFU, the risk of developing a new event depends on the different patterns of amputation, which increases until 60% [38]. Patients with DFU below a metatarsal head are more difficult to offload because of severely altered biomechanics compared with other locations (e.g., ulcers on the tip and dorsum of the toes).

Plantar location, deformity records, and previous amputation can affect the biomechanics of our patients; consequently, orthopedic treatment could be challenging even using semi-rigid rocker soles.

Reints et al. [13] performed a cross-sectional study in which they excluded patients with diabetes and DFU history and analyzed plantar pressure in 30 healthy patients. They found that plantar pressure was lower in the rigid rocker sole group than in the semi-rigid sole group. Compared with this study, we obtained good results in terms of the reduction rate of plantar ulcers.

According to the recently published review of Armstrong et al. [1], 40% of patients who use a footwear demonstrated relief of plantar pressure and developed a recurrence in the first year after wound healing. In our study, recurrence rates of rigid outsole group were 23%. Selecting the kind of therapeutic shoe according to the risk of diabetic foot is a key point in the management of patients with diabetes. The selection of a therapeutic footwear should be determined based on classification of the patients according to the risk of developing a DFU [21]. Although further trials should confirm the benefits of dense outsole materials for reducing recurrent ulcers, we recommend the use of rigid soles in patients with DFU histories.

Our results should be interpreted with caution due to some limitations of the study. Unfortunately, the sample size was reduced in number than previously calculated, due to the difficulty in recruiting patients and the difficulty in finding the requirement in characteristic needed.

All of our patients had a low activity. As such, future studies should verify the efficacy of the rigid sole in a high-activity population. Finally, plantar pressure was not analyzed. However, mobility joint, deformities, history of previous minor amputation, and general variables were not significantly different between both groups. As such, we could assume that barefoot pressures in the sample were equivalent. Furthermore, our study did not analyze plantar pressure because we deemed that clinical outcomes are more important outcome measures.

The main strength of our study is that it is the first randomized clinical trial that clinically examined the ulcer recurrence rates of patients with diabetic foot and polyneuropathy in a follow-up period of 6 months in which different rocker sole materials are compared. We believe that the evaluation of high-risk patient is important when selecting the appropriate therapeutic footwear.

## Conclusions

A rigid rocker sole is better than a semi-rigid rocker sole in reducing ulcer recurrence in patients with diabetes, in those with history of previous ulcer in the plantar aspect of the foot, those with foot deformity, and those who underwent minor amputation.

Registration number Clinical trial reg. no.: NCT02995863

Name of trial registry

This Research Hypothesizes That the Use of a Rigid Rocker Sole Reduces the Recurrence Rate of Diabetic Foot Ulcers in Patients with Peripheral Neuropathy.

## Supporting information

S1 File Consort checklist. (PDF)

S2 File ClinicalTrials.gov protocol. Clinical Trial Registration Number: NCT02995863.
(PDF)

S3 File Local ethics committee. Short English version.
(PDF)

S4 File Local ethics committee. Long Spanish version.
(PDF)

S5 File Ethical approval certify. (PDF)

S6 File Study´s underlaying database. (SAV)
