# Supplementary material for: Clinical efficacy of therapeutic footwear with a rigid rocker sole in the prevention of recurrence in patients with diabetes mellitus and diabetic polineuropathy: A randomized clinical trial
Source: PLoS One. 2019 Jul 11;14(7):e0219537. doi: 10.1371/journal.pone.0219537 (PMC6623964; doi:10.1371/journal.pone.0219537)
Supplement: S2 File — Clinical Trial Registration Number: NCT02995863. (PDF) [file pone.0219537.s002.pdf]

ClinicalTrials.gov PRS
Protocol Registration and Results System

ClinicalTrials.gov Protocol Registration and Results System (PRS) Receipt
Release Date: May 10, 2018

ClinicalTrials.gov ID: NCT02995863

Study Identification

Unique Protocol ID: UComplutenseMadrid

Brief Title: This Research Hypothesizes That the Use of a Rigid Rocker Sole Reduces the Recurrence Rate of Diabetic Foot Ulcers in Patients With Peripheral Neuropathy.

Official Title: Clinical Efficacy of Therapeutic Footwear With a Rigid Rocker Sole in the Prevention of Reulceration in Patients With Diabetes Mellitus and Diabetic Polineuropathy: a Prospective and Randomized Clinical Trial.

Secondary IDs:

Study Status

Record Verification: May 2018

Overall Status: Completed

Study Start: December 10, 2016 [Actual]

Primary Completion: December 10, 2017 [Actual]

Study Completion: May 10, 2018 [Actual]

Sponsor/Collaborators

Sponsor: Universidad Complutense de Madrid

Responsible Party: Principal Investigator
Investigator: Mateo López Moral [malomo]
Official Title: Clinical Research
Affiliation: Universidad Complutense de Madrid

Collaborators:

Oversight

U.S. FDA-regulated Drug:

U.S. FDA-regulated Device:

U.S. FDA IND/IDE: No

Human Subjects Review: Board Status: Approved
Approval Number: 16/408-P
Board Name: CEIC Hospital Clínico San Carlos
Board Affiliation: Hospital Clínico San Carlos
Phone: 913303413
Email: ceic.hcsc@salud.madrid.org

Address:	Clínico San Carlos Hospital<br>Doctor Martín Lagos, s/n. Madrid.<br>28040<br>Madrid, España
Data Monitoring:	
FDA Regulated Intervention:	No

Study Description

Brief Summary:	This research presents a randomized clinical trial which analyzes the efficacy of a rigid rocker sole in the reduction of the recurrence rate of plantar ulcers in diabetic foot patients. The hypothesis of the research is the use of a rigid rocker sole reduces the recurrence rate of diabetic foot ulcers in patients with peripheral neuropathy.
Detailed Description:	

Conditions

Conditions:	Diabetic Foot<br>Diabetic Foot Ulcer
Keywords:	

Study Design

Study Type:	Interventional
Primary Purpose:	Prevention
Study Phase:	N/A
Interventional Study Model:	Parallel Assignment
Number of Arms:	2
Masking:	None (Open Label)
Allocation:	Randomized
Enrollment:	73 [Actual]

Arms and Interventions

Arms	Assigned Interventions
Experimental: Experimental Group<br>Rigid rocker sole footwear	Device: Rigid Rocker Sole Footwear
Active Comparator: Control Group<br>Therapeutic footwear	Device: Therapeutic Footwear

Outcome Measures

Primary Outcome Measure:

1. Presence of diabetic foot ulcer

The Wagner system assesses ulcer depth and the presence of osteomyelitis or gangrene using the following grades: grade 0 (pre- or post-ulcerative lesion), grade 1 (partial/full-thickness ulcer), grade 2 (probing to tendon or capsule), grade 3 (deep with osteitis), grade 4 (partial foot gangrene) and grade 5 (whole foot gangrene

[Time Frame: through study completion, an average of 6 months]

Secondary Outcome Measure:

2. Ankle Joint movility
   The ankle joint is examined in the neutral position, with the patient prone; a vertical line is marked on the patient's skin from heel to midcalf, and the maxi- mum range of dorsiflexion in passive motion is mea- sured in degrees with a goniometer. The normal value for the ankle joint is a mobility >90° of dorsi- flexion.

   [Time Frame: through study completion, an average of 6 months]

3. Mobility of the First metatarsal Joint
   Is examined with the patient in the supine position, and a horizontal line is drawn from the big toe to the heel. The maximum range of pas- sive dorsiflexion is recorded. The normal range of joint mobility is >65° at rest and 30° when the patient is standing.

   [Time Frame: through study completion, an average of 6 months]

4. Subtalar Joint Movements
   (inversion and eversion) are examined with the patient in a prone position, holding the calcaneus with one hand and the neck of the astragalus with the thumb and index finger of the other hand. Holding the astragalus rather than the tibia isolated the s

   [Time Frame: through study completion, an average of 6 months]

5. IPAQ (International Physical Activity Questionnaires)
   The purpose of the International Physical Activity Questionnaires (IPAQ) is to provide a set of well-developed instruments that can be used internationally to obtain comparable estimates of physical activity. There are two versions of the questionnaire. The short version is suitable for use in national and regional surveillance systems and the long version provide more detailed information often required in research work or for evaluation purposes.

   [Time Frame: through study completion, an average of 6 months]

6. Foot Type
   The validated protocol of the Foot Posture Index-6 involves the rating of three criteria in the rearfoot: Talar head palpation,Supra- and infra-lateral malleolar curvature and Calcaneal frontal plane position. In addition, there are three criteria on the forefoot: Prominence in the region of the talonavicular joint (TNJ), Congruence of the medial longitudinal arch (MLA), Abduction/adduction of the forefoot on the rearfoot. Each item is graded by a five-point Likert-type, from -2 to +2: 0 for neutral, with a minimum score of -2 for clear signs of supination, and +2 for positive signs of pronation. The final FPI-6 score will be a whole number between -12 and +12. A total FPI-6 score between 0 and +5 indicates a neutral foot, a score of above +6 indicates a pronated or highly pronated foot, and a score between -1 and -12 indicates a supinated or highly supinated foot.

   [Time Frame: through study completion, an average of 6 months]

7. Deformities
   Forefoot deformities were considered when the foot presented any of the following: hallux valgus, Tailor's bunion; toe contractures (hammer-toe, claw-toe or mallet-toe deformities); subluxation or dislocation of the metatarsophalangeal joints (overlapped toe and prominent metatarsal heads).

   [Time Frame: through study completion, an average of 6 months]

8. Ankle - Brachial Index (ABI)
   Were assessed by the same experienced podiatrist using a manual 8 MHz Doppler (Doppler II, Huntleigh Healthcare Ltd, South Glamorgan, UK), and the toe systolic pressure was taken with a digital plethysmography (Systoe, Atys Medical, Quermed, Madrid). The dorsalis pedis artery was used for recording ankle values. The ABI were calculated with the equations of the ankle pressure readings divided by the highest brachial reading between the right and left arms. We considered Peripheral Arterial Disease to have an ABI value less than 0.9; normal ABI values were between 0.9 and 1.39, and an ABI value ≥1.4 was considered poorly compressible vessels related to medial arterial calcification in distal arteries.

   [Time Frame: through study completion, an average of 6 months]

9. Sensorimotor neuropathy
Sensorimotor neuropathy was diagnosed by evaluation using a Semmes–Weinstein 5.07/10 g monofilament and a biothesiometer (both from Novalab Iberica, Madrid, Spain). Patients who did not feel 1 of the 2 tests were diagnosed with neuropathy.

[Time Frame: through study completion, an average of 6 months]

10. Physical Activity Questionnaire
Measurements about time of use of footwear either at home or outdoor. Normal value ranges are: never, 1 day per week, 1 to 3 day per week, 4 to 5 days per week and 6 to 7 days per week.

And measurements about how many hours per day the patient wear the footwear, the normal value ranges are: less than 1 hour, 1 to 3 hours per day, 4 to 7 hours per day, 8 to 11 hours per day and more than 12 hours per day.

[Time Frame: through study completion, an average of 6 months]

11. Toe - Brachial Index (TBI)
Were assessed by the same experienced podiatrist using a manual 8 MHz Doppler (Doppler II, Huntleigh Healthcare Ltd, South Glamorgan, UK), and the toe systolic pressure was taken with a digital plethysmography (Systoe, Atys Medical, Quermed, Madrid). The ABI and the TBI were calculated with the equations of the ankle or toe pressure readings divided by the highest brachial reading between the right and left arms. We considered PAD to have a TBI value less than 0.7; normal TBI values were between 0.7 and 0.99, and TBI ≥ 1 was considered distal arteries calcification.

[Time Frame: through study completion, an average of 6 months]

Eligibility

Minimum Age: 18 Years

Maximum Age:

Sex: All

Gender Based:

Accepts Healthy Volunteers: Yes

Criteria: Inclusion Criteria:

- Type 1 or 2 diabetic patients regardless of the pharmacotherapy that they receive.
- Adult patients of both sexes.
- Peripheral neuropathy patients.
- Patients without wounds or ulcers at the examination time.
- Patients with a foot ulcers history.
- Patients with or without minor amputation prior to the inclusion time in the present study.
- Patients who don´t need gait support mechanisms such as walking sticks, crutches, splints or any other devices which interferes with the autonomous development of the gait.

Exclusion Criteria:

- Patients with mayor amputation.
- Patients with rheumatic disease that affect the feet.
- Patients with peripheral neuropathy of different etiology to Diabetes mellitus.
- Patients with several critical ischemia criteria, defined by TACS II guideline.

Contacts/Locations

Central Contact Person: Mateo López Moral, 24
Telephone: 652311573 Ext. +34
Email: matlopezmor@hotmail.com

Central Contact Backup:

Study Officials:

Locations: Spain
Clínica Universitaria de Podología de la Universidad Complutense de Madrid
Madrid, Spain, 28040
Contact: Mateo López Moral, 24 652311573 Ext. +34
matlopezmor@hotmail.com

IPDSharing

Plan to Share IPD:

References

Citations:

Links:

Available IPD/Information:
