# Supplementary material for: Clinical efficacy of therapeutic footwear with a rigid rocker sole in the prevention of recurrence in patients with diabetes mellitus and diabetic polineuropathy: A randomized clinical trial
Source: PLoS One. 2019 Jul 11;14(7):e0219537. doi: 10.1371/journal.pone.0219537 (PMC6623964; doi:10.1371/journal.pone.0219537)
Supplement: S3 File — Short English version. (PDF) [file pone.0219537.s003.pdf]

Clinical efficacy of therapeutic footwear with a rigid rocker sole in the prevention of recurrence in patients with diabetes mellitus and diabetic polineuropathy: a randomized clinical trial.

Principal investigator: Prof. Dr. José Luis Lázaro Martínez

CLÍNICA UNIVERSITARIA DE PODOLOGÍA
UNIVERSIDAD COMPLUTENSE DE MADRID

Protocol version 01: September 2016

Hypothesis:

The use of rigid rocker sole reduces the recurrence rate of diabetic foot ulcers in patients with diabetes and peripheral neuropathy.

Aims:

Primary aim:

- To analyze the efficacy of a rigid rocker sole in the reduction of recurrence rate of plantar ulcers in patients with diabetic foot.

Secondary aims:

- To assess the wearing time and adherence to preventive treatment of patients with diabetes, similarly to the influence with the risk of recurrence.
- To identify general risk factors associated with a higher risk of recurrence.
- To identify biomechanical disorders associated with a higher risk of recurrence.

Research design and methods:

Study design:

Randomized and controlled parallel (1:1) clinical trial, intends to investigate the efficacy of a rigid rocker sole in the prevention of recurrence in patients with peripheral neuropathy and personal records of plantar ulceration.

Participants:

We are trying to include in the present study 138 patients with diabetes who come to the Diabetic Foot Unit of the University podiatrist Clinic of the Complutense University, Madrid, that fulfil the next criteria:

Inclusion criteria:

- Type 1 or type 2 patients with diabetes.
- Both sex patients Aged > 18 years.
- Peripheral neuropathy.
- No ulcer during examination.
- Previous foot ulcer under the metatarsal head.
- Patients with and without minor amputation before the inclusion of the present study.
- No need for walking aids.

Exclusion criteria:

- Transmetatarsal or major amputation (below or above the knee)
- History of rheumatoid disease.
- Other causes of neuropathy different to diabetes mellitus.
- Critical limb ischemia as defined by the TASC II guideline (88).

Sample size:

The calculations were based on the total patients with diabetes who will develop an ulcer throughout his life , which amounted to 15% (89) and it has been observed that the 48.2% of

the ulcers will develop in the plantar aspect of the foot (90). It was determined that, based a desired power of 80% with a β level of 20%, with an α level of 0.05, with a confidence interval of 95%, for a proportion of 50%, assuming a loss of 10%, at least 138 participants were included in the study

Allocation

The patients were randomly assigned to the control (semi-rigid rocker sole) or experimental (rigid rocker sole) group. Sample of rigid and semi rigid sole footwear are shown in first appendix, with model for both sexes. (83,85,86)
All patients will be tested to, neurological and vascular assessment according to International Guidelines.

Neurological Assessment

Loss of protective sensation was confirmed by:

- Inability to sense the pressure of a 10-g Semmes–Weinstein monofilament at four or more plantar foot sites (88, 91).
- Vibration perception threshold >25 V as assessed via the biothesiometer (92).

Vascular Assessment

Peripheral arterial disease (PAD) was define as:

- Absence of both distal pulses.
- Ankle brachial index (ABI) of <0.9 (88).
- Presión transcutánea de oxígeno ($TcpO_2$) inferior a 20 mmHg (93).

Once, the neurological and vascular assessment was performed, each patient was allocated to both different groups (control and experimental), in order to perform a posterior comparative analysis during the follow up of the study.

Methods:

All the patients, after the sign of the written informed consent (see appendix 2) will be asked for filiation and personal information, and data related to diabetes and its comorbidities.

A biomechanical exploration will be performed, data on ranges of mobility of the subtalar joint, ankle joint mobility, first metatarsophalangeal joint mobility in weigh bearing and non-weigh bearing position, foot posture index and hiperquerathosis pattern (94, 95, 96).

The ankle joint is examined in the neutral position, with the patient prone; a vertical line is marked on the patient's skin from heel to midcalf, and the maximum range of dorsiflexion in passive motion is measured in degrees with a goniometer. The normal value for the ankle joint is a mobility >90° of dorsi- flexion.

Subtalar Joint Movements, (inversion and eversion) are examined with the patient in a prone position, holding the calcaneus with one hand and the neck of the astragalus with the thumb and index finger of the other hand.

Mobility of the First metatarsal Joint is examined with the patient in the supine position, and a horizontal line is drawn from the big toe to the heel. The maximum range of passive dorsiflexion is recorded. The normal range of joint mobility is >65° at rest and 30° when the patient is standing.

Foot Type was measured using the validated protocol of the Foot Posture Index-6 involves the rating of three criteria in the rearfoot: Talar head palpation, Supra and infra-lateral malleolar curvature and Calcaneal frontal plane position. In addition, there are three criteria on the forefoot: Prominence in the region of the talonavicular joint (TNJ), Congruence of the medial longitudinal arch (MLA), Abduction/adduction of the forefoot on the rearfoot. Each item is graded by a five-point Likert-type, from -2 to +2: 0 for neutral, with a minimum score of -2 for clear signs of supination, and +2 for positive signs of pronation. The final FPI-6 score will be a whole number between -12 and +12. A total FPI-6 score between 0 and +5 indicates a neutral foot, a score of above +6 indicates a pronated or highly pronated foot, and a score between -1 and -12 indicates a supinated or highly supinated foot.

Forefoot deformities were considered when the foot presented any of the following: hallux valgus, Tailor's bunion; toe contractures (hammer-toe, claw-toe or mallet-toe deformities); subluxation or dislocation of the metatarsophalangeal joints (overlapped toe and prominent metatarsal heads).

Plantar pressure (PP) records will be performed in the standing position and in the dynamic during gait, through use of a computerized pedobarographic analysis system, with the system F – Scan ®, maximum plantar pressure, average plantar pressure and integral

pressure/time was recorded in 10 areas of the foot: medial heel, lateral heel, midfoot, metatarsal head from the first to fifth, first toe and average of the rest of the minor toes.

After the principal investigator makes sure the accordance of the therapeutic footwear, three different plantar pressure records will be made with the patient in the barefoot condition and three more with the therapeutic footwear (depends from the allocation). Before the PP records, patients will be instructed to walk along the laboratory and over the platform, to reproduce a normal gait for 5 minutes. (97)

All the patients will be instructed to complete an activity questionnaire of the last 7 days (see appendix 3). IPAQ (International Physical Activity Questionnaires): the purpose of the International Physical Activity Questionnaires (IPAQ) is to provide a set of well-developed instruments that can be used internationally to obtain comparable estmates of physical activity. There are two versions of the questionnaire. The short version is suitable for use in national and regional surveillance systems and the long version provide more detailed information often required in research work or for evaluation purposes. The questionnaire will be completed in each consultation and at the end of the follow-up period. (98).

In every visit, the principal investigator performed the debridement of high-risk points in the plantar forefoot areas, such as callus and hiperquerathosis, in addition the insole and the therapeutic footwear was checked, according to the International Working Group on the Diabetic Foot (99). All the patients included in the study will be followed up for six months (one visit each 30 ± 2 days) or until the development of a recurrence event.

Data record will be carried out by using the registration form that is attached in the appendix 4.

Univariate analyses for risk factors associated with ulcer location will be performed using Chi-square test for categorical variables and the Student t-test for quantitative variables using SPSS version 20.0 (SPSS, Chicago, IL, USA).

The strength of difference in the effect size will be calculated by Phi coefficient for chi-square test and r coefficient for non-parametric test considering the values >0.01 as a small effect, >0.30 as a medium effect, and >0.50 as a large effect. Cohen's d will be calculated as the effect size for parametric test using the effect size calculator (http://www.uccs.edu/~lbecker/), and considering the values >0.2, >0.5, and >0.8 as small, moderate, and large effects, respectively.

Variables that been significant at a p-value of ≤0.5 in the univariate analyses will be entered in the multivariate model using introduction selection, and the limit for entering or removing

variables was a p-value ≤0.05. The model will be controlled for potential confounding factors as well as for their possible associated interactions.

Relative risk reduction (RRR) and number needed to treat (NNT) will be used to estimate the size of the effect. A logistic regression model will be generated to estimate the risk of ulcer recurrence in both groups.

In summary, study variables are:

Main Outcome:

- Recurrence rate of plantar ulcers in patients with diabetes treated with therapeutic footwear with or without rigid sole.

Secondary outcomes:

- Association between the foot deformities with the risk of recurrence in patients with peripheral neuropathy

- Association between decreased ranges of mobility of the ankle joint, the subtalar joint and first metatarsophalangeal joint mobility with the risk of recurrence in patients with peripheral neuropathy.

- To define plantar pressure patterns associated with the risk of recurrence in the patient with diabetes and peripheral neuropathy, and the control of plantar pressure with the use of a rigid rocker sole.

- To define the wearing time of the therapeutic footwear by the patient with diabetes similarly to the impact on the risk of recurrent events and variables that affect in the wearing time.

Limitations of our study lie mainly in the selection bias of the patients, because of all the patients who come the Diabetic Foot Unit of the Complutense University of Madrid have a medical advice related with a pathological reason. Fewer of all the patients come to the Diabetic Foot Unit for prevention.

Research Schedule:

We intent to enroll 138 patients for the study in a period of 3 years and with a follow-up of 6 months per each patient.

The meeting schedule of the research team for the analyses of the outcomes and discussion of the reports produced will be as follows:

- Weekly meeting of the research team and the collaborating field researchers.
- Monthly review of the data collection forms and checks in the concordance in the SPSS v21.0 record.
- Three months later, statistical analyses to identify and asses the progress of the study and patients bias. A first publication will be published in two steps, first, preliminary outcomes and secondarily definitive results to compare with International Guidelines.
- After 6 months of the trial completion, a final report will be made with the following sections: research title, aims, research design and methods, discussion and conclusions as well as the financial justification.

The Project will be developed in three phases:

- First phase: Study of the neuropathic diabetic foot with medical history of diabetic foot ulcer on the plantar aspect.
- Second phase: to identify general and biomechanical disorders of patients with neuropathic diabetic foot and the relationship with the risk of recurrence.
- Third phase: to investigate the effectiveness of the therapeutic footwear with a rigid rocker sole in the reduction of the recurrence risk in plantar ulcers in patients with diabetes and diabetic foot.

(Monthly follow – up visits will be made for all the patients, from the moment each patient start wearing the therapeutic footwear and during the next 6 months. If the patient will develop a recurrence during the follow up period, it will be recorded in the variable of “recurrent event” to calculate the final recurrence rate).

Experience of the research team about the topic

The research team requests the next health aid funding is integrated in a Multidisciplinary Diabetic Foot Care team of the Diabetic Foot Unit of the University Complutense Clinic of Madrid.

The Diabetic Foot Unit of the Complutense University of Madrid in addition to the medical care, develops his work in a teaching activity with the Podiatrist Student of graduate, the training of fellows of postgraduate studies and the continuing training of different sanitary professionals in diabetic foot syndrome.

The Diabetic Foot Unit of the Complutense University of Madrid is a member of the National Scientific Association named as "Multidisciplinary research study of the Diabetic Foot", which actively participate in meetings to improve the assistance care of Diabetic Foot Syndrome in our country and reduce the number of amputations which is associated with dead in these patients with diabetes mellitus.

In the specific field of the diabetic foot, research team have already published more than 40 scientific journals in the last five years, in international high impact journals, on the other hand they have published preliminary studies about this topic in national and international congress and finally realized PhD thesis. Furthermore, they develop their medical assistance in the Diabetic Foot Unit of the Complutense University of Madrid.

Some of our Scientific Journals:

2016:

- ✓ Molines-Barroso RJ, Lázaro-Martínez JL, Álvaro-Afonso FJ, Sanz-Corbalán I, García-Klepzig JL, Aragón-Sánchez J. Validation of an algorithm to predict reulceration in amputation patients with diabetes. Int Wound J. 2016 Aug 9. doi: 10.1111/iwj.12639. JCR (2015) Impact factor 2,59. Quartil: Q1, Ranking: 13 of 61. Category: Dermatología.

- ✓ Molines-Barroso RJ, Lázaro-Martínez JL, Aragón-Sánchez FJ, Álvaro-Afonso FJ, García-Morales E, García-Álvarez Y. Forefoot ulcer risk is associated with foot type in patients with diabetes and neuropathy. Diabetes Res Clin Pract. 2016 Jan 14. pii:

S0168-8227(16)00057-7. doi: 10.1016/j.diabres.2016.01.008. JCR (2015) Impact factor : 3,04. Quartil: Q2, Ranking: 63 of 131. Category: Endocrinología y Metabolismo.

2015:

- ✓ Álvaro-Afonso FJ, Lázaro-Martínez JL, Aragón-Sánchez J, García-Morales E, García-Álvarez Y, Molines-Barroso RJ. What Is the Clinical Utility of the Ankle-Brachial Index in Patients With Diabetic Foot Ulcers and Radiographic Arterial Calcification? Int J Low Extrem Wounds. 2015 Dec;14(4):372-6. JCR (2015) Impact factor: 1,36. Quartil: Q3, Ranking: 39 of 61. Category: Dermatología.

- ✓ Sanz-Corbalán I, Lázaro-Martínez JL, Aragón-Sánchez J, García-Morales E, Molines-Barroso R, Alvaro-Afonso FJ. Analysis of Ulcer Recurrences After Metatarsal Head Resection in Patients Who Underwent Surgery to Treat Diabetic Foot Osteomyelitis. Int J Low Extrem Wounds. 2015 Jun;14(2):154-9. JCR (2015) Impact factor: 1,36. Quartil: Q3, Ranking: 39 of 61. Category: Dermatología.

Aragón-Sánchez J, Lázaro-Martínez JL, Alvaro-Afonso FJ, Molinés-Barroso R. Conservative Surgery of Diabetic Forefoot Osteomyelitis: How Can I Operate on This Patient Without Amputation? Int J Low Extrem Wounds. 2015 Jun;14(2):108-31. JCR (2015) Impact factor : 1,36. Quartil: Q3, Ranking: 39 of 61. Category: Dermatología.

2014:

- ✓ Armstrong DG, Hanft JR, Driver VR, Smith AP, Lazaro-Martinez JL, Reyzelman AM, Furst GJ, Vayser DJ, Cervantes HL, Snyder RJ, Moore MF, May PE, Nelson JL, Baggs GE, Voss AC; the Diabetic Foot Nutrition Study Group. Effect of oral nutritional supplementation on wound healing in diabetic foot ulcers: a prospective randomized controlled trial. Diabet Med. 2014 May 28. doi: 10.1111/dme.12509. [Epub ahead of print]. JCR (2015) Impact factor :3,15. Quartil: Q2, Ranking: 56 of 131. Category: Endocrinología y Metabolismo.

- ✓ Alvaro-Afonso FJ, Lázaro-Martínez JL, Aragón-Sánchez J, García-Morales E, García-Álvarez Y, Molines-Barroso RJ. Inter-observer reproducibility of diagnosis of diabetic foot osteomyelitis based on a combination of probe-to-bone test and simple radiography. Diabetes Res Clin Pract. 2014 Jul; 105(1):e3-5. doi: 10.1016/j.diabres.2014.04.024. Epub 2014 Apr 28. JCR (2015) Impact factor 3,04. Quartil: Q2, Ranking: 63 of 131. Category: Endocrinología y Metabolismo.

- ✓ Lázaro-Martínez JL, Aragón-Sánchez J, García-Morales E. Response to comment on Lazaro-Martinez et al. Antibiotics versus conservative surgery for treating diabetic foot osteomyelitis: a randomized comparative trial. Diabetes Care 2014; 37:789-795. Diabetes Care. 2014 May; 37(5):e116-7. doi: 10.2337/dc14-0114. JCR (2015) Impact factor: 8,93. Quartil: Q1, Ranking: 7 of 131. Category: Endocrinología y Metabolismo.

- ✓ Aragón-Sánchez J, Lázaro-Martínez JL, García-Álvarez Y, Morales EG, Hernández-Herrero MJ. Albuminuria is a predictive factor of in-hospital mortality in patients with diabetes admitted for foot disease. Diabetes Res Clin Pract. 2014 Jan 21. pii: S0168-8227(14)00008-4. doi: 10.1016/j.diabres.2014.01.006. JCR (2015) Impact factor: 3,04. Quartil: Q2, Ranking: 63 of 131. Category: Endocrinología y Metabolismo.

- ✓ Lázaro-Martínez J, Aragón-Sánchez J, García-Morales E. Antibiotics versus conservative surgery for treating diabetic foot osteomyelitis. A randomized comparative trial. Diabetes Care.2014 Mar; 37(3):789-95. JCR (2015) Impact factor: 8,93. Quartil: Q1, Ranking: 7 of 131. Category: Endocrinología y Metabolismo.

2013:

- ✓ Cecilia-Matilla A, Lázaro-Martínez JL, Aragón-Sánchez J, García-Morales E, García-Álvarez Y, Beneit-Montesinos JV. Histopathologic characteristics of bone infection complicating foot ulcers in diabetic patients. J Am Podiatr Med Assoc. 2013 Jan-Feb; 103(1):24-31. JCR (2015) Impact factor : 0,48. Quartil: Q4, Ranking: 68 of 74. Category: Ortopedia.

- ✓ Sanz-Corbalán I, Lázaro-Martínez JL, García-Morales E, Aragón-Sánchez J, Carabantes-Alarcón D, García-Álvarez Y. Relationship of limited joint mobility and foot deformities with neurological examination in patients with diabetes. Exp Clin Endocrinol Diabetes. 2013 Apr; 121(4):239-43. doi: 10.1055/s-0032-1329981. Epub 2013 Jan 17. JCR (2015) Impact factor :1,66. Quartil: Q4, Ranking: 105 of 131. Category: Endocrinología y Metabolismo.

- ✓ Álvaro-Afonso FJ, Lázaro-Martínez JL, Aragón-Sánchez J, García-Morales E, Cecilia-Matilla A, Beneit-Montesinos JV. Interobserver and intraobserver reproducibility of plain X-rays in the diagnosis of diabetic foot osteomyelitis. Int J Low Extrem Wounds. 2013 Mar; 12(1):12-5. Doi: 10.1177/1534734612474304. Epub 2013 Feb 1. JCR (2015) Impact factor : 1,36. Quartil: Q3, Ranking: 39 of 61. Category: Dermatología.

- ✓ Aragón-Sánchez J, Lázaro-Martínez JL, Quintana-Marrero Y, Sanz-Corbalán I, Hernández-Herrero MJ, Cabrera-Galván JJ. Super-oxidized solution (dermacyn wound care) as adjuvant treatment in the postoperative management of complicated diabetic foot osteomyelitis: preliminary experience in a specialized department. Int J Low Extrem Wounds. 2013 Jun; 12(2):130-7. Doi: 10.1177/1534734613476710. Epub 2013 Feb 26. JCR (2015) Impact factor: 1,36. Quartil: Q3, Ranking: 39 of 61. Category: Dermatología.

- ✓ Aragón-Sánchez J, Lipsky BA, Lázaro-Martínez JL. Gram-negative diabetic foot osteomyelitis: risk factors and clinical presentation. Int J Low Extrem Wounds. 2013 Mar; 12(1):63-8. Doi: 10.1177/1534734613477423. Epub 2013 Feb 26. JCR (2015)Impact factor: 1,36. Quartil: Q3, Ranking: 39 of 61. Category: Dermatología.

- ✓ Aragón-Sánchez J, Lázaro-Martínez JL, Quintana-Marrero Y, Alvaro-Afonso FJ, Hernández-Herrero MJ. Charcot neuroarthropathy triggered and complicated by osteomyelitis. How limb salvage can be achieved. Diabet Med. 2013 Jun; 30(6):e229-32.

doi: 10.1111/dme.12191. Epub 2013 Apr 17. JCR (2015) Impact factor :3,15. Quartil: Q2, Ranking: 56 of 131. Category: Endocrinología y Metabolismo.

- ✓ Molines-Barroso RJ, Lázaro-Martínez JL, Aragón-Sánchez J, García-Morales E, Beneit-Montesinos JV, Alvaro-Afonso FJ. Analysis of transfer lesions in patients who underwent surgery for diabetic foot ulcers located on the plantar aspect of the metatarsal heads. Diabet Med. 2013 Aug; 30(8):973-6. Doi: 10.1111/dme.12202. Epub 2013 Apr 20. JCR (2015) Impact factor :3,15. Quartil: Q2, Ranking: 56 of 131. Category Endocrinología y Metabolismo.

- ✓ Aragón-Sánchez J, Lázaro-Martínez JL, Molinés-Barroso R, García Álvarez Y, Quintana-Marrero Y, Hernández-Herrero MJ. Revision surgery for diabetic foot infections: giving another chance to the patient. Int J Low Extrem Wounds. 2013 Jun; 12(2):146-51. Doi: 10.1177/1534734613486155. Epub 2013 May 13. JCR (2015) Impact factor : 1,36. Quartil: Q3, Ranking: 39 of 61. Category: Dermatología.

- ✓ García-Álvarez Y, Lázaro-Martínez JL, García-Morales E, Cecilia-Matilla A, Aragón-Sánchez J, Carabantes-Alarcón D. Morphofunctional characteristics of the foot in patients with diabetes mellitus and diabetic neuropathy. Diabetes Metab Syndr. 2013 Apr-Jun; 7(2):78-82. doi: 10.1016/j.dsx.2013.02.029. Epub 2013 Mar 15.

- ✓ Cecilia-Matilla A, Lázaro-Martínez JL, Aragón-Sánchez J. Dear Editor. J Foot Ankle Surg. 2013 Jun 12. Doi: pii: S1067-2516(13)00196-8. 10.1053/j.jfas.2013.05.003. JCR (2015) Impact factor : 1,09. Quartil: Q3, Ranking: 49 of 74. Category: Ortopedia.

- ✓ Alvaro-Afonso FJ, Lázaro-Martínez JL, Aragón-Sánchez FJ, García-Morales E, Carabantes-Alarcón D, Molines-Barroso RJ. Does the location of the ulcer affect the interpretation of the probe-to-bone test in the diagnosis of osteomyelitis in diabetic foot ulcers? Diabet Med. 2013 Jul 19. Doi: 10.1111/dme.12280. JCR (2015) Impact factor :3,15. Quartil: Q2, Ranking: 56 of 131. Category: Endocrinología y Metabolismo.

- ✓ Aragón-Sánchez J, Lázaro-Martínez JL, Cabrera-Galván JJ. Additional information on the role of histopathology in diagnosing diabetic foot osteomyelitis. Diabet Med. 2013 Jul 22. Doi: 10.1111/dme.12283. JCR (2015) Impact factor :3,15. Quartil: Q2, Ranking: 56 of 131. Category: Endocrinología y Metabolismo.

- ✓ Cecilia-Matilla A, Lázaro-Martínez JL, Aragón-Sánchez J, García-Álvarez Y, Chana-Valero P, Beneit-Montesinos JV. Influence of the Location of Nonischemic Diabetic Forefoot Osteomyelitis on Time to Healing After Undergoing Surgery. Int J Low Extrem Wounds. 2013 Sep 16. JCR (2015) Impact factor 1,36. Quartil: Q3, Ranking: 39 of 61. Category: Dermatología.

- ✓ Rubio JA, Aragón-Sánchez J, Lázaro-Martínez JL, Almaraz MC, Mauricio D, Antolín Santos JB, Díaz Pérez JA, Fabbi M, Lozano Del Hoyo ML, Vela MP; en representación del Grupo Español del Pie Diabético. Diabetic foot units in Spain: Knowing the facts using a questionnaire. Endocrinol Nutr. 2013 Nov 4. Doi: pii: S1575-0922(13)00269-6. 10.1016/j.endonu.2013.07.002. English, Spanish.

- ✓ Aragón-Sánchez J, Lázaro-Martínez JL. Factors Associated With Calcification in the Pedal Arteries in Patients With Diabetes and Neuropathy Admitted for Foot Disease and Its Clinical Significance. Int J Low Extrem Wounds. 2013 Nov 25. JCR (2015) Impact factor 1,36. Quartil: Q3, Ranking: 39 of 61. Category: Dermatología.

- ✓ Lázaro-Martínez JL, Cecilia-Matilla A, Aragón-Sánchez J, García-Morales E, Garcia-Alvarez Y, Alvaro-Afonso F. Treatment of infected diabetic foot ulcers clinical effectiveness of a dressing of alginate and hydrocolloid, with silver fiber. Analysis of results of a series of cases. Rev Enferm. 2013 Nov; 36(11):29-34.

2012:

- ✓ Aragón-Sánchez J, Lázaro-Martínez JL, Hernández-Herrero C, Campillo-Vilorio N, Quintana-Marrero Y, García-Morales E, Hernández-Herrero MJ. Does osteomyelitis in the feet of patients with diabetes really recur after surgical treatment? Natural history of a surgical series. Diabet Med. 2012 Jun;29(6):813-8. doi: 10.1111/j.1464-5491.2011.03528.x. JCR (2015) Impact factor :3,15. Quartil: Q2, Ranking: 56 of 131. Category: Endocrinología y Metabolismo.

- ✓ Aragón-Sánchez J, Lázaro-Martínez JL, Cecilia-Matilla A, Quintana-Marrero Y, Hernández-Herrero MJ. Limb salvage for spreading midfoot osteomyelitis following diabetic foot surgery. J Tissue Viability. 2012 May;21(2):64-70. Epub 2012 Jan 9. JCR (2015) Impact factor:: 1,65. Quartil: Q1, Ranking: 19 of 116. Category: Enfermería.

- ✓ García-Morales E, Lázaro-Martínez JL, Aragón-Sánchez J, Cecilia-Matilla A, García-Álvarez Y, Beneit-Montesinos JV. Surgical complications associated with primary closure in patients with diabetic foot osteomyelitis. Diabet Foot Ankle. 2012; 3. Doi: 10.3402/dfa.v3i0.19000. Epub 2012 Sep 25.

- ✓ Aragón-Sánchez J, Lázaro-Martínez JL, Pulido-Duque J, Maynar M. From the diabetic foot ulcer and beyond: how do foot infections spread in patients with diabetes? Diabet Foot Ankle. 2012;3. doi: 10.3402/dfa.v3i0.18693. Epub 2012 Oct 1.

- ✓ Aragón-Sánchez J, Lázaro-Martínez JL, Campillo-Vilorio N, Quintana-Marrero Y, Hernández-Herrero MJ. Controversies regarding radiological changes and variables predicting amputation in a surgical series of diabetic foot osteomyelitis. Foot Ankle Surg. 2012 Dec;18(4):233-6. doi: 10.1016/j.fas.2012.01.005. Epub 2012 Mar 2. JCR (2015) Impact factor: 1,09. Quartil: Q3, Ranking: 49 of 74. Category: Ortopedia.

2011:

- ✓ Aragon-Sanchez J, Lipsky BA, Lazaro-Martinez JL. Diagnosing diabetic foot osteomyelitis: is the combination of probe-to-bone test and plain radiography sufficient for high-risk inpatients? Diabet Med. 2011 Feb;28(2):191-4. JCR (2015) Impact factor :3,15. Quartil: Q2, Ranking: 56 of 131. Category: Endocrinología y Metabolismo.

- ✓ Garcia-Morales E, Lazaro-Martinez JL, Martinez-Hernandez D, Aragon-Sanchez J, Beneit-Montesinos JV, Gonzalez-Jurado MA. Impact of diabetic foot related complications on the Health Related Quality of Life (HRQol) of patients--a regional study in Spain. Int J Low Extrem Wounds. 2011 Mar;10(1):6-11. JCR (2015) Impact factor: 1,36. Quartil: Q3, Ranking: 39 of 61. Category: Dermatología.

- ✓ Lazaro-Martinez JL, Aragon-Sanchez FJ, Beneit-Montesinos JV, Gonzalez-Jurado MA, Garcia Morales E, Martinez Hernandez D. Foot biomechanics in patients with diabetes mellitus: doubts regarding the relationship between neuropathy, foot motion, and deformities. J Am Podiatr Med Assoc. May-Jun;101(3):208-14. JCR (2015) Impact factor: 0,48. Quartil: Q4, Ranking: 68 of 74. Category: Ortopedia

- ✓ Garcia Morales E, Lazaro-Martinez JL, Aragon-Sanchez FJ, Cecilia-Matilla A, Beneit-Montesinos JV, Gonzalez Jurado MA. Inter-observer reproducibility of probing to bone in

the diagnosis of diabetic foot osteomyelitis. Diabet Med. 2011 Oct;28(10):1238-40. JCR (2015) Impact factor :3,15. Quartil: Q2, Ranking: 56 of 131. Category: Endocrinología y Metabolismo.

- ✓ Aragon-Sanchez J, Lazaro-Martinez JL. Comment on: Lipsky et al. Developing and validating a risk score for lower-extremity amputation in patients hospitalized for a diabetic foot infection. Diabetes Care 2011;34:1695-1700. Diabetes Care. Oct;34(10):e160; author reply e1. JCR (2015) Impact factor : 8,93. Quartil: Q1, Ranking: 7 of 131. Category: Endocrinología y Metabolismo.
- ✓ Aragon-Sanchez J, Jimenez FC, Lazaro-Martinez JL, Campillo-Vilorio N, Quintana-Marrero Y, Hernandez-Herrero MJ. Never Amputate a Patient With Diabetes Without Consulting With a Specialized Unit. Int J Low Extrem Wounds. 2011 Dec;10(4):214-7. JCR (2015) Impact factor : 1,36. Quartil: Q3, Ranking: 39 of 61. Category: Dermatología.

- ✓ Aragon-Sanchez J, Lazaro-Martinez JL. Impact of perioperative glycaemia and glycated haemoglobin on the outcomes of the surgical treatment of diabetic foot osteomyelitis. Diabetes Res Clin Pract. 2011 Dec; 94(3):83-5. JCR (2015) Impact factor: 3,04. Quartil: Q2, Ranking: 63 of 131. Category: Endocrinología y Metabolismo.

- ✓ Aragon-Sanchez J, Lazaro-Martinez JL, Hernandez-Herrero C, Campillo-Vilorio N, Quintana-Marrero Y, Garcia-Morales E, et al. Surgical Treatment of Limb- and Life-Threatening Infections in the Feet of Patients With Diabetes and at Least One Palpable Pedal Pulse: Successes and Lessons Learnt. Int J Low Extrem Wounds. 2011 Dec;10(4):207-13. JCR (2015) Impact factor: 1,36. Quartil: Q3, Ranking: 39 of 61. Category: Dermatología.

- ✓ Aragon-Sanchez J, Lipsky BA, Lazaro-Martinez JL. Diagnosing diabetic foot osteomyelitis: is the combination of probe-to-bone test and plain radiography sufficient for high-risk inpatients? Diabet Med. 2011 Feb;28(2):191-4. JCR (2015) Impact factor :3,15. Quartil: Q2, Ranking: 56 of 131. Category: Endocrinología y Metabolismo.

- ✓ Garcia-Morales E, Lazaro-Martinez JL, Martinez-Hernandez D, Aragon-Sanchez J, Beneit-Montesinos JV, Gonzalez-Jurado MA. Impact of diabetic foot related complications on the Health Related Quality of Life (HRQol) of patients--a regional study in Spain. Int J Low Extrem Wounds. 2011 Mar;10(1):6-11. JCR (2015) Impact factor : 1,36. Quartil: Q3, Ranking: 39 of 61. Category: Dermatología.

- ✓ Lazaro-Martinez JL, Aragon-Sanchez FJ, Beneit-Montesinos JV, Gonzalez-Jurado MA, Garcia Morales E, Martinez Hernandez D. Foot biomechanics in patients with diabetes mellitus: doubts regarding the relationship between neuropathy, foot motion, and deformities. J Am Podiatr Med Assoc. May-Jun;101(3):208-14. JCR (2015) Impact factor: 0,48. Quartil: Q4, Ranking: 68 of 74. Category: Ortopedia

- ✓ Aragon-Sanchez J, Jimenez FC, Lazaro-Martinez JL, Campillo-Vilorio N, Quintana-Marrero Y, Hernandez-Herrero MJ. Never Amputate a Patient With Diabetes Without Consulting With a Specialized Unit. Int J Low Extrem Wounds. 2011 Dec;10(4):214-7. JCR (2015) Impact factor : 1,36. Quartil: Q3, Ranking: 39 of 61. Category: Dermatología.

- ✓ Aragon-Sanchez J, Lazaro-Martinez JL, Hernandez-Herrero C, Campillo-Vilorio N, Quintana-Marrero Y, Garcia-Morales E, et al. Surgical Treatment of Limb- and Life-Threatening Infections in the Feet of Patients With Diabetes and at Least One Palpable Pedal Pulse: Successes and Lessons Learnt. Int J Low Extrem Wounds. 2011 Dec;10(4):207-13. JCR (2015) Impact factor: 1,36. Quartil: Q3, Ranking: 39 of 61. Category: Dermatología.

2010:

- ✓ Lazaro-Martinez JL, Garcia-Morales EA, Aragon-Sanchez FJ, Ano-Vidales P, Allas-Aguado S, Garcia-Alvarez Y. [Reducing skin maceration in exudative diabetic foot ulcers]. Rev Enferm. 2010 Mar;33(3):9-14.

- ✓ Aragon-Sanchez J, Hernandez-Herrero MJ, Lazaro-Martinez JL, Quintana-Marrero Y, Maynar-Moliner M, Rabellino M, et al. In-hospital complications and mortality following major lower extremity amputations in a series of predominantly diabetic patients. Int J Low Extrem Wounds. 2010 Mar;9(1):16-23. JCR (2015) Impact factor: 1,36. Quartil: Q3, Ranking: 39 of 61. Category: Dermatología.

- ✓ Aragon-Sanchez J, Lazaro-Martinez JL, Hernandez-Herrero MJ, Quintana-Marrero Y, Cabrera-Galvan JJ. Clinical significance of the isolation of Staphylococcus epidermidis from bone biopsy in diabetic foot osteomyelitis. Diabet Foot Ankle. 2010.1 doi: 10.3402/dfa.v1i0.5418.

✓ Aragón-Sánchez J, Lázaro-Martínez JL, Hernández-Herrero MJ. Triggering mechanisms of neuroarthropathy following conservative surgery for osteomyelitis. Diabet Med. 2010 Jul;27(7):844-7. JCR (2015) Impact factor :3,15. Quartil: Q2, Ranking: 56 of 131. Category: Endocrinología y Metabolismo.

At present, they develop the next research areas:

1. “Diabetic foot osteomyelitis”
2. “Biomechanical disorders in patients with and without diabetic neuropathy"
3. “Biomechanical study and the risk of reulceration in patients with diabetes with and without neuropathy through the analyses of podiatrist pathology”.
4. “Study of classification of prognosis factors in the healing of diabetic foot ulcers”
5. “Efficacy of micro – current in the healing of neuroischemic ulcers in the diabetic foot”
6. “Efficacy and effectiveness of different plantar orthosis in the reduction of plantar pressures”

The next PhD thesis have been published result of these research areas:

- Jose Luis Lázaro Martínez: “Determinación de las Características Biomecánicas en el pie Diabético con y sin neuropatía”.

- María Luz González Fernández: “Estudio del tratamiento ortopodológico individualizado del paciente diabético de alto riesgo y su efecto sobre la incidencia de la reulceración”.

- María del Rosario Morales Lozano: “Validación de la prueba del probing to bone frente a otras pruebas de diagnóstico clínico de la osteomielitis crónica en el pie diabético”.

- Gabriel Rivera: “Estudio de la anatomía osteoarticular del pie mediante el análisis goniométrico en radiografía simple en pacientes diabéticos con y sin neuropatía”.

- Laura Ramos: “Estudio de la influencia de la limitación de la movilidad articular en el desarrollo de patrones de sobrecarga en el pie diabético con y sin neuropatía”.

- Juan Pedro Sánchez Ríos: **"**Eficacia de los Ácidos Grasos Hiperoxigenados en la microcirculación de pacientes con Pie Diabético**”**

- Almudena Cecilia Matilla: “Determinación histopatológica de la osteomielitis y su influencia en el pronóstico de la cicatrización en pacientes con úlceras de pie diabético”

- Yolanda García Álvarez: “Identificación de las deformidades morfofuncionales del pie en pacientes diabéticos con o sin neuropatía y en pacientes no diabéticos mediante el análisis de la patología podológica y biomecánica”.

- Francisco Javier Álvaro Afonso: “Variabilidad diagnóstica de la palpación transulcerosa de hueso (Probe to bone) y de la radiografía simple en el diagnóstico de osteomielitis en úlceras de pie diabético”.

- Silvia Allas Aguado: “Estudio clínico aleatorizado sobre la efectividad del tratamiento médico frente al quirúrgico en la osteomielitis asociada a lesiones de pie diabético”.

- Raúl Juan Molines Barroso: “Riesgo de reulceración tras la resección quirúrgica de una cabeza metatarsal en el paciente con Diabetes mellitus”.

- Javier Hernández Toledo: “Las bacterias Gram negativas y su influencia en la cicatrización de úlceras de pie diabético”.

- Irene Sanz Corbalán: “Utilidad del test de la función sudomotora como instrumento clínico de clasificación de riesgo del paciente diabético”.

Podartis® Lab is actually one of main important manufacturing companies in therapeutic footwear in the European Union. The extensive experience, the capacity of their storage facilities to develop all the manufacturing process, and the human team have become a reference in the field of the international research in prevention of diabetic foot ulcer with therapeutic footwear.

Plan for dissemination:

1.- Importance of the research regarding to clinical and assistance impact and/or technological development

The feasibility of the project will enable to get clinical objectives in the prevention of the diabetic foot field, due to define risk factors associated with biomechanical disorders in patients with diabetes, which results in the high plantar pressures and the development of diabetic foot ulcers. This research will enable us to determine preventive treatments that will contribute us to reduce the reulceration and amputation rates, which is a big social issue in the present society.

The aim proof of the benefits about podiatrist technologies in the diabetic foot care is relevant in order to favor the development of Multidisciplinary Diabetic Foot Units in our country.

2.- Bibliometric impact of the research

Diabetic Foot Syndrome is a health issue that promote numerous studies. Many research about diabetic foot have been published previously in: Lancet, Diabetes Care, Diabetes Medicine, Clinical Biomechanics, Foot and Ankle International y JAPMA (Journal American Podiatric Medical Association).

Many studies talk about prevention of the reulceration in the diabetic foot, but none have been specific about how to prevent the reulceration of the diabetic foot with the use of a rigid rocker sole.

Available resources for project implementation

Diabetic Foot Unit of the Podiatrist Clinic of the Complutense University of Madrid has the next physic resources to the Development of the next research:

- Room specially prepared for research whit the next tools for neurological assessment (Semmes Weinstein Monofilament and Tuning Fork), Vascular assessment (computerized doppler and transcutaneous oxygen pressure instrument).
  Furthermore, the research room has walking bench, examining table and podoscopio.

- The biomechanical assessment of the patient with diabetes requires an investment in time and economic resources. The Diabetic Foot Unit provide all equipment needed.

Podartis® Laboratory has different models of therapeutic footwear for man (X-Diab-14) and woman (Cortina Gran Volume), which only differ in the outsole design, while the rest of characteristics of the shoe are the same in both groups (see appendix 1).
Their products are clinically tested to ensure the maximum benefit and comfort. All the models are available in Spain from 48 to 72 hours from their order.
Podartis® is specialized in the footwear production for the prevention of the diabetic foot ulceration and is an internationally benchmark company, with a high experience since its foundation in 2001.

Bibliography:

1. Conget I. [Diagnosis, classification and cathogenesis of diabetes mellitus]. Rev Esp Cardiol. 2002 May;55(5):528-35.
2. Boulton AJ, Vileikyte L, Ragnarson-Tennvall G, Apelqvist J. The global burden of diabetic foot disease. Lancet. 2005 Nov 12;366(9498):1719-24.
3. Wild S, Roglic G, Green A, Sicree R, King H. Global prevalence of diabetes: estimates for the year 2000 and projections for 2030. Diabetes Care. 2004 May;27(5):1047-53.
4. Singh N, Armstrong DG, Lipsky BA. Preventing foot ulcers in patients with diabetes. JAMA. 2005 Jan 12;293(2):217-28.
5. Instituto Nacional de Salud. Encuesta Nacional de Salud2006.
6. Delgado E. Las estimaciones internacionales indican que la prevalencia de diabetes se duplicará en los próximos años. Economía de la Salud. 2008;7(2):55-8.
7. Hogan P, Dall T, Nikolov P. Economic costs of diabetes in the US in 2002. Diabetes Care. 2003 Mar;26(3):917-32.
8. Ballesta M, Carral F, Grupo de diabetes de la SAEN. Costes directos e indirectos de la diabetes mellitus tipo 2. Avances en Diabetes. 2002;18 Supl(1):20.
9. Conget I. Un control exhaustivo de la diabetes reduce los costes asociados a las complicaciones. Economía de la Salud 2008.7(2):88.
10. O'Sullivan JB, Mahan CM. Mortality related to diabetes and blood glucose levels in a community study. Am J Epidemiol. 1982 Oct;116(4):678-84.
11. Frykberg RG. Epidemiology of the diabetic foot: ulcerations and amputations. Adv Wound Care. 1999 Apr;12(3):139-41.
12. Organización Mundial de la Salud. Prevención de la diabetes mellitus. Ginebra: Informe de un Grupo de Estudio de la OMS. Ser Inf Tecn. 1994;884:81.
13. Moss SE, Klein R, Klein BE. The 14-year incidence of lower-extremity amputations in a diabetic population. The Wisconsin Epidemiologic Study of Diabetic Retinopathy. Diabetes Care. 1999 Jun;22(6):951-9.
14. Carmona GA, Hoffmeyer P, Herrmann FR, Vaucher J, Tschopp O, Lacraz A, et al. Major lower limb amputations in the elderly observed over ten years: the role of diabetes and peripheral arterial disease. Diabetes Metab. 2005 Nov;31(5):449-54.
15. Glober JL, Weingarten MS, Buchbinder DS. A 4-year outcome-based retrospective study of wound healing and limb salvage in patients with chronic wounds. Adv Wound Care. 1997;10:33-7.

16. Levin ME. Preventing amputation in the patient with diabetes. Diabetes Care. 1995 Oct;18(10):1383-94.
17. Larsson J, Agardh CD, Apelqvist J, Stenstrom A. Long-term prognosis after healed amputation in patients with diabetes. Clin Orthop Relat Res. 1998 May(350):149-58.
18. Reiber GE, Boyko EJ, Smith DG. Lower extremity foot ulcers and amputations in diabetes. In: Harris MI, Cowie C, Stern MP, editors. Diabetes in America1995. p. 409-27.
19. Reiber GE, Boyko EJ, Smith DG. Lower extremity foot ulcers and amputations in diabetes. Diabetes in America 2nd ed. 1995:409-27.
20. Boulton AJ. The diabetic foot: from art to science. The 18th Camillo Golgi lecture. Diabetologia. 2004 Aug;47(8):1343-53.
21. Pecoraro RE, Reiber GE, Burgess EM. Pathways to diabetic limb amputation. Basis for prevention. Diabetes Care. 1990 May;13(5):513-21.
22. Knox RC, Dutch W, Blume P, Sumpio BE. Diabetic Foot Disease. Int J Angiol. 2000 Jan;9(1):1-6.
23. Mueller MJ, Diamond JE, Delitto A, Sinacore DR. Insensitivity, limited joint mobility, and plantar ulcers in patients with diabetes mellitus. Phys Ther. 1989 Jun;69(6):453-9; discussion 9-62.
24. American Diabetes Association. Clinical practice recommendations 1996. Diabetes Care 1996 Jan. 1996;19 (Suppl 1):S1-118.
25. Adler AI, Boyko EJ, Ahroni JH, Stensel V, Forsberg RC, Smith DG. Risk factors for diabetic peripheral sensory neuropathy. Results of the Seattle Prospective Diabetic Foot Study. Diabetes Care 1997 Jul. 1997;20(7):1162-7.
26. March JR, López-Quintana A, Guilleuma J. Diabetic foot management. In: Pharmacotherapy D, editor. Ros Die. Barcelona1998. p. 537-41.
27. Tomás P, Diabetic foot work group GEDAPS. Diabetic foot. Aten Primaria. 1996;18(10):533-4.
28. Gibbons GW. Lower extremity bypass in patients with diabetic foot ulcers. Surg Clin North Am. 2003 Jun;83(3):659-69.
29. Frykberg RG. An evidence-based approach to diabetic foot infections. Am J Surg. 2003 Nov 28;186(5A):44S-54S; discussion 61S-4S.
30. Serrano FJ, Martín A. Enfermedad arterial periférica: aspectos fisiopatológicos, clínicos y terapéuticos. Rev Esp Cardiol. 2007;60(9):969-82.
31. Kannel WB. Risk factors for atherosclerotic cardiovascular outcomes in different arterial territories. J Cardiovasc Risk. 1994 Dec;1(4):333-9.
32. Apelqvist J, Bakker K, van Houtum WH, Nabuurs-Franssen MH, Schaper NC. International consensus and practical guidelines on the management and the prevention of the diabetic

foot. International Working Group on the Diabetic Foot. Diabetes Metab Res Rev. 2000 Sep-Oct;16 Suppl 1:S84-92.

33. Consensus Development Conference on Diabetic Foot Wound Care: 7-8 April 1999, Boston, Massachusetts. American Diabetes Association. Diabetes Care. 1999 Aug;22(8):1354-60.
34. Geerlings SE, Hoepelman AI. Immune dysfunction in patients with diabetes mellitus (DM). FEMS Immunol Med Microbiol. 1999 Dec;26(3-4):259-65.
35. Caputo GM. The rational use of antimicrobial agents in diabetic foot infection. In: Boulton AJM, Connor H, Cavanagh PR, Wiley and Sons, editors. The Foot in Diabetes. 3rd ed. Chichester1994. p. 143-51.
36. Eneroth M, Larsson J, Apelqvist J. Deep foot infections in patients with diabetes and foot ulcer: an entity with different characteristics, treatments, and prognosis. J Diabetes Complications. 1999 Sep-Dec;13(5-6):254-63.
37. Frykberg RG, Zgonis T, Armstrong DG, Driver VR, Giurini JM, Kravitz SR, et al. Diabetic foot disorders. A clinical practice guideline (2006 revision). J Foot Ankle Surg. 2006 Sep-Oct;45(5 Suppl):S1-66.
38. Caputo GM, Cavanagh PR, Ulbrecht JS, Gibbons GW, Karchmer AW. Assessment and management of foot disease in patients with diabetes. N Engl J Med. 1994 Sep 29;331(13):854-60.
39. Gibbons GW, Eliopoulus GM. Infection of the diabetic foot. In: Kozak GP, Campbell DR, Frykberg RG, Habershaw GM, Saunders WB, editors. Management of the Diabetic Foot Problems. Philadelphia1995. p. 121.
40. Karchmer AW, Gibbons GW. Foot infections in diabetes: evaluation and management. Curr Clin Top Infect Dis. 1994;14:1-22.
41. Eneroth M, Apelqvist J, Stenstrom A. Clinical characteristics and outcome in 223 diabetic patients with deep foot infections. Foot Ankle Int. 1997 Nov;18(11):716-22.
42. Fard AS, Esmaelzadeh M, Larijani B. Assessment and treatment of diabetic foot ulcer. Int J Clin Pract. 2007 Nov;61(11):1931-8.
43. Unger RF, Foster DW. Diabetes mellitus. In: Williams RH, Wilson JD, Foster DW, editors. Williams textbook of endocrinology. 8th ed. Philadelphia: WB Saundeers Company; 1992. p. 1255-333.
44. Edmonds ME, Blundell MP, Morris ME, Thomas EM, Cotton LT, Watkins PJ. Improved survival of the diabetic foot: the role of a specialized foot clinic. Q J Med. 1986 Aug;60(232):763-71.
45. Frykberg RG. Diabetic foot ulcers: pathogenesis and management. Am Fam Physician. 2002 Nov 1;66(9):1655-62.

46. Aksoy DY, Gurlek A, Cetinkaya Y, Oznur A, Yazici M, Ozgur F, Aydingoz U, Gedik O: Change in the amputation profile in diabetic foot in a tertiary reference center: efficacy of team working. Exp Clin Endocrinol Diabetes 112:526-530, 2004.
47. Stanley S, Turner L: A collaborative care approach to complex diabetic foot ulceration. Br J Nurs 13:788-793, 2004.
48. Sandnes DK, Sobel M, Flum DR: Survival after lower-extremity amputation. J Am Coll Surg 199:394-402, 2004.
49. Davis BL, Kuznicki J, Praveen SS, Sferra JJ: Lower-extremity amputations in patients with diabetes: pre- and post-surgical decisions related to successful rehabilitation. Diabetes Metab Res Rev 20 Suppl 1:S45-S50, 2004.
50. Saar WE, Lee TH, Berlet GC: The economic burden of diabetic foot and ankle disorders. Foot Ankle Int 26:27-31, 2005.
51. Cruz CP, Eidt JF, Capps C, Kirtley L, Moursi MM: Major lower extremity amputations at a Veterans Affairs hospital. Am J Surg 186:449-454, 2003.
52. Andros G: Diagnostic and therapeutic arterial interventions in the ulcerated diabetic foot. Diabetes Metab Res Rev 20 Suppl 1:S29-S33, 2004.
53. Margolis DJ, Len-Taylor L, Hoffstad O, Berlin JA: Healing diabetic neuropathic foot ulcers: are we getting better? Diabet Med 22:172-176, 2005.
54. Chantelau E, Tanudjaja T, Altenhofer F, Ersanli Z, Lacigova S, Metzger C: Antibiotic treatment for uncomplicated neuropathic forefoot ulcers in diabetes: a controlled trial. Diabet Med 13:156-159, 1996.
55. Lipsky BA, Itani K, Norden C: Treating foot infections in diabetic patients: a randomized, multicenter, open-label trial of linezolid versus ampicillin-sulbactam/amoxicillin-clavulanate. Clin Infect Dis 38:17-24, 2004.
56. Lew DP, Waldvogel FA: Osteomyelitis. N Engl J Med 336:999-1007, 1997.
57. Senneville E, Yazdanpanah Y, Cazaubiel M, Cordonnier M, Valette M, Beltrand E, Khazarjian A, Maulin L, Alfandari S, Caillaux M, Dubreuil L, Mouton Y: Rifampicin-ofloxacin oral regimen for the treatment of mild to moderate diabetic foot osteomyelitis. J Antimicrob Chemother 48:927-930, 2001.
58. Venkatesan P, Lawn S, Macfarlane RM, Fletcher EM, Finch RG, Jeffcoate WJ: Conservative management of osteomyelitis in the feet of diabetic patients. Diabet Med 14:487-490, 1997.
59. Boulton AJ, Armstrong DG: Whither progress in the diabetic foot--clinical and research? Int J Low Extrem Wounds 3:182-183, 2004.
60. Lipsky BA, Berendt AR, Deery HG, Embil JM, Joseph WS, Karchmer AW, Lefrock JL, Lew DP, Mader JT, Norden C, Tan JS: Diagnosis and treatment of diabetic foot infections. Clin Infect Dis 39:885-910, 2004.

61. Macfarlane RM, Jeffcoate WJ: Factors contributing to the presentation of diabetic foot ulcers. Diabet Med 14:867-870, 1997.
62. Leymarie F, Richard JL, Malgrange D: Factors associated with diabetic patients at high risk for foot ulceration. Diabetes Metab 31:603-605, 2005.
63. Boulton AJ, Kirsner RS, Vileikyte L: Clinical practice. Neuropathic diabetic foot ulcers. N Engl J Med 351:48-55, 2004.
64. Chantelau E, Kushner T, Spraul M: How effective is cushioned therapeutic footwear in protecting diabetic feet? A clinical study. Diabet Med 7:355-359, 1990.
65. Uccioli L, Faglia E, Monticone G, Favales F, Durola L, Aldeghi A, Quarantiello A, Calia P, Menzinger G: Manufactured shoes in the prevention of diabetic foot ulcers. Diabetes Care 18:1376-1378, 1995.
66. Valk GD, Kriegsman DM, Assendelft WJ: Patient education for preventing diabetic foot ulceration. A systematic review. Endocrinol Metab Clin North Am 31:633-658, 2002.
67. Calle-Pascual AL, Duran A, Benedi A, Calvo Mi, Charro A, Diaz JA, Calle JR, Gil E, Maranes JP, Cabezas-Cerrato J: A preventative foot care programme for people with diabetes with different stages of neuropathy. Diabetes Res Clin Pract 57:111-117, 2002.
68. Dalla PL, Faglia E, Caminiti M, Clerici G, Ninkovic S, Deanesi V: Ulcer recurrence following first ray amputation in diabetic patients: a cohort prospective study. Diabetes Care 26:1874-1878, 2003.
69. Thompson P, Langemo D, Hunter S, Hanson D, Anderson J: Offloading diabetic foot ulcers. Adv Skin Wound Care 19:15-6, 19, 2006.
70. Van SC, Ulbrecht JS, Becker MB, Cavanagh PR: Design criteria for rigid rocker shoes. Foot Ankle Int 21:833-844, 2000.
71. Edmonds ME, Blundell MP, Morris ME, Thomas EM, Cotton LT, Watkins PJ: Improved survival of the diabetic foot: the role of a specialized foot clinic. Q J Med 60:763-771, 1986.
72. Larsson J, Apelqvist J, Agardh CD, Stenstrom A: Decreasing incidence of major amputation in diabetic patients: a consequence of a multidisciplinary foot care team approach? Diabet Med 12:770-776, 1995.
73. Frykberg RG: The team approach in diabetic foot management. Adv Wound Care 11:71-77, 1998.
74. Jeffcoate WJ, Lipsky BA, Berendt AR, Cavanagh PR, Bus SA, Peters EJ, et Al. Unresolved issues in the management of ulcers of the foot in diabetes. Diabet Med. 2008 Dec;25(12):1380-9.
75. Sinacore DR. Healing times of pedal ulcers in diabetic immunosuppressed patients after transplantation. Arch Phys Med Rehabil. 1999 Aug;80(8):935-40.

76. Ghanassia E, Villon L, Thuan Dit Dieudonne JF, Boegner C, Avignon A, Sultan A. Long-term outcome and disability of diabetic patients hospitalized for diabetic foot ulcers: a 6.5-year follow-up study. Diabetes Care. 2008 Jul;31(7):1288-92.
77. Preventive foot care in people with diabetes. Diabetes Care. 2000 Jan;23 Suppl 1:S55-6.
78. Boulton AJ, Jude EB. Therapeutic footwear in diabetes: the good, the bad, and the ugly? Diabetes Care. 2004;27(7):1832-3.
79. Arts ML, Waaijman R, de Haart M, Keukenkamp R, Nollet F, Bus SA. Offloading effect of therapeutic footwear in patients with diabetic neuropathy at high risk for plantar foot ulceration. Diabet Med. 2012;29(12):1534-41.
80. Fernandez ML, Lozano RM, Diaz MI, Jurado MA, Hernandez DM, Montesinos JV. How effective is orthotic treatment in patients with recurrent diabetic foot ulcers? J Am Podiatr Med Assoc. 2013;103(4):281-90.
81. Bus SA. Priorities in offloading the diabetic foot. Diabetes Metab Res Rev. 2012;28(Suppl 1):54-9.
82. Bus SA, Ulbrechtb JS, Cavanagh PR. Pressure relief and load redistribution by custom-made insoles in diabetic patients with neuropathy and foot deformity . Clinical biomechanics 2004.volum 19, issue 6, pg 629-638.
83. Wu WL, Rosenbaum D, Su FC. The effects of rocker sole and SACH heel on kinematics in gait. Med Eng Phys. 2004;26(8):639-46.
84. Myers KA1, Long JT, Klein JP, Wertsch JJ, Janisse D, Harris GF. Biomechanical implications of the negative heel rocker sole shoe: gait kinematics and kinetics. Gait Posture. 2006;24(3):323-30.
85. Brown D, Wertsch JJ, Harris GF, Klein J, Janisse D. Effect of rocker soles on plantar pressures. Arch Phys Med Rehabil. 2004;85(1):81-6.
86. Praet SF, Louwerens JW. The influence of shoe design on plantar pressures in neuropathic feet. Diabetes Care. 2003;26(2):441-5.
87. Healy A, Naemi R, Chockalingam N. The effectiveness of footwear as an intervention to prevent or to reduce biomechanical risk factors associated with diabetic foot ulceration: a systematic review. J Diabetes Complications. 2013;27(4):391-400.
88. Mayfield JA, Sugarman JR. The use of the Semmes-Weinstein monofilament and other threshold tests for preventing foot ulceration and amputation in persons with diabetes. J Fam Pract. 2000 Nov; 49(11uppl):S17-29.
89. Reiber GE. The epidemiology of diabetic foot problems. Diabet Med. 1996;13(suppl 1):S6-S11.
90. Prompers L, Huijberts M, Schaper N, Apelqvist J, Bakker K, Edmonds M, et al. Resource utilisation and costs associated with the treatment of diabetic foot ulcers. Prospective data from the Eurodiale Study. Diabetologia. 2008;51(10):1826-34.

91. Yong R, Karas TJ, Smith KD, Petrov O. The durability of the Semmes-Weinstein 5.07 monofilament. J Foot Ankle Surg. 2000 Jan-Feb; 39(1):34-8.
92. Armstrong DG, Lavery LA, Vela SA, Quebedeaux TL, Fleischli JG. Choosing a practical screening instrument to identify patients at risk for diabetic foot ulceration. Arch Intern Med. 1998 Feb 9; 158(3):289-92.
93. Kalani M, Brismar K, Fargrell B, Östergren J, Jörneskog G. “Transcutaneous oxygen tensión and toe blood pressure as predictors for outcome of diabetic foot ulcers”. Diabetes Care 22:147-151, 1999.
94. Michaud TC: “Abnormal motion during the gait cycle”, in Foot Orthoses and other conservative foot care. Ed by Thomas C. Michaud, p 57-173, Williams & Wilkins. Newton, Massachusetts, 1991.
95. Root ML, Orien WP, Weed JH, Hugues RJ. Exploración Biomecánica del Pie. VOL 1. Ortocen SA. Madrid. 1991.
96. Molines-Barroso RJ, Lázaro-Martínez JL, Aragón-Sánchez FJ, Álvaro-Afonso FJ, García-Morales E, García-Álvarez Y. Forefoot ulcer risk is associated with foot type in patients with diabetes and neuropathy. Diabetes Res Clin Pract. 2016 Jan 14. pii: S0168-8227(16)00057-7. doi: 10.1016/j.diabres.2016.01.008.
97. Bacarin TA, Sacco IC, Hennig EM. Plantar pressure distribution patterns during gait in diabetic neuropathy patients with a history of foot ulcers. Clinics (Sao Paulo). 2009;64(2):113-20.
98. Craig CL, Marshall AL, Sjöström M, Bauman AE, Stand ML, Ainsworth BE, et al. International physical activity ques- tionnaire: 12-country reliability and validity. Med Sci Sports Exerc 2003;35:1381e95.
99. Bakker K, Apelqvist J, Schaper NC. Practical guidelines on the management and prevention of the diabetic foot 2011. Diabetes Metab Res Rev 2012; 28(Suppl 1): 225–231.
