# Supplementary material for: Clinical efficacy of therapeutic footwear with a rigid rocker sole in the prevention of recurrence in patients with diabetes mellitus and diabetic polineuropathy: A randomized clinical trial
Source: PLoS One. 2019 Jul 11;14(7):e0219537. doi: 10.1371/journal.pone.0219537 (PMC6623964; doi:10.1371/journal.pone.0219537)
Supplement: S4 File — Long Spanish version. (PDF) [file pone.0219537.s004.pdf]

Eficacia clínica del calzado terapéutico con balancín para la prevención de la re-ulceración en los pacientes con Diabetes mellitus y polineuropatía diabética: ensayo clínico prospectivo y aleatorizado.

Investigador principal: Prof. Dr. José Luis Lázaro Martínez

CLÍNICA UNIVERSITARIA DE PODOLOGÍA

UNIVERSIDAD COMPLUTENSE DE MADRID

Versión protocolo 1: Septiembre 2016

Índice:

Resumen…………………………………………………………….. *3*

Introducción………………………………………………………….. *5*

Hipótesis…………………………………………………………….... *17*

Objetivos……………………………………………………………… *17*

Metodología y Plan de Trabajo…………………………………. *18*

Bibliografía………………………………………………………….. *38*

Anexos………………………………………………………………… *45*

Resumen:

El proceso fisiopatológico del síndrome del pie diabético comprende una triada etiológica, neuropática, vascular e infecciosa. La polineuropatía periférica es el mayor factor de riesgo de ulceración manifestándose con afectación sensitiva, motora y autonómica. La neuropatía motora se ha asociado con el desarrollo de deformidades en el pie que generan un aumento de las presiones plantares, lo cual, unido a la falta de sensibilidad como consecuencia de la afectación sensitiva en pacientes diabéticos va a aumenta el riesgo de sufrir úlceras plantares.

Aunque el uso del calzado terapéutico para la prevención de úlceras está bien aceptado en la práctica clínica, todavía la evidencia científica no es concluyente. Esto puede ser debido probablemente a que no existen guías basadas en la evidencia ni protocolos estandarizados para la prescripción de calzado diabético. Como consecuencia existe una amplia diversidad de métodos, materiales y diseños usados en la prescripción de calzados, lo cual empeora aun más la situación. Por esto, la información actual sobre la eficacia del calzado es claramente deficiente, aumentando la posibilidad de usar el calzado de forma inadecuada. El análisis cuantitativo mediante dispositivos de medición de presiones nos permitirá investigar que calzado terapéutico ofrece los mejores resultados en la reducción de presiones plantares y el uso prolongado de estos dispositivos en la práctica clínica podría reducir la tasa de recurrencia de lesiones de pie diabético en estos pacientes.

Objetivo:

Analizar la eficacia del uso de la suela rígida en balancín en la reducción de la tasa de recurrencia de úlceras plantares en los pacientes con pie diabético.

Material y Método:

Ensayo clínico prospectivo y aleatorizado en el que se pretenden analizar 138 pacientes diabéticos con antecedentes de úlceras plantares. Los pacientes serán aleatorizados mediante una tabla de aleatorización en dos grupos: calzado terapéutico estándar (grupo control) y calzado terapéutico estándar con balancín rígido (grupo experimental). Se pretenden estudiar los factores de riesgo generales y biomecánicos que aumentan el riesgo de lesión. Los pacientes serán seguidos durante un periodo de 6 meses, donde se evaluará la tasa de recurrencia de úlceras plantares

Los resultados del presente trabajo permitirán definir las estrategias preventivas, diagnosticas, terapéuticas y ortopédicas que ayuden a reducir el número de reulceraciones y de amputaciones asociadas al pie diabético en base al patrón biomecánico de cada paciente.

Palabras clave:

Pie Diabético, Alteraciones Biomecánicas, Úlcera de Pie Diabético, Limitación Movilidad Articular, Calzado terapéutico, Calzado con balancín.

Introducción:

Diabetes *mellitus*

Definición de Diabetes *mellitus*

La Diabetes *mellitus* (DM) es una alteración metabólica caracterizada por la presencia de hiperglucemia crónica que se acompaña, en mayor o menor medida, de alteraciones en el metabolismo de los hidratos de carbono, de las proteínas y de los lípidos. El origen y la etiología de la DM pueden ser muy diversos, pero conllevan inexorablemente la existencia de alteraciones en la secreción de insulina, de la sensibilidad a la acción de la hormona, o de ambas en algún momento de su historia natural. En aquellos casos en que los síntomas son floridos, persistentes y las cifras de glucemia suficientemente elevadas, el diagnóstico es obvio en la mayoría de ocasiones. Pero no se debe olvidar que, en muchos casos, el diagnóstico se realiza a sujetos asintomáticos y a través de una exploración analítica de rutina (1).

Prevalencia e incidencia de Diabetes *mellitus*

La prevalencia de la DM, sus complicaciones específicas y la presencia de otras entidades que suelen acompañarla, hacen que la enfermedad sea un problema socio sanitario en la actualidad, constituyendo una de las principales causas de muerte sobre todo en los países desarrollados y una de las causas de insuficiencia renal, ceguera y amputación no traumática (2).

La prevalencia mundial de la DM en el año 2003 se estimó en 194 millones (3), pero para el año 2030, está previsto que esta cifra aumente a 366 millones, debido al aumento de la esperanza de vida en la población y al cambio en los hábitos nutricionales (4).

En los EE.UU., la incidencia de la DM es superior a los 1,5 millones de casos nuevos al año, con una prevalencia global de 17,7 millones de personas, o el 7% de la población del país. Se estima que actualmente 14,6 millones de personas son diagnosticadas, mientras que otras 6,2 millones de personas que padecen DM, no se diagnostican, lo que representa un aumento en seis veces el número de personas con DM en las próximas cuatro décadas (3).

En Europa, la prevalencia de la DM es un 3% más que el Norte de América, cifrándose un total de 33,3 millones de diabéticos repartidos en los países que la conforman y por tanto, estableciéndose como la región del mundo con mayor prevalencia de esta enfermedad (3).

En España, la prevalencia media total, está en un 6,05% y se estima que a partir de los 70 años la prevalencia puede llegar al 25% (5). Se cree que estas cifras de prevalencia en España son superiores a las de otros países, teniendo en cuenta que se calcula que alrededor del 50% queda sin diagnosticar (6).

Análisis de costes de la Diabetes *mellitus*

El elevado coste del tratamiento de la DM tipo 2 y sus complicaciones, sugiere la posibilidad de que el buen control de la enfermedad pueda no sólo mejorar la supervivencia y la calidad de vida, sino reducir los costes asociados con las complicaciones crónicas, especialmente entre los jóvenes y personas con problemas de obesidad.

Se calcula que la población con diabetes puede consumir entre un 4 y un 14% del gasto sanitario global en los países occidentales y de forma individual un paciente con diabetes puede consumir entre 2 y 6 veces más recursos directos, que los individuos de similares edad y sexo con otras enfermedades crónicas (7).

En España se han realizado varios estudios de costes donde se reflejaron los costes de la DM y sus complicaciones. En el 2004, Ballesta y colaboradores estimaron un coste anual asociado a la diabetes de 4.278€/paciente/año, siendo la partida de costes directos superior (2.540€/paciente/año) que los costes indirectos (1.774€/paciente/año). Además estos costes fueron significativamente superiores en pacientes varones, obesos, en tratamiento con insulina y que requirieron hospitalización (8). Por ello es importante señalar que el control exhaustivo de la DM reduce los costes asociados a las complicaciones (9).

Complicaciones de la Diabetes *mellitus*

Sin embargo, el desarrollo de complicaciones crónicas en pacientes con DM es cada vez más frecuente, induciendo tasas altas de morbilidad e incluso mortalidad. La etiología de estas complicaciones a corto, medio y largo plazo es microvascular, macrovascular y metabólica.

Actualmente una de las complicaciones a largo plazo más frecuente y grave es el conjunto de las angiopatías derivadas de la ateroesclerosis, que pueden ocasionar claudicación intermitente, gangrena, enfermedad coronaria e ictus, y hemorragias internas. La alteraciones a nivel microvascular afectan al corazón (miocardiopatías), ojos (retinopatías, que pueden conducir a la ceguera), riñones (nefropatías) y sistema nervioso (neuropatías). Las causas de mortalidad más frecuentes en la población son las enfermedades del corazón con un 70%, seguidas de la propia DM, el cáncer y la neumonía (10).

La complicación tardía más incapacitante de la DM es el síndrome del "Pie Diabético", cuyo proceso etiopatogénico comprende un triada etiológica neuropática, vascular e infecciosa. Cerca del 15% de los diabéticos de varios años de evolución desarrollarán una úlcera en el pie a lo largo de su vida y en ocasiones puede llevar a la amputación del pie o de la pierna (15%-25%) (11). Tanto es así, que en España, la DM es la primera causa desencadenante de amputación no traumática, y en ocasiones representa en torno al 60%-80% de las amputaciones del miembro inferior. En el 85% de los casos, la amputación está siempre precedida de una úlcera en el pie (2).

Pie Diabético

Definición de Pie Diabético

La Organización Mundial de la Salud (OMS) define pie diabético como la infección, la ulceración y la destrucción de los tejidos profundos, asociadas con anormalidades neurológicas (pérdida de la sensibilidad al dolor) y vasculopatía periférica de diversa gravedad en las extremidades inferiores (12).

Prevalencia de Pie Diabético

La prevalencia de las úlceras de pie diabético se encuentra en un rango entre el 2 y el 10%, con una incidencia acumulada del PD, de entre el 0,5 y 3 % anual (13).

Se estima que el 50% de las amputaciones no traumáticas que se realizan en hospitales del primer mundo, son debidas al pie diabético (14), siendo la causa más frecuente de amputación no traumática de extremidad inferior en los Estados Unidos y en el continente europeo (2).

Si la úlcera del paciente llega a cicatrizar, se estima que tras 1, 3 y 5 años de seguimiento, el 34%, 61% y 70% de los pacientes cuyas úlceras cicatrizaron desarrollarán una nueva úlcera (15). Pero existe un dato aún más alarmante y es el hecho de que la supervivencia a los 3 años después de haber sufrido una amputación mayor es sólo del 50% y a los 5 años del 40% (16). Un estudio de Larsson et al., incrementa esta tasa de mortalidad a un 68 %, a los 5 años de haber sufrido una amputación mayor (17). Además, la incidencia de sufrir amputación en el miembro contralateral pasados de 2 a 5 años es del 50% (18).

La duración de la estancia media hospitalaria de un paciente con úlcera de pie diabético, es un 59% más larga que la de un paciente sin esta complicación (19).

Etiopatogenia de Pie Diabético

Los factores de riesgo identificados en diversos estudios para el padecimiento de lesión de pie diabético pueden ser locales o generales. Dentro de los locales se encuentran la neuropatía, la limitación de la movilidad articular, la presencia de deformidades y patrón de hiperqueratosis las presiones plantares anormales, los traumatismos previos y los antecedentes de úlcera y/o amputación (20). Por otro lado los factores de riesgo general o sistémico son el mal control glucémico, el tiempo de evolución de la DM, la enfermedad vascular periférica, la retinopatía, la nefropatía y edades del paciente por encima de 60 años (21).

Entre estos factores, son dos mecanismos principalmente, deformidad y neuropatía, los determinantes de que el paciente diabético desarrolle una úlcera en el pie (22). El primer mecanismo de la lesión se produce en prominencia ósea causando heridas en bordes laterales y dorsales principalmente del antepié y se asocia con el uso de zapatos inadecuados. El otro mecanismo común de ulceración moderada implica estrés repetitivo prolongado. Esto normalmente ocurre en la planta del pie en zonas de prominencias óseas a nivel de metatarsianos donde se ha producido una disminución o desplazamiento de la grasa plantar. Deformidades rígidas tales como el hallux valgus, hallux rígidus, dedo en garra, pie de Charcot o amputaciones previas limitan el rango de movimiento del tobillo (23).

Neuropatía diabética

En 1996, la Asociación Americana de Diabetes (ADA), definió la neuropatía diabética como: «aquella alteración de la función de los nervios periféricos que ocurre en pacientes con diabetes mellitus en ausencia de otras causas de neuropatía periférica». Posteriormente, en 1999, Boulton refrendó esta definición como: «la presencia de síntomas, signos o ambos de disfunción de nervios periféricos en personas con diabetes una vez que se descartan otras causas» (24). De esta forma, ambas definiciones coinciden en que la neuropatía diabética produce una alteración estructural y funcional de los nervios (de causa diabética), desarrollando una serie de síntomas y signos que determinarán su diagnóstico, diferente en todo momento a otro tipo de sintomatología producida por otros tipos de neuropatía como puede ser la producida por el consumo de alcohol.

La neuropatía diabética está presente en aproximadamente el 50% de los diabéticos con más de 15 años de evolución. Existe una relación directa entre la aparición de la neuropatía y un mal control metabólico de la diabetes, por lo que se pueden detectar signos incipientes de neuropatía en pacientes con pocos años de evolución, pero con un mal control metabólico (25).

Enfermedad vascular periférica

La enfermedad vascular periférica (EVP) favorece la aparición y complicación de las úlceras en el Pie Diabético. Se estima que la EVP está presente en el 8-13% de los diabéticos, incrementándose su prevalencia a medida que lo hacen los años de padecimiento de la enfermedad. Suele presentarse como manifestación clínica o subclínica, en todos los diabéticos de más de 25 años de evolución (26, 27).

Rara vez conduce directamente a la ulceración del pie, sin embargo, una vez desarrollada la úlcera, dificultará el tiempo de cicatrización, la llegada de antibióticos a la zona de infección y aumentará el riesgo de amputación (28). Por tanto, la perfusión arterial es un componente vital para la cicatrización y un riesgo subyacente de amputación (28), siendo fundamental una evaluación y derivación precoces (29).

Está producida principalmente por arteriosclerosis, que produce una isquemia, donde en los casos más críticos, supone la amputación del miembro. Aunque el factor de riesgo de mayor relevancia es el tabaquismo; sin embargo, la DM constituye en sí misma un importante factor de riesgo aterosclerótico; hasta tal punto, que por cada aumento del 1%

de la hemoglobina glicosilada se produce un incremento del 25% en el riesgo de padecer EVP. Cuanto mayor sea la afectación vascular menos posibilidades de cicatrización existen y, en los casos más avanzados, será necesaria la opción quirúrgica de revascularización para evitar una complicación mayor como la amputación (30). Si el paciente no fuera susceptible de cirugía de revascularización distal, el pronóstico es siempre malo alcanzando cifras de hasta un 40 % de posibilidad de desarrollar úlceras o gangrena distal, frente al 9% en pacientes no diabéticos (31). Por ello, un diagnóstico precoz de los pacientes diabéticos con isquemia y una terapia agresiva, van a ser cruciales en la salvación del miembro inferior (32) teniendo en cuenta que la localización anatómica de la oclusión, suele producir afectación infrainguinal.

La enfermedad vascular en el paciente diabético se ha descrito en una doble vertiente macroangiopática y microangiopática.

Infección

La infección en el Pie diabético es casi diez veces más frecuente que en los pacientes no diabéticos, con un riesgo alto de producirse osteomielitis y de precipitar una amputación, puesto que entre el 14%-20% de estas infecciones graves conducen a una amputación, siendo a su vez la úlcera precursora de más del 85% de la amputaciones MMII (21).

El factor más importante que predispone a la infección en el pie de los pacientes diabéticos es la pérdida de la integridad de la barrera cutánea coincidiendo casi siempre con infecciones polimicrobianas (33). El mal control metabólico en los pacientes diabéticos con hiperglucemias mantenidas produce alteraciones en la función leucocitaria, anormalidades en la actividad bactericida y deficiencias en la actividad celular, lo que aumenta el riesgo de infección (34). Esto supone un retraso en la llegada del antibiótico al lugar de la infección, pudiéndose propagar esta infección rápidamente con daños irreversibles en los tejidos (35), llevando a la amputación o a la muerte en un estadío final. Incluso aun teniendo una perfusión arterial adecuada, la neuropatía ralentizaría la detección por parte del paciente del empeoramiento de la lesión (36).

Podemos encontrarnos diferentes presentaciones de infecciones en el pie diabético. Por ello es necesario clasificarlas en función de la gravedad (37) para determinar si es amenazante o no de la extremidad (38):

- Las infecciones no amenazantes de la extremidad: menos de 2 cm. de celulitis desde la úlcera o puerta de entrada (39):

- Úlceras superficiales
- No existe evidencia de isquemia ni infección profunda
- Sin signos ni síntomas
- Manejo en consultas externas

- Las infecciones amenazantes de la extremidad: celulitis mayor de 2 cm (40):
  - Úlceras profundas
  - Isquemia
  - Fiebre, edema, linfangitis, leucocitosis, hiperglucemia. Pueden no presentar sintomatología en un 50% de los casos (41).
  - Manejo urgente en Atención especializada.

En la actualidad, cerca de un 20% de los ingresos hospitalarios de pacientes diabéticos, son causados por problemas resultantes del pie (42). Así, las estancias en el hospital son aproximadamente un 60% más largas en pacientes con úlceras, en comparación con pacientes sin úlceras (43). La literatura médica recoge un índice de amputación en las partes más distales de los miembros inferiores en pacientes diabéticos de 17-40 veces superior que en pacientes no diagnosticados de DM.

La naturaleza multifactorial de la úlcera del pie diabético se ha estudiado en numerosos estudios observacionales (18), determinándose cómo factores etiopatogénicos primarios la neuropatía y enfermedad vascular periférica, desarrollando principalmente dos tipos de lesiones: la úlcera neuropática y la úlcera isquémica. Otros factores de riesgo asociados a los primarios como la limitación de la movilidad articular, las deformidades del pie, la alteración de las presiones plantares, los antecedentes previos de ulceración o amputación, la disminución de la agudeza visual y la infección (20) complicarán el tratamiento de las úlceras en el pie diabético. Debido a que la neuropatía es el principal factor etiológico que produce ulceras en el pie diabético (34), el 45% a 60% de las lesiones en el pie diabético son neuropáticas. Únicamente el 10-15% son úlceras isquémicas puras, mientras que hasta un 20-30% son neuroisquémicas (44).

Por tanto, la evaluación inicial de la úlcera del pie diabético debe ser amplia y sistemática para determinar los parámetros que podrían haber dado lugar a su aparición y que establecerán una u otra clínica, así como para determinar la presencia de factores que pueden afectar a la cicatrización de la herida (45). La posibilidad de encontrarnos diferentes lesiones en el pie diabético obliga a que éstas sean clasificadas para poder establecer una identificación etiológica y un protocolo de actuación para el tratamiento. De esta forma facilitamos el tratamiento y la predicción de los resultados esperados.

Actitud terapéutica

En los pacientes que reciben tratamiento adecuado en el momento oportuno, y con la actuación basada en la evidencia, de un grupo multidisciplinar (46, 47) compuesto al menos por: diabetólogos, cirujanos, podólogos, enfermeras especializadas, expertos en calzado, fisioterapeutas y protésicos, disminuye de una forma muy significativa la morbimortalidad (48, 49) y la mejora de su condición psíquica y física es mucho mayor que en los que no reciben este tratamiento; además el coste económico y social se reduce de una forma drástica (50, 51).

Cualquier úlcera debe evolucionar en las primeras 2 semanas; en caso contrario debe ser estudiada en una unidad de pie de diabético (52); ya que si no, muchos de estos pacientes con úlceras de pie de diabético serían tratados de forma inadecuada (53); lo cual prolonga la necesidad de cuidados y, en numerosas ocasiones, se llegaría a la amputación aumentando significativamente el coste y las consecuencias en la calidad de vida del paciente.

La evidencia actual no aconseja el uso de antimicrobianos para el tratamiento de úlceras de pie de diabético sin infección clínica y sólo con fines profilácticos (54).

La hospitalización es necesaria cuando aparecen complicaciones graves, aunque a veces, algunos pacientes pueden requerir ingreso en infecciones menos graves con el fin de llevar a cabo su diagnóstico, o bien, cuando el paciente presenta una baja adherencia terapéutica. En los demás casos, el tratamiento de la infección debe realizarse de forma ambulatoria (55).

La curación de la osteomielitis es difícil por varias razones como son: la escasa concentración de antibiótico en el foco de infección y el bajo metabolismo de los microorganismos implicados que, a su vez, están inmersos en un biofilm impermeable a los antibióticos (56). Estas características han llevado a que tradicionalmente la osteomielitis se haya tratado fundamentalmente con cirugía, sobre todo la crónica. Sin embargo, últimamente han aparecido publicaciones en las que se obtienen buenos resultados en osteomielitis del pie del diabético, con tratamiento antimicrobiano y cirugía escasa o nula (57, 58).

No obstante, la descarga de la úlcera de pie de diabético es el principio fundamental del tratamiento. La utilización de una terapia de descarga temporal adecuada condiciona la eficacia de cualquier tratamiento y su omisión en el manejo de estas lesiones, garantiza un pobre resultado (59). De la misma forma, terapias de descarga definitivas como las ortesis plantares ayudan a prevenir reulceraciones y por tanto, a reducir tasas de recurrencia de lesiones en estos pacientes.

Prevención

La educación del paciente resulta crucial en la prevención primaria de la úlcera del pie de diabético. El paciente debe aprender a controlar su glucemia, usar un calzado adecuado, evitar traumatismos, identificar y vigilar los posibles signos previos a la lesión y programar el control periódico por el podólogo. Estos cuidados deben de extremarse cuando el paciente sufre una neuropatía grave, deformidades del pie y enfermedad vascular periférica grave (60, 61).

Los factores de riesgo de la úlcera en el pie de diabético incluyen: la retinopatía, pues tienen mayor riesgo de tropezar y golpearse; el estatus psicosocial bajo, por la falta de conciencia del problema y el escaso acceso a la información y los servicios; la hiperqueratosis y niveles altos de la HbAc1, al traducir un escaso control de la diabetes. Es más, muchos de éstos son modificables por lo que es importante la prevención (62).

Todos los pacientes diabéticos deben someterse a una exploración podológica al menos una vez al año, mientras que aquellos que tienen factores de riesgo como la neuropatía, la historia de úlceras previas, enfermedad vascular, y deformidades en los pies, precisan exploraciones más frecuentes. El uso de calzado con la suficiente profundidad y anchura es crucial en la prevención (63).

La prevención de la recurrencia de la ulcera es uno de los objetivos del tratamiento de estos pacientes tras conseguir el cierre de ésta. Casi una de cada tres úlceras recurre al cabo del año y prácticamente todas a los 40 meses (64, 65).

Parece ser que los cambios en los hábitos saludables de los pacientes que han sufrido pie de diabético, tienen una temporalidad corta, y con demasiada frecuencia estos pacientes vuelven a reproducir conductas peligrosas que les llevan nuevamente a la reulceración. Por otro lado, la metodología, que en ocasiones se emplea en la educación de los mismos, tiene fallos evidentes y además su eficacia está pobremente evaluada. Los pacientes son educados de forma muy focalizada en el control metabólico y nutricional de la Diabetes, y se les informa poco de cómo evitar las complicaciones asociadas a ésta. Es cierto que muchas de ellas se previenen con un buen control glucémico, pero en el caso del Pie de Diabético se insiste poco en los tratamientos ortopodológicos definitivos como ortesis plantares personalizadas, el vestido y calzado del pie (66).

Existen evidencias que avalan los beneficios de un programa de prevención y educación en el pie de diabético. Un estudio publicado en nuestro país (67) demostró que los pacientes diabéticos sometidos a un plan preventivo, tienen tasas de ulceración más bajas que aquellos que no reciben educación. Sin embargo, en el mismo estudio los pacientes diabéticos de alto riesgo tenían una tasa de reulceración del 35.8 %. Dalla Paola (68) ha publicado una tasa de reulceración del 16.8% al año de producirse una amputación del primer radio, lo que nos orienta acerca del altísimo nivel de reulceración que sufren estos pacientes, pudiendo multiplicarse por 5 esta tasa a los tres años.

La prevención de la reulceración debe basarse en la aplicación inmediata de un tratamiento ortopodológico definitivo junto a un calzado adecuado, que compense las alteraciones biomecánicas del paciente y redistribuya, de forma segura, las presiones recibidas en el pie. Se trata en definitiva, de controlar los factores intrínsecos de cada paciente, para evitar los puntos de presión que pueden desarrollar hiperqueratosis y después úlceras (69, 70).

Las experiencias positivas de la inclusión de los servicios de Podología en el equipo de atención del pie de diabético, han sido ampliamente demostradas por autores de reconocido prestigio en este campo. Edmonds (71) demostró que la inclusión de un podólogo, en el equipo multidisciplinar de atención del pie de diabético, consiguió elevar la tasa de curación de úlceras en un 86% para las úlceras neuropáticas y en un 76% para las úlceras isquémicas.

En el mismo estudio se aporta una tasa de reducción de la amputación en un 58% durante el primer año de inclusión del servicio de podología. Larsson (72) en Suecia, nos muestra una reducción en la tasa de amputación del 75% con la creación de un equipo de atención integral del pie, con servicio de podología especializado.

Frykberg publicó que el éxito en el tratamiento de estos pacientes se basa en la creación de Unidades Multidisciplinares de Pie de Diabético (73).

Prevención de la reulceración

Se ha demostrado que el mejor abordaje de la problemática del pie diabético debe girar en torno a la prevención. Sin embargo uno de los aspectos pendientes de resolver aún es el alto índice de reulceración que sufren estos pacientes (74).

En los últimos años se han publicado varios estudios que analizan las tasas de reulceración de los pacientes con pie diabético. El primer estudio publicado por Sinacore (75) en 1999 describe una tasa del 32% en un seguimiento a 18 meses. De los estudios publicados el de mayor duración en el seguimiento es el publicado por Ghanassia y Colbs (76) en 2008 y publica una tasa de reulceración del 40,6% en 6 años y medio de seguimiento. En el año 2000 la Asociación Americana de Diabetes (77) publicó una tasa de reulceración del 50% en un seguimiento a 2 años.

Todos estos estudios nos hablan de tasas de reulceración en valores absolutos, lo que significa que probablemente esta tasa sería superior si se hubiera hecho una determinación estadística mediante un análisis de Kaplan-Meier o una Regresión de Cox. Es decir que probablemente estaríamos hablando de tasas de reulceración en torno al 60%-70% en períodos de seguimiento medios de 3 años.

El calzado mal adaptado es considerado el principal causante de ulceración en individuos con DM y pie de riesgo.(78) Es por este motivo, que el calzado debe guardar una serie de características que lejos de generar nuevas lesiones, puedan proteger el pie de tal modo que la incidencia de la ulceración sea menor. El zapato extra-ancho es la principal característica del calzado que se prescribe en todos los pacientes con pies insensibles, con el objetivo de acomodar las deformidades digitales dentro de la pala del zapato, disminuyendo la incidencia de nuevas lesiones. (5,79,80)

El uso del calzado terapéutico para la prevención de úlceras es bien aceptado en la práctica clínica, aunque la evidencia todavía no resulta concluyente; esto es probablemente debido a que no existen guías basadas en la evidencia y protocolos estandarizados para la prescripción de calzado diabético. Se han publicado algunos consensos de protocolos pero su efectividad aun permanece desconocida. Como consecuencia existe una amplia diversidad de métodos, materiales y diseños usados en la prescripción de calzados, lo cual empeora aun más la situación. Por esto, la información sobre la eficacia del calzado es claramente deficiente, lo cual aumenta la posibilidad de usar el calzado de forma inadecuada. (81)

Otro problema es que la evaluación del calzado terapéutico depende de la experiencia y la destreza del equipo de pie diabético y de un enfoque ensayo-error. Un enfoque más cuantitativo puede mejorar el diseño de la descarga: como el uso de dispositivos de medición de presiones, análisis computerizados y biomecánicos para el diseño de ortesis individualizadas pueden reducir un 30% los picos de presión en comparación con los dispositivos manufacturados. Estos planteamientos son novedosos y se comienzan a integrar la practica y actualmente su eficacia clínica está siendo evaluada. (81)

Es por tanto necesario disminuir la presión y para ello hay que diseñar y desarrollar un cuidado preventivo, que incluya un tratamiento ortopodológico consistente en plantillas que consigan reducir y redistribuir las presiones y la utilización de un calzado terapéutico, que facilite la dinámica a dicha redistribución (82).

La suela en balancín es una de las modificaciones externas del calzado más comúnmente prescrita en la población general para aliviar dolores en algunas patologías (artritis, fracturas, dolor,…), debido a que dicha suela sustituye el movimiento de la articulación del tobillo durante la marcha. Los cambios cinéticos y cinemáticos sobre el pie que genera la suela en balancín ayudan a la fase de despegue del pie, simulando la dorsiflexión del antepié y haciendo por tanto más confortable la marcha. (83, 84)

La suela en balancín es también prescrita de manera habitual entre los pacientes diabéticos con neuropatía periférica, con el objetivo de reducir las presiones en las áreas de antepié con elevado riesgo de ulceración, especialmente los metatarsianos.(85) De hecho se ha mostrado como la medida más efectiva para reducir las presiones en el antepié de los pacientes neuropáticos.(86)

Sin embargo, estos estudios (83-86) son de tipo observacional y extraen sus conclusiones de los resultados de la evaluación de los efectos en la reducción de las presiones plantares mediante estudios de laboratorio,(87) no existiendo evidencia científica que apoye estas intervenciones en la práctica clínica. (83)

Hasta la actualidad no se han encontrado estudios de diseño longitudinal que examinen la efectividad del calzado terapéutico con suela rígida en balancín en la prevención de úlceras. (87)

Hipótesis:

El uso de la suela rígida en balancín, reduce la tasa de recurrencia de úlceras plantares en el pie de los pacientes diabéticos con neuropatía periférica.

Objetivos:

Objetivo primario:

- Analizar la eficacia del uso de la suela rígida en balancín en la reducción de la tasa de recurrencia de úlceras plantares en los pacientes con pie diabético.

Objetivos secundarios:

- Identificar el rango límite de presiones plantares que se asocia con la recurrencia de úlceras plantares.
- Evaluar la efectividad en términos de cambios cinéticos del calzado con suela rígida en balancín.
- Valorar el tiempo de uso de los tratamientos preventivos por parte de los pacientes diabéticos, así como su influencia en el riesgo de reulceración.
- Identificar los factores de riesgo generales que se asocian con un mayor riesgo de reulceración.
- Identificar las alteraciones biomecánicas que se asocian con un mayor riesgo de reulceración.

Metodología y plan de trabajo:

Tipo de Estudio:

Ensayo clínico aleatorizado en el que se pretende investigar la eficacia de la suela rígida en balancín en la prevención de la reulceración de los pacientes diabéticos neuropáticos con antecedentes de úlceras plantares.

Población Diana:

Se pretenden incluir en el presente estudio 138 pacientes diabéticos que acudan a la Unidad de Pie Diabético de la Clínica Universitaria de Podología de la Universidad Complutense de Madrid, que cumplan los siguientes criterios:

Criterios de inclusión:

- Pacientes diabéticos Tipo 1 ó 2 independientemente del tratamiento farmacológico que reciban.
- Pacientes de ambos sexos mayores de edad.
- Pacientes con presencia de neuropatía periférica.
- Pacientes que no presenten heridas o úlceras en el momento de la exploración
- Pacientes con antecedentes de úlceras plantares en el pie
- Pacientes con o sin amputaciones menores anteriores al momento de inclusión el presente estudio.
- Pacientes que no necesiten mecanismos de ayuda para la marcha como bastones, muletas, férulas o cualquier otro dispositivo que interfiera en el desarrollo autónomo del paso.

Criterios de exclusión:

- Pacientes con amputación mayor infra o supracondílea.
- Pacientes con enfermedades de etiología reumática, que afecten al pie.
- Pacientes con neuropatía periférica de etiología diferente a la Diabetes *mellitus.*
- Pacientes que presenten criterios de isquemia crítica, descrito por la TACSII (88).

Cálculo del tamaño muestral.

Considerando que el 15% de la población diabética sufrirá una úlcera a lo largo de su vida (89) y que se ha observado que el 48,2% de las úlceras son de localización plantar, (90) se estima un tamaño muestral de 138 pacientes, con una precisión del 5% y un nivel de significación del 95%, asumiendo un 10 % de pacientes no evaluables.

Método de asignación a los grupos de estudio

Aleatorización mediante tabla y los que vayan al grupo experimental, implicará la aplicación del tratamiento mediante un modelo de calzado con suela rígida en balancín, mientras que al grupo control se le suministrará un modelo de calzado con suela semirrígida. Ver anexo 1 con los modelos de calzado para hombre y mujer. (83,85,86)
Todos los pacientes serán sometidos a un screening vascular y neurológico según los protocolos de diagnóstico de estas complicaciones definidos en las guías internacionales.

Exploración neurológica

Se definirán como pacientes neuropáticos aquellos que presente las siguientes características:

- Insensibilidad a más de 4 puntos en la exploración mediante el monofilamento de Semmes Weinstein 5.07 10 g. de un total de 10 puntos explorados (88, 91).
- Insensibilidad al biotensiómetro a voltajes superiores a 25 voltios (>25 v) (92).

Exploración vascular

Se definirán como pacientes isquémicos aquellos que presente la combinación de al menos 2 de las circunstancias que se describen a continuación:

- Ausencia de latido en la palpación de pulsos distales tibial posterior y pedio.
- Índice tobillo brazo (ITB) menor de 0.9 (88).
- Presión transcutánea de oxígeno ($TcpO_2$) inferior a 20 mmHg (93).

Una vez realizado el cribaje neurológico y vascular cada paciente se asignará al grupo control o al grupo experimental para su posterior análisis comparativo en el trascurso del estudio.

Descripción de variables a analizar y métodos de recogida de la información

A todos los pacientes, tras la firma del consentimiento informado (ver anexo 2) se les recogerán los datos de filiación y los relativos al estado de su Diabetes y al nivel de complicaciones crónicas asociadas al padecimiento de la misma.

Se realizará una exploración biomecánica donde se recogerán los valores de movilidad articular medida en grados de la articulación tibioperonea-astragalina (ATPA), articulación subastragalina (ASA), 1ª articulación metatarso-falángica en carga y en descarga, índice de postura del pie (IPP) y patrón de hiperqueratosis (94, 95, 96).

Para valorar la ATPA trazamos la línea media del tercio distal y lateral de la pierna a explorar y otra línea en la zona plantar y lateral del calcáneo y manteniendo la articulación subastragalina neutra, flexionamos dorsalmente el pie y registramos los grados de dorsiflexión de la ATPA.

La valoración de la ASA se realiza trazando la bisección de la cara posterior del calcáneo a lo largo de los dos tercios proximales manteniendo la ASA en posición neutra y trazando también la línea media del tercio distal y posterior de la pierna evitando el Tendón de Aquiles. Partiendo de la posición neutra de la ASA, se coloca el pie en máxima supinación y con un brazo del goniómetro paralelo al bisector calcáneo y otro brazo paralelo a la bisección de la pierna, se mide el ángulo resultante. Se realiza la misma operación con la ASA en máxima pronación.

Para valorar el rango de la 1ª articulación metatarso-falángica en descarga, trazamos la línea media de la diáfisis del 1er metatarsiano, marcamos el punto medio de la cabeza del 1er meta, donde colocaremos el fulcro del goniómetro, y finalmente, trazamos la línea media de la diáfisis de la falange proximal del hallux.

Con el paciente en bipedestación pero en visión lateral mediremos también la flexión dorsal del hallux colocando un brazo del goniómetro paralelo al plano del suelo, el fulcro sobre el punto que marca el centro de la primera cabeza metatarsal y el otro brazo paralelo a la bisectriz de la falange proximal del hallux en su máxima flexión.

Para calcular el IPP, se seguirá el protocolo validado de la FPI-6 (Foot Posture Index-6 ítems), el cual cuantifica la puntuación de tres criterios en la parte posterior del pie: (96)

- Palpación de la cabeza del astrágalo: Es el único criterio de palpación. La cabeza del astrágalo se palpará en el lado medial y lateral de la cara anterior del tobillo. Cuanto mayor pronación, más palpable será la cabeza en el lado medial y menos en el lado lateral. En el pie supinado sucederá lo contrario.

- Curvatura supra e infra-maleolar lateral: Observación y comparación de las curvas por encima y por debajo del maléolo lateral del tobillo. En el pie pronado, la curva por debajo del maléolo será más aguda que la curva por encima del maléolo. En el pie supinado sucederá lo contrario.

- Posición del calcáneo en el plano frontal: Esto es un equivalente a la observación de la posición relajada del calcáneo en estática, que se mide utilizando un goniómetro. 0º indicará un pie neutro. El calcáneo estará claramente proando cuando se obtengan más de cinco grados hacia eversión. Más de cinco grados invertidos indicarán claramente supinación.

Además, hay tres criterios cuantificables en la parte anterior del pie:

- La prominencia en la región de la articulación talonavicular (ATN): En el pie neutro, el área de la piel inmediatamente superficial al ATN será plana. La ATN se hará más prominente si la cabeza del astrágalo se aduce en la parte posterior del pie pronado. En el pie supinado, este área será cóncava.

- La congruencia del arco longitudinal medial (ALM): Esta observación se realizará teniendo en cuenta, tanto la altura del arco como su congruencia. En un pie neutro, la curvatura del arco se considera similar a un segmento circunferencial. En el pie pronado, el ALM estará aplanado en el centro. En el pie supinado, la curvatura del ALM se agudizará en el extremo posterior del arco.

- Abducción / aducción del antepié en relación al retropié: En esta observación, el explorador valorará la parte delantera del pie directamente desde atrás, y en línea con el eje del talón. En el pie pronado, la parte anterior del pie será más visible por el lado lateral (el " signo de demasiados dedos"). En el pie supinado, la parte anterior del pie será más visible por el lado medial.

Cada ítem se calificará con una escala tipo Likert de cinco puntos, del -2 al +2 : Donde 0 será un pie neutro, puntuaciones de -2 indicarán señales claras de supinación, y de +2 indicarán signos positivos de pronación .

La puntuación final del IPP será un número entero entre -12 y +12. Una puntuación total de IPP entre 0 y 5 indicará un pie neutro, una puntuación por encima del +6 indicará un pie pronado o altamente pronado, y una puntuación entre -1 y -12 indicará un pie supinado o altamente supinado.

Así mismo, se recogerían también la presencia de deformidades digitales diferenciando dedo en garra, dedo en martillo, Hallux Abductus Valgus y Taylor Bunion.

La recogida de las presiones plantares tanto en estática como en dinámica se realizarán mediante un sistema de análisis podobarográfico computerizado, mediante el sistema F-Scan®, determinando los puntos de presión máxima, la presión media y la integral presión tiempo en 10 áreas de apoyó del pie dividida en talón medial, talón lateral, mediopié, cabezas de los metatarsianos del primero al quinto, primer dedo y zona de apoyo de los dedos medios del segundo al quinto.

Tras comprobar por parte del investigador que el calzado terapéutico es adecuado, se llevarán a cabo 3 registros de presiones plantares con el paciente descalzo, y otros 3 registros con el calzado terapéutico (con o sin suela rígida en balancín), según el brazo de estudio. Antes de la adquisición de los datos, los sujetos serán instruidos para caminar libremente en el laboratorio y sobre la plataforma, para reproducir su movimiento normal y sentirse cómodos en el laboratorio durante un período aproximado de cinco minutos. (97)

Todos los pacientes serán instruidos para rellenarán un cuestionario sobre la actividad física en los últimos 7 días, que se adjunta en el anexo 3. Dicho cuestionario será rellanado en una única ocasión y a la finalización del periodo de seguimiento. (98)

Los pacientes serán evaluados mensualmente de acuerdo a las recomendaciones del Grupo de Trabajo Internacional sobre el Pie Diabético (IWGDF) y hasta completar los 6 meses de seguimiento del estudio, para identificar posibles nuevas lesiones y realizar tratamientos preventivos. (99)

La recogida de datos se realizará mediante la cumplimentación del formulario que se adjunta en el anexo 4 del presente proyecto.

Una vez recogidas en el formulario las variables de estudio (ver anexo 5) se introducirán en el formato digital del mismo creado en el paquete estadístico Spss v21.0 para Macintosh (SPSS Inc., Chicago, IL). donde se realizará un análisis comparativo de los dos grupos, de cara a identificar la asociación entre reulceración y el uso de la suela rígida en balancín las variables generales, biomecánicas y los rangos de presiones plantares. Para analizar las asociaciones entre estas variables se realizará el test del chi^2. Una vez identificados los odds ratio (OR) de cada variable anteriormente interpretada, se construirá un modelo de regresión multivariante para valorar la influencia de manera independiente de cada variable,

teniendo en cuenta las interacciones entre las variables así como el ajuste del modelo por las variables confusoras.

Además, se elaborará una curva ROC para seleccionar el punto de corte óptimo de diagnóstico para predecir el riesgo de reulceración en la escala de mediciones de las presiones plantares. Para todos estos test se asumirán diferencias significativas en valores de $p<0.05$.

En resumen, las variables a estudio planteadas son:

Variable primaria:

- Tasa de recurrencia de lesión plantar en el pie diabético en pacientes tratados con calzado terapéutico con o sin suela rígida en balancín.

Variables secundarias:

- Definición de las deformidades estructurales del paciente diabético neuropático que se asocian a un mayor riesgo de reulceración en el pie.

- Valoración de limitaciones en el rango de movilidad articular de las articulaciones tibioperonea-astragalina, subastragalina y primera articulación metatarsofalángica en los pacientes diabéticos neuropáticos que se asocian a un mayor riesgo de reulceración en el pie.

- Definición de los patrones de presiones plantares que se asocian con el riesgo de reulceración en el paciente diabético neuropático y su control con el uso de la suela rígida en balancín.

- Describir el tiempo de uso del calzado terapéutico por parte del paciente diabético neuropático así como el impacto del mismo en el riesgo de reulceración y las variables que influyen en el tiempo de uso.

Las limitaciones de nuestro estudio radican principalmente en el sesgo de selección de los pacientes ya que son pacientes que acuden a la Unidad de Pie Diabético de la Universidad Complutense de Madrid con un motivo determinado de consulta relacionado con un problema patológico del pie. Son menores los que acuden por prevención y cuidado del mismo pie.

Cronograma y Plan de Trabajo

Se pretende reclutar 138 pacientes para el estudio en un período de tres años y con un seguimiento a 6 meses.

El calendario de reuniones del equipo investigador para el análisis de los resultados y revisión y discusión de los informes realizados será el siguiente:

- Semanalmente reunión del investigador principal y los Investigadores colaboradores de campo.
- Mensualmente revisión de los formularios de recogida de datos y comprobación de concordancias en el registro del SPSS v21.0.
- A los 3 meses análisis estadístico para identificar y valorar el desarrollo del estudio y sesgo de los pacientes. Se realizará una primera y segunda publicación incluyendo los resultados de los estudios preliminares de cara a crear expectativas nuevas en publicaciones internacionales y comparación con guías internacionales previas.
- A los 6 meses finalización del proyecto realizándose la memoria final donde se incluirán título del proyecto, objetivos, métodos, resultados, discusión y conclusiones, así como la justificación económica.

De esta forma, el proyecto se desarrollará en tres fases:

- Primera fase: Estudio de las características del pie diabético neuropático con antecedentes de úlceras plantares.
- Segunda fase: Identificar las alteraciones generales y biomecánicas de los pacientes diabéticos neuropáticos y su asociación con el riesgo de recurrencia de lesión.
- Tercera fase: Investigar la eficacia del calzado terapéutico con suela rígida en balancín en la reducción del riesgo de recurrencia de lesiones plantares en pacientes diabéticos neuropáticos.

(Las visitas de seguimiento de los pacientes se realizarán mensualmente, desde que comiencen a utilizar el calzado terapéutico y durante 6 meses. Si durante el período de seguimiento, el paciente presentase reulceración en el pie, será registrado en la variable de recurrencia de lesión para el cálculo final de tasa de recurrencia)

Experiencia del equipo investigador sobre el tema

El equipo de investigación que solicita la presente ayuda de financiación sanitaria está integrado en un equipo de atención multidisciplinar de la Unidad de Pie Diabético de la Clínica Universitaria de Podología de la Universidad Complutense de Madrid.

La Unidad de Pie Diabético de la Universidad Complutense de Madrid realiza además de la labor asistencial, una labor docente con los alumnos de grado de la Diplomatura, la formación de becarios postgraduados en un programa de residencia y la formación continua de profesionales sanitarios de diferentes profesiones y especialidades con implicación en el tratamiento del síndrome del Pie Diabético.

La Unidad de Pie Diabético de la Universidad Complutense de Madrid forma parte de la Asociación Científica Nacional denominada Grupo de Estudio Interdisciplinar de Pie Diabético, la cuál promociona de forma activa reuniones científicas y de investigación para mejorar la atención del síndrome del Pie Diabético en nuestro país y reducir el número de amputaciones que se asocia a esta complicación de la Diabetes *mellitus*.

En el campo específico de pie diabético, los miembros del grupo investigador ya han publicado en estos últimos cinco años más de 40 publicaciones en revistas de alto impacto internacional, comunicado estudios preliminares en torno a este tema en congresos nacionales e internacionales y realizado tesis doctorales. Además, desarrollan de forma habitual su práctica asistencial en la Clínica Universitaria de Podología de la Universidad Complutense integrados en la Unidad de Pie Diabético.

Algunas de estas publicaciones son:

2016:

- ✓ Molines-Barroso RJ, Lázaro-Martínez JL, Álvaro-Afonso FJ, Sanz-Corbalán I, García-Klepzig JL, Aragón-Sánchez J. Validation of an algorithm to predict reulceration in

amputation patients with diabetes. Int Wound J. 2016 Aug 9. doi: 10.1111/iwj.12639. JCR (2015) Factor de impacto: 2,59. Quartil: Q1, Ranking: 13 de 61. Categoría: Dermatología.

- ✓ Molines-Barroso RJ, Lázaro-Martínez JL, Aragón-Sánchez FJ, Álvaro-Afonso FJ, García-Morales E, García-Álvarez Y. Forefoot ulcer risk is associated with foot type in patients with diabetes and neuropathy. Diabetes Res Clin Pract. 2016 Jan 14. pii: S0168-8227(16)00057-7. doi: 10.1016/j.diabres.2016.01.008. JCR (2015) Factor de impacto: 3,04. Quartil: Q2, Ranking: 63 de 131. Categoría: Endocrinología y Metabolismo.

2015:

- ✓ Álvaro-Afonso FJ, Lázaro-Martínez JL, Aragón-Sánchez J, García-Morales E, García-Álvarez Y, Molines-Barroso RJ. What Is the Clinical Utility of the Ankle-Brachial Index in Patients With Diabetic Foot Ulcers and Radiographic Arterial Calcification? Int J Low Extrem Wounds. 2015 Dec;14(4):372-6. JCR (2015) Factor de impacto: 1,36. Quartil: Q3, Ranking: 39 de 61. Categoría: Dermatología.

- ✓ Sanz-Corbalán I, Lázaro-Martínez JL, Aragón-Sánchez J, García-Morales E, Molines-Barroso R, Alvaro-Afonso FJ. Analysis of Ulcer Recurrences After Metatarsal Head Resection in Patients Who Underwent Surgery to Treat Diabetic Foot Osteomyelitis. Int J Low Extrem Wounds. 2015 Jun;14(2):154-9. JCR (2015) Factor de impacto: 1,36. Quartil: Q3, Ranking: 39 de 61. Categoría: Dermatología.

Aragón-Sánchez J, Lázaro-Martínez JL, Alvaro-Afonso FJ, Molinés-Barroso R. Conservative Surgery of Diabetic Forefoot Osteomyelitis: How Can I Operate on This Patient Without Amputation? Int J Low Extrem Wounds. 2015 Jun;14(2):108-31. JCR (2015) Factor de impacto: 1,36. Quartil: Q3, Ranking: 39 de 61. Categoría: Dermatología.

2014:

- ✓ Armstrong DG, Hanft JR, Driver VR, Smith AP, Lazaro-Martinez JL, Reyzelman AM, Furst GJ, Vayser DJ, Cervantes HL, Snyder RJ, Moore MF, May PE, Nelson JL, Baggs GE, Voss AC; the Diabetic Foot Nutrition Study Group. Effect of oral nutritional supplementation on wound healing in diabetic foot ulcers: a prospective randomized controlled trial. Diabet Med. 2014 May 28. doi: 10.1111/dme.12509. [Epub ahead of print]. JCR (2015) Factor de impacto:3,15. Quartil: Q2, Ranking: 56 de 131. Categoría: Endocrinología y Metabolismo.

- ✓ Alvaro-Afonso FJ, Lázaro-Martínez JL, Aragón-Sánchez J, García-Morales E, García-Álvarez Y, Molines-Barroso RJ. Inter-observer reproducibility of diagnosis of diabetic foot osteomyelitis based on a combination of probe-to-bone test and simple radiography. Diabetes Res Clin Pract. 2014 Jul; 105(1):e3-5. doi: 10.1016/j.diabres.2014.04.024. Epub 2014 Apr 28. JCR (2015) Factor de impacto: 3,04. Quartil: Q2, Ranking: 63 de 131. Categoría: Endocrinología y Metabolismo.

- ✓ Lázaro-Martínez JL, Aragón-Sánchez J, García-Morales E. Response to comment on Lazaro-Martinez et al. Antibiotics versus conservative surgery for treating diabetic foot osteomyelitis: a randomized comparative trial. Diabetes Care 2014; 37:789-795. Diabetes Care. 2014 May; 37(5):e116-7. doi: 10.2337/dc14-0114. JCR (2015) Factor de impacto: 8,93. Quartil: Q1, Ranking: 7 de 131. Categoría: Endocrinología y Metabolismo.

- ✓ Aragón-Sánchez J, Lázaro-Martínez JL, García-Álvarez Y, Morales EG, Hernández-Herrero MJ. Albuminuria is a predictive factor of in-hospital mortality in patients with diabetes admitted for foot disease. Diabetes Res Clin Pract. 2014 Jan 21. pii: S0168-8227(14)00008-4. doi: 10.1016/j.diabres.2014.01.006. JCR (2015) Factor de impacto: 3,04. Quartil: Q2, Ranking: 63 de 131. Categoría: Endocrinología y Metabolismo.

- ✓ Lázaro-Martínez J, Aragón-Sánchez J, García-Morales E. Antibiotics versus conservative surgery for treating diabetic foot osteomyelitis. A randomized comparative trial. Diabetes Care.2014 Mar; 37(3):789-95. JCR (2015) Factor de impacto: 8,93. Quartil: Q1, Ranking: 7 de 131. Categoría: Endocrinología y Metabolismo.

2013:

- ✓ Cecilia-Matilla A, Lázaro-Martínez JL, Aragón-Sánchez J, García-Morales E, García-Álvarez Y, Beneit-Montesinos JV. Histopathologic characteristics of bone infection complicating foot ulcers in diabetic patients. J Am Podiatr Med Assoc. 2013 Jan-Feb; 103(1):24-31. JCR (2015) Factor de impacto: 0,48. Quartil: Q4, Ranking: 68 de 74. Categoría: Ortopedia.

- ✓ Sanz-Corbalán I, Lázaro-Martínez JL, García-Morales E, Aragón-Sánchez J, Carabantes-Alarcón D, García-Álvarez Y. Relationship of limited joint mobility and foot deformities with neurological examination in patients with diabetes. Exp Clin Endocrinol Diabetes. 2013 Apr; 121(4):239-43. doi: 10.1055/s-0032-1329981. Epub 2013 Jan 17. JCR (2015) Factor de impacto: 1,66. Quartil: Q4, Ranking: 105 de 131. Categoría: Endocrinología y Metabolismo.

- ✓ Álvaro-Afonso FJ, Lázaro-Martínez JL, Aragón-Sánchez J, García-Morales E, Cecilia-Matilla A, Beneit-Montesinos JV. Interobserver and intraobserver reproducibility of plain X-rays in the diagnosis of diabetic foot osteomyelitis. Int J Low Extrem Wounds. 2013 Mar; 12(1):12-5. Doi: 10.1177/1534734612474304. Epub 2013 Feb 1. JCR (2015) Factor de impacto: 1,36. Quartil: Q3, Ranking: 39 de 61. Categoría: Dermatología.

- ✓ Aragón-Sánchez J, Lázaro-Martínez JL, Quintana-Marrero Y, Sanz-Corbalán I, Hernández-Herrero MJ, Cabrera-Galván JJ. Super-oxidized solution (dermacyn wound care) as adjuvant treatment in the postoperative management of complicated diabetic foot osteomyelitis: preliminary experience in a specialized department. Int J Low Extrem Wounds. 2013 Jun; 12(2):130-7. Doi: 10.1177/1534734613476710. Epub 2013 Feb 26. JCR (2015) Factor de impacto: 1,36. Quartil: Q3, Ranking: 39 de 61. Categoría: Dermatología.

- ✓ Aragón-Sánchez J, Lipsky BA, Lázaro-Martínez JL. Gram-negative diabetic foot osteomyelitis: risk factors and clinical presentation. Int J Low Extrem Wounds. 2013 Mar; 12(1):63-8. Doi: 10.1177/1534734613477423. Epub 2013 Feb 26. JCR (2015) Factor de impacto: 1,36. Quartil: Q3, Ranking: 39 de 61. Categoría: Dermatología.

- ✓ Aragón-Sánchez J, Lázaro-Martínez JL, Quintana-Marrero Y, Alvaro-Afonso FJ, Hernández-Herrero MJ. Charcot neuroarthropathy triggered and complicated by osteomyelitis. How limb salvage can be achieved. Diabet Med. 2013 Jun; 30(6):e229-32. doi: 10.1111/dme.12191. Epub 2013 Apr 17. JCR (2015) Factor de impacto:3,15. Quartil: Q2, Ranking: 56 de 131. Categoría: Endocrinología y Metabolismo.

- ✓ Molines-Barroso RJ, Lázaro-Martínez JL, Aragón-Sánchez J, García-Morales E, Beneit-Montesinos JV, Alvaro-Afonso FJ. Analysis of transfer lesions in patients who underwent surgery for diabetic foot ulcers located on the plantar aspect of the metatarsal heads. Diabet Med. 2013 Aug; 30(8):973-6. Doi: 10.1111/dme.12202. Epub 2013 Apr 20. JCR (2015) Factor de impacto:3,15. Quartil: Q2, Ranking: 56 de 131. Categoría: Endocrinología y Metabolismo.

- ✓ Aragón-Sánchez J, Lázaro-Martínez JL, Molinés-Barroso R, García Álvarez Y, Quintana-Marrero Y, Hernández-Herrero MJ. Revision surgery for diabetic foot infections: giving another chance to the patient. Int J Low Extrem Wounds. 2013 Jun; 12(2):146-51. Doi: 10.1177/1534734613486155. Epub 2013 May 13. JCR (2015) Factor de impacto: 1,36. Quartil: Q3, Ranking: 39 de 61. Categoría: Dermatología.

- ✓ García-Álvarez Y, Lázaro-Martínez JL, García-Morales E, Cecilia-Matilla A, Aragón-Sánchez J, Carabantes-Alarcón D. Morphofunctional characteristics of the foot in patients with diabetes mellitus and diabetic neuropathy. Diabetes Metab Syndr. 2013 Apr-Jun; 7(2):78-82. doi: 10.1016/j.dsx.2013.02.029. Epub 2013 Mar 15.

- ✓ Cecilia-Matilla A, Lázaro-Martínez JL, Aragón-Sánchez J. Dear Editor. J Foot Ankle Surg. 2013 Jun 12. Doi: pii: S1067-2516(13)00196-8. 10.1053/j.jfas.2013.05.003. JCR (2015) Factor de impacto: 1,09. Quartil: Q3, Ranking: 49 de 74. Categoría: Ortopedia.
- ✓
- ✓ Alvaro-Afonso FJ, Lázaro-Martínez JL, Aragón-Sánchez FJ, García-Morales E, Carabantes-Alarcón D, Molines-Barroso RJ. Does the location of the ulcer affect the interpretation of the probe-to-bone test in the diagnosis of osteomyelitis in diabetic foot ulcers? Diabet Med. 2013 Jul 19. Doi: 10.1111/dme.12280. JCR (2015) Factor de impacto:3,15. Quartil: Q2, Ranking: 56 de 131. Categoría: Endocrinología y Metabolismo.

- ✓ Aragón-Sánchez J, Lázaro-Martínez JL, Cabrera-Galván JJ. Additional information on the role of histopathology in diagnosing diabetic foot osteomyelitis. Diabet Med. 2013 Jul 22. Doi: 10.1111/dme.12283. JCR (2015) Factor de impacto:3,15. Quartil: Q2, Ranking: 56 de 131. Categoría: Endocrinología y Metabolismo.

- ✓ Cecilia-Matilla A, Lázaro-Martínez JL, Aragón-Sánchez J, García-Álvarez Y, Chana-Valero P, Beneit-Montesinos JV. Influence of the Location of Nonischemic Diabetic Forefoot Osteomyelitis on Time to Healing After Undergoing Surgery. Int J Low Extrem Wounds.

2013 Sep 16. JCR (2015) Factor de impacto: 1,36. Quartil: Q3, Ranking: 39 de 61. Categoría: Dermatología.

- ✓ Rubio JA, Aragón-Sánchez J, Lázaro-Martínez JL, Almaraz MC, Mauricio D, Antolín Santos JB, Díaz Pérez JA, Fabbi M, Lozano Del Hoyo ML, Vela MP; en representación del Grupo Español del Pie Diabético. Diabetic foot units in Spain: Knowing the facts using a questionnaire. Endocrinol Nutr. 2013 Nov 4. Doi: pii: S1575-0922(13)00269-6. 10.1016/j.endonu.2013.07.002. English, Spanish.

- ✓ Aragón-Sánchez J, Lázaro-Martínez JL. Factors Associated With Calcification in the Pedal Arteries in Patients With Diabetes and Neuropathy Admitted for Foot Disease and Its Clinical Significance. Int J Low Extrem Wounds. 2013 Nov 25. JCR (2015) Factor de impacto: 1,36. Quartil: Q3, Ranking: 39 de 61. Categoría: Dermatología.

- ✓ Lázaro-Martínez JL, Cecilia-Matilla A, Aragón-Sánchez J, García-Morales E, Garcia-Alvarez Y, Alvaro-Afonso F. Treatment of infected diabetic foot ulcers clinical effectiveness of a dressing of alginate and hydrocolloid, with silver fiber. Analysis of results of a series of cases. Rev Enferm. 2013 Nov; 36(11):29-34.

2012:

- ✓ Aragón-Sánchez J, Lázaro-Martínez JL, Hernández-Herrero C, Campillo-Vilorio N, Quintana-Marrero Y, García-Morales E, Hernández-Herrero MJ. Does osteomyelitis in the feet of patients with diabetes really recur after surgical treatment? Natural history of a surgical series. Diabet Med. 2012 Jun;29(6):813-8. doi: 10.1111/j.1464-5491.2011.03528.x. JCR (2015) Factor de impacto:3,15. Quartil: Q2, Ranking: 56 de 131. Categoría: Endocrinología y Metabolismo.

- ✓ Aragón-Sánchez J, Lázaro-Martínez JL, Cecilia-Matilla A, Quintana-Marrero Y, Hernández-Herrero MJ. Limb salvage for spreading midfoot osteomyelitis following diabetic foot surgery. J Tissue Viability. 2012 May;21(2):64-70. Epub 2012 Jan 9. JCR (2015) Factor de impacto: 1,65. Quartil: Q1, Ranking: 19 de 116. Categoría: Enfermería.

- ✓ García-Morales E, Lázaro-Martínez JL, Aragón-Sánchez J, Cecilia-Matilla A, García-Álvarez Y, Beneit-Montesinos JV. Surgical complications associated with primary closure in patients with diabetic foot osteomyelitis. Diabet Foot Ankle. 2012; 3. Doi: 10.3402/dfa.v3i0.19000. Epub 2012 Sep 25.

- ✓ Aragón-Sánchez J, Lázaro-Martínez JL, Pulido-Duque J, Maynar M. From the diabetic foot ulcer and beyond: how do foot infections spread in patients with diabetes? Diabet Foot Ankle. 2012;3. doi: 10.3402/dfa.v3i0.18693. Epub 2012 Oct 1.

- ✓ Aragón-Sánchez J, Lázaro-Martínez JL, Campillo-Vilorio N, Quintana-Marrero Y, Hernández-Herrero MJ. Controversies regarding radiological changes and variables predicting amputation in a surgical series of diabetic foot osteomyelitis. Foot Ankle Surg. 2012 Dec;18(4):233-6. doi: 10.1016/j.fas.2012.01.005. Epub 2012 Mar 2. JCR (2015) Factor de impacto: 1,09. Quartil: Q3, Ranking: 49 de 74. Categoría: Ortopedia.

2011:

- ✓ Aragon-Sanchez J, Lipsky BA, Lazaro-Martinez JL. Diagnosing diabetic foot osteomyelitis: is the combination of probe-to-bone test and plain radiography sufficient for high-risk inpatients? Diabet Med. 2011 Feb;28(2):191-4. JCR (2015) Factor de impacto:3,15. Quartil: Q2, Ranking: 56 de 131. Categoría: Endocrinología y Metabolismo.

- ✓ Garcia-Morales E, Lazaro-Martinez JL, Martinez-Hernandez D, Aragon-Sanchez J, Beneit-Montesinos JV, Gonzalez-Jurado MA. Impact of diabetic foot related complications on the Health Related Quality of Life (HRQol) of patients--a regional study in Spain. Int J Low Extrem Wounds. 2011 Mar;10(1):6-11. JCR (2015) Factor de impacto: 1,36. Quartil: Q3, Ranking: 39 de 61. Categoría: Dermatología.

- ✓ Lazaro-Martinez JL, Aragon-Sanchez FJ, Beneit-Montesinos JV, Gonzalez-Jurado MA, Garcia Morales E, Martinez Hernandez D. Foot biomechanics in patients with diabetes mellitus: doubts regarding the relationship between neuropathy, foot motion, and deformities. J Am Podiatr Med Assoc. May-Jun;101(3):208-14. JCR (2015) Factor de impacto: 0,48. Quartil: Q4, Ranking: 68 de 74. Categoría: Ortopedia

- ✓ Garcia Morales E, Lazaro-Martinez JL, Aragon-Sanchez FJ, Cecilia-Matilla A, Beneit-Montesinos JV, Gonzalez Jurado MA. Inter-observer reproducibility of probing to bone in the diagnosis of diabetic foot osteomyelitis. Diabet Med. 2011 Oct;28(10):1238-40. JCR (2015) Factor de impacto:3,15. Quartil: Q2, Ranking: 56 de 131. Categoría: Endocrinología y Metabolismo.

- ✓ Aragon-Sanchez J, Lazaro-Martinez JL. Comment on: Lipsky et al. Developing and validating a risk score for lower-extremity amputation in patients hospitalized for a diabetic foot infection. Diabetes Care 2011;34:1695-1700. Diabetes Care. Oct;34(10):e160; author reply e1. JCR (2015) Factor de impacto: 8,93. Quartil: Q1, Ranking: 7 de 131. Categoría: Endocrinología y Metabolismo.
- ✓ Aragon-Sanchez J, Jimenez FC, Lazaro-Martinez JL, Campillo-Vilorio N, Quintana-Marrero Y, Hernandez-Herrero MJ. Never Amputate a Patient With Diabetes Without Consulting With a Specialized Unit. Int J Low Extrem Wounds. 2011 Dec;10(4):214-7. JCR (2015) Factor de impacto: 1,36. Quartil: Q3, Ranking: 39 de 61. Categoría: Dermatología.

- ✓ Aragon-Sanchez J, Lazaro-Martinez JL. Impact of perioperative glycaemia and glycated haemoglobin on the outcomes of the surgical treatment of diabetic foot osteomyelitis. Diabetes Res Clin Pract. 2011 Dec; 94(3):83-5. JCR (2015) Factor de impacto: 3,04. Quartil: Q2, Ranking: 63 de 131. Categoría: Endocrinología y Metabolismo.

- ✓ Aragon-Sanchez J, Lazaro-Martinez JL, Hernandez-Herrero C, Campillo-Vilorio N, Quintana-Marrero Y, Garcia-Morales E, et al. Surgical Treatment of Limb- and Life-Threatening Infections in the Feet of Patients With Diabetes and at Least One Palpable Pedal Pulse: Successes and Lessons Learnt. Int J Low Extrem Wounds. 2011 Dec;10(4):207-13. JCR (2015) Factor de impacto: 1,36. Quartil: Q3, Ranking: 39 de 61. Categoría: Dermatología.

- ✓ Aragon-Sanchez J, Lipsky BA, Lazaro-Martinez JL. Diagnosing diabetic foot osteomyelitis: is the combination of probe-to-bone test and plain radiography sufficient for high-risk inpatients? Diabet Med. 2011 Feb;28(2):191-4. JCR (2015) Factor de impacto:3,15. Quartil: Q2, Ranking: 56 de 131. Categoría: Endocrinología y Metabolismo.

- ✓ Garcia-Morales E, Lazaro-Martinez JL, Martinez-Hernandez D, Aragon-Sanchez J, Beneit-Montesinos JV, Gonzalez-Jurado MA. Impact of diabetic foot related complications on the Health Related Quality of Life (HRQol) of patients--a regional study in Spain. Int J Low Extrem Wounds. 2011 Mar;10(1):6-11. JCR (2015) Factor de impacto: 1,36. Quartil: Q3, Ranking: 39 de 61. Categoría: Dermatología.

- ✓ Lazaro-Martinez JL, Aragon-Sanchez FJ, Beneit-Montesinos JV, Gonzalez-Jurado MA, Garcia Morales E, Martinez Hernandez D. Foot biomechanics in patients with diabetes mellitus: doubts regarding the relationship between neuropathy, foot motion, and deformities. J Am Podiatr Med Assoc. May-Jun;101(3):208-14. JCR (2015) Factor de impacto: 0,48. Quartil: Q4, Ranking: 68 de 74. Categoría: Ortopedia

- ✓ Aragon-Sanchez J, Jimenez FC, Lazaro-Martinez JL, Campillo-Vilorio N, Quintana-Marrero Y, Hernandez-Herrero MJ. Never Amputate a Patient With Diabetes Without Consulting With a Specialized Unit. Int J Low Extrem Wounds. 2011 Dec;10(4):214-7. JCR (2015) Factor de impacto: 1,36. Quartil: Q3, Ranking: 39 de 61. Categoría: Dermatología.

- ✓ Aragon-Sanchez J, Lazaro-Martinez JL, Hernandez-Herrero C, Campillo-Vilorio N, Quintana-Marrero Y, Garcia-Morales E, et al. Surgical Treatment of Limb- and Life-Threatening Infections in the Feet of Patients With Diabetes and at Least One Palpable Pedal Pulse: Successes and Lessons Learnt. Int J Low Extrem Wounds. 2011 Dec;10(4):207-13. JCR (2015) Factor de impacto: 1,36. Quartil: Q3, Ranking: 39 de 61. Categoría: Dermatología.

2010:

- ✓ Lazaro-Martinez JL, Garcia-Morales EA, Aragon-Sanchez FJ, Ano-Vidales P, Allas-Aguado S, Garcia-Alvarez Y. [Reducing skin maceration in exudative diabetic foot ulcers]. Rev Enferm. 2010 Mar;33(3):9-14.

- ✓ Aragon-Sanchez J, Hernandez-Herrero MJ, Lazaro-Martinez JL, Quintana-Marrero Y, Maynar-Moliner M, Rabellino M, et al. In-hospital complications and mortality following major lower extremity amputations in a series of predominantly diabetic patients. Int J Low Extrem Wounds. 2010 Mar;9(1):16-23. JCR (2015) Factor de impacto: 1,36. Quartil: Q3, Ranking: 39 de 61. Categoría: Dermatología.

- ✓ Aragon-Sanchez J, Lazaro-Martinez JL, Hernandez-Herrero MJ, Quintana-Marrero Y, Cabrera-Galvan JJ. Clinical significance of the isolation of Staphylococcus epidermidis from bone biopsy in diabetic foot osteomyelitis. Diabet Foot Ankle. 2010.1 doi: 10.3402/dfa.v1i0.5418.

- ✓ Aragón-Sánchez J, Lázaro-Martínez JL, Hernández-Herrero MJ. Triggering mechanisms of neuroarthropathy following conservative surgery for osteomyelitis. Diabet Med. 2010 Jul;27(7):844-7. JCR (2015) Factor de impacto:3,15. Quartil: Q2, Ranking: 56 de 131. Categoría: Endocrinología y Metabolismo.

En lo que se refiere a investigación, la actividad desempeñada en esta unidad está siendo el germen de importantes líneas de investigación en el marco del Master Oficial de Postgrado: Investigación en Cuidados, de la Facultad Universitaria de Enfermería, Fisioterapia y Podología.

En la actualidad posee las siguientes líneas de investigación:

1. “Osteomielitis en el pie diabético”
2. “Alteraciones biomecánicas en los pacientes con y sin neuropatía diabética”
3. “Identificación de los factores de riesgo de ulceración de los pacientes diabéticos con y sin neuropatía mediante el análisis de la patología podológica y biomecánica”
4. “Estudio de valoración y clasificación de factores pronósticos en la cicatrización de la úlcera neuropática”
5. “Eficacia de las microcorrientes en la cicatrización de las úlceras neuroisquémicas en el pie diabético”
6. “Eficacia y efectividad de diferentes soportes plantares en la reducción local de presiones plantares”

Se han presentado fruto de estas líneas de investigación las siguientes tesis:

- Jose Luis Lázaro Martínez: “Determinación de las Características Biomecánicas en el pie Diabético con y sin neuropatía”.

- María Luz González Fernández: “Estudio del tratamiento ortopodológico individualizado del paciente diabético de alto riesgo y su efecto sobre la incidencia de la reulceración”.

- María del Rosario Morales Lozano: “Validación de la prueba del probing to bone frente a otras pruebas de diagnóstico clínico de la osteomielitis crónica en el pie diabético”.

- Gabriel Rivera: “Estudio de la anatomía osteoarticular del pie mediante el análisis goniométrico en radiografía simple en pacientes diabéticos con y sin neuropatía”.

- Laura Ramos: “Estudio de la influencia de la limitación de la movilidad articular en el desarrollo de patrones de sobrecarga en el pie diabético con y sin neuropatía”.

- Juan Pedro Sánchez Ríos: "Eficacia de los Ácidos Grasos Hiperoxigenados en la microcirculación de pacientes con Pie Diabético"

- Almudena Cecilia Matilla: "Determinación histopatológica de la osteomielitis y su influencia en el pronóstico de la cicatrización en pacientes con úlceras de pie diabético"

- Yolanda García Álvarez: "Identificación de las deformidades morfofuncionales del pie en pacientes diabéticos con o sin neuropatía y en pacientes no diabéticos mediante el análisis de la patología podológica y biomecánica".

- Francisco Javier Álvaro Afonso: "Variabilidad diagnóstica de la palpación transulcerosa de hueso (Probe to bone) y de la radiografía simple en el diagnóstico de osteomielitis en úlceras de pie diabético".

- Silvia Allas Aguado: "Estudio clínico aleatorizado sobre la efectividad del tratamiento médico frente al quirúrgico en la osteomielitis asociada a lesiones de pie diabético".

- Raúl Juan Molines Barroso: "Riesgo de reulceración tras la resección quirúrgica de una cabeza metatarsal en el paciente con Diabetes mellitus".

- Javier Hernández Toledo: "Las bacterias Gram negativas y su influencia en la cicatrización de úlceras de pie diabético".

- Irene Sanz Corbalán: "Utilidad del test de la función sudomotora como instrumento clínico de clasificación de riesgo del paciente diabético".

El Laboratorio Podartis es actualmente una de las más importantes empresas en fabricación de calzado terapéutico en la UE. La amplia experiencia, la capacidad de sus instalaciones para desarrollar todos los procesos de fabricación, la alta cualificación de su equipo humano, hace que se hayan convertido en referencia imprescindible en la investigación, diseño, elaboración y acabado del calzado terapéutico.

Plan de difusión:

1.- Relevancia del proyecto en cuanto a su impacto clínico, asistencial y / o desarrollo tecnológico

La viabilidad del proyecto permitirá alcanzar objetivos clínicos muy importantes en el campo de la prevención del síndrome del pie diabético, ya que se podrán definir de forma clara los factores de riesgo de ulceración que se relacionan con las alteraciones biomecánicas de los pacientes diabéticos y que desembocan en el aumento de presiones plantares y la formación de úlceras en el pie. Hasta el momento no se ha definido en el marco científico del pie diabético de forma clara esta implicación y existe a día de hoy una enorme controversia aún por resolver. Además, permitirá establecer tratamientos preventivos que contribuyan a reducir las tasas de reulceración y de amputación que actualmente constituyen un enorme problema social, sanitario y económico en nuestro país. La demostración objetiva de los beneficios de las tecnologías de los servicios de salud podológica en la atención del pie diabético es relevante de cara a facilitar a las Administraciones de Salud Públicas y Privadas la toma de decisiones a la hora de impulsar la creación de unidades multidisciplinares de atención al Pie Diabético en nuestro país.

2.- Relevancia del proyecto en cuanto a su impacto bibliométrico

El síndrome del Pie Diabético es un problema sanitario que motiva numerosas investigaciones en diferentes facetas. Numerosas publicaciones de prestigio científico incluyen de forma habitual investigaciones sobre el Pie Diabético como es el caso de Lancet, Diabetes Care, Diabetes Medicine, Clinical Biomechanics, Foot and Ankle International y JAPMA (Journal American Podiatric Medical Association). Se han publicado numerosos estudios respecto al tema de investigación propuesto en dichas publicaciones, pero no se ha realizado un estudio tan específico y preciso acerca de cómo y por qué se produce la úlcera en el pie diabético neuropático y de la aplicación terapéutica de un elemento tan específico como la suela rígida en balancín en el calzado terapéutico.

Medios disponibles para la realización del proyecto

La Unidad De Pie Diabético de la Clínica Universitaria de Podología de la Universidad Complutense de Madrid cuenta con los siguientes recursos físicos para la realización del proyecto:

- Gabinete habilitado para investigación donde se poseen los instrumentos necesarios para la exploración neurológica (biotensiómetro y monofilamentos), doppler informatizado e instrumento de medición de la presión transcutánea de oxígeno. Además el gabinete está habilitado con banco de marcha, camilla de exploración, podoscopio y reflexoscopio.

- El estudio pormenorizado de la biomecánica del paciente diabético requiere una inversión en tiempo y recursos que normalmente los pacientes que sufren esta enfermedad no pueden costearse pese a poder ofrecerse este servicio hoy en día en la clínica. La clínica dispone de la mayoría del material inventariable que se solicita en el siguiente proyecto salvo el sistema de plantillas instrumentadas recurso imprescindible para la realización de este proyecto.

El Laboratorio Podartis cuenta con modelos de calzado terapéutico para hombre (X-Diab-14) y mujer (Cortina Gran Volume), los cuales sólo se diferencian en el diseño de la suela, mientras que el resto de características externas del calzado son idénticas en ambos modelos (anexo 1).
Sus productos son testados clínicamente para garantizar el máximo beneficio y confort. Los modelos estarán disponibles en España en 48-72 horas desde su solicitud.
Podartis está especializado en la producción de calzado para la prevención del pie diabético y representa una empresa de referencia internacional, con una amplia experiencia desde su creación en 2001.

Bibliografía:

1. Conget I. [Diagnosis, classification and cathogenesis of diabetes mellitus]. Rev Esp Cardiol. 2002 May;55(5):528-35.
2. Boulton AJ, Vileikyte L, Ragnarson-Tennvall G, Apelqvist J. The global burden of diabetic foot disease. Lancet. 2005 Nov 12;366(9498):1719-24.
3. Wild S, Roglic G, Green A, Sicree R, King H. Global prevalence of diabetes: estimates for the year 2000 and projections for 2030. Diabetes Care. 2004 May;27(5):1047-53.
4. Singh N, Armstrong DG, Lipsky BA. Preventing foot ulcers in patients with diabetes. JAMA. 2005 Jan 12;293(2):217-28.
5. Instituto Nacional de Salud. Encuesta Nacional de Salud2006.
6. Delgado E. Las estimaciones internacionales indican que la prevalencia de diabetes se duplicará en los próximos años. Economía de la Salud. 2008;7(2):55-8.
7. Hogan P, Dall T, Nikolov P. Economic costs of diabetes in the US in 2002. Diabetes Care. 2003 Mar;26(3):917-32.
8. Ballesta M, Carral F, Grupo de diabetes de la SAEN. Costes directos e indirectos de la diabetes mellitus tipo 2. Avances en Diabetes. 2002;18 Supl(1):20.
9. Conget I. Un control exhaustivo de la diabetes reduce los costes asociados a las complicaciones. Economía de la Salud 2008.7(2):88.
10. O'Sullivan JB, Mahan CM. Mortality related to diabetes and blood glucose levels in a community study. Am J Epidemiol. 1982 Oct;116(4):678-84.
11. Frykberg RG. Epidemiology of the diabetic foot: ulcerations and amputations. Adv Wound Care. 1999 Apr;12(3):139-41.
12. Organización Mundial de la Salud. Prevención de la diabetes mellitus. Ginebra: Informe de un Grupo de Estudio de la OMS. Ser Inf Tecn. 1994;884:81.
13. Moss SE, Klein R, Klein BE. The 14-year incidence of lower-extremity amputations in a diabetic population. The Wisconsin Epidemiologic Study of Diabetic Retinopathy. Diabetes Care. 1999 Jun;22(6):951-9.
14. Carmona GA, Hoffmeyer P, Herrmann FR, Vaucher J, Tschopp O, Lacraz A, et al. Major lower limb amputations in the elderly observed over ten years: the role of diabetes and peripheral arterial disease. Diabetes Metab. 2005 Nov;31(5):449-54.
15. Glober JL, Weingarten MS, Buchbinder DS. A 4-year outcome-based retrospective study of wound healing and limb salvage in patients with chronic wounds. Adv Wound Care. 1997;10:33-7.
16. Levin ME. Preventing amputation in the patient with diabetes. Diabetes Care. 1995 Oct;18(10):1383-94.

17. Larsson J, Agardh CD, Apelqvist J, Stenstrom A. Long-term prognosis after healed amputation in patients with diabetes. Clin Orthop Relat Res. 1998 May(350):149-58.
18. Reiber GE, Boyko EJ, Smith DG. Lower extremity foot ulcers and amputations in diabetes. In: Harris MI, Cowie C, Stern MP, editors. Diabetes in America1995. p. 409-27.
19. Reiber GE, Boyko EJ, Smith DG. Lower extremity foot ulcers and amputations in diabetes. Diabetes in America 2nd ed. 1995:409-27.
20. Boulton AJ. The diabetic foot: from art to science. The 18th Camillo Golgi lecture. Diabetologia. 2004 Aug;47(8):1343-53.
21. Pecoraro RE, Reiber GE, Burgess EM. Pathways to diabetic limb amputation. Basis for prevention. Diabetes Care. 1990 May;13(5):513-21.
22. Knox RC, Dutch W, Blume P, Sumpio BE. Diabetic Foot Disease. Int J Angiol. 2000 Jan;9(1):1-6.
23. Mueller MJ, Diamond JE, Delitto A, Sinacore DR. Insensitivity, limited joint mobility, and plantar ulcers in patients with diabetes mellitus. Phys Ther. 1989 Jun;69(6):453-9; discussion 9-62.
24. American Diabetes Association. Clinical practice recommendations 1996. Diabetes Care 1996 Jan. 1996;19 (Suppl 1):S1-118.
25. Adler AI, Boyko EJ, Ahroni JH, Stensel V, Forsberg RC, Smith DG. Risk factors for diabetic peripheral sensory neuropathy. Results of the Seattle Prospective Diabetic Foot Study. Diabetes Care 1997 Jul. 1997;20(7):1162-7.
26. March JR, López-Quintana A, Guilleuma J. Diabetic foot management. In: Pharmacotherapy D, editor. Ros Die. Barcelona1998. p. 537-41.
27. Tomás P, Diabetic foot work group GEDAPS. Diabetic foot. Aten Primaria. 1996;18(10):533-4.
28. Gibbons GW. Lower extremity bypass in patients with diabetic foot ulcers. Surg Clin North Am. 2003 Jun;83(3):659-69.
29. Frykberg RG. An evidence-based approach to diabetic foot infections. Am J Surg. 2003 Nov 28;186(5A):44S-54S; discussion 61S-4S.
30. Serrano FJ, Martín A. Enfermedad arterial periférica: aspectos fisiopatológicos, clínicos y terapéuticos. Rev Esp Cardiol. 2007;60(9):969-82.
31. Kannel WB. Risk factors for atherosclerotic cardiovascular outcomes in different arterial territories. J Cardiovasc Risk. 1994 Dec;1(4):333-9.
32. Apelqvist J, Bakker K, van Houtum WH, Nabuurs-Franssen MH, Schaper NC. International consensus and practical guidelines on the management and the prevention of the diabetic foot. International Working Group on the Diabetic Foot. Diabetes Metab Res Rev. 2000 Sep-Oct;16 Suppl 1:S84-92.

33. Consensus Development Conference on Diabetic Foot Wound Care: 7-8 April 1999, Boston, Massachusetts. American Diabetes Association. Diabetes Care. 1999 Aug;22(8):1354-60.
34. Geerlings SE, Hoepelman AI. Immune dysfunction in patients with diabetes mellitus (DM). FEMS Immunol Med Microbiol. 1999 Dec;26(3-4):259-65.
35. Caputo GM. The rational use of antimicrobial agents in diabetic foot infection. In: Boulton AJM, Connor H, Cavanagh PR, Wiley and Sons, editors. The Foot in Diabetes. 3rd ed. Chichester1994. p. 143-51.
36. Eneroth M, Larsson J, Apelqvist J. Deep foot infections in patients with diabetes and foot ulcer: an entity with different characteristics, treatments, and prognosis. J Diabetes Complications. 1999 Sep-Dec;13(5-6):254-63.
37. Frykberg RG, Zgonis T, Armstrong DG, Driver VR, Giurini JM, Kravitz SR, et al. Diabetic foot disorders. A clinical practice guideline (2006 revision). J Foot Ankle Surg. 2006 Sep-Oct;45(5 Suppl):S1-66.
38. Caputo GM, Cavanagh PR, Ulbrecht JS, Gibbons GW, Karchmer AW. Assessment and management of foot disease in patients with diabetes. N Engl J Med. 1994 Sep 29;331(13):854-60.
39. Gibbons GW, Eliopoulus GM. Infection of the diabetic foot. In: Kozak GP, Campbell DR, Frykberg RG, Habershaw GM, Saunders WB, editors. Management of the Diabetic Foot Problems. Philadelphia1995. p. 121.
40. Karchmer AW, Gibbons GW. Foot infections in diabetes: evaluation and management. Curr Clin Top Infect Dis. 1994;14:1-22.
41. Eneroth M, Apelqvist J, Stenstrom A. Clinical characteristics and outcome in 223 diabetic patients with deep foot infections. Foot Ankle Int. 1997 Nov;18(11):716-22.
42. Fard AS, Esmaelzadeh M, Larijani B. Assessment and treatment of diabetic foot ulcer. Int J Clin Pract. 2007 Nov;61(11):1931-8.
43. Unger RF, Foster DW. Diabetes mellitus. In: Williams RH, Wilson JD, Foster DW, editors. Williams textbook of endocrinology. 8th ed. Philadelphia: WB Saundeers Company; 1992. p. 1255-333.
44. Edmonds ME, Blundell MP, Morris ME, Thomas EM, Cotton LT, Watkins PJ. Improved survival of the diabetic foot: the role of a specialized foot clinic. Q J Med. 1986 Aug;60(232):763-71.
45. Frykberg RG. Diabetic foot ulcers: pathogenesis and management. Am Fam Physician. 2002 Nov 1;66(9):1655-62.
46. Aksoy DY, Gurlek A, Cetinkaya Y, Oznur A, Yazici M, Ozgur F, Aydingoz U, Gedik O: Change in the amputation profile in diabetic foot in a tertiary reference center: efficacy of team working. Exp Clin Endocrinol Diabetes 112:526-530, 2004.

47. Stanley S, Turner L: A collaborative care approach to complex diabetic foot ulceration. Br J Nurs 13:788-793, 2004.
48. Sandnes DK, Sobel M, Flum DR: Survival after lower-extremity amputation. J Am Coll Surg 199:394-402, 2004.
49. Davis BL, Kuznicki J, Praveen SS, Sferra JJ: Lower-extremity amputations in patients with diabetes: pre- and post-surgical decisions related to successful rehabilitation. Diabetes Metab Res Rev 20 Suppl 1:S45-S50, 2004.
50. Saar WE, Lee TH, Berlet GC: The economic burden of diabetic foot and ankle disorders. Foot Ankle Int 26:27-31, 2005.
51. Cruz CP, Eidt JF, Capps C, Kirtley L, Moursi MM: Major lower extremity amputations at a Veterans Affairs hospital. Am J Surg 186:449-454, 2003.
52. Andros G: Diagnostic and therapeutic arterial interventions in the ulcerated diabetic foot. Diabetes Metab Res Rev 20 Suppl 1:S29-S33, 2004.
53. Margolis DJ, Len-Taylor L, Hoffstad O, Berlin JA: Healing diabetic neuropathic foot ulcers: are we getting better? Diabet Med 22:172-176, 2005.
54. Chantelau E, Tanudjaja T, Altenhofer F, Ersanli Z, Lacigova S, Metzger C: Antibiotic treatment for uncomplicated neuropathic forefoot ulcers in diabetes: a controlled trial. Diabet Med 13:156-159, 1996.
55. Lipsky BA, Itani K, Norden C: Treating foot infections in diabetic patients: a randomized, multicenter, open-label trial of linezolid versus ampicillin-sulbactam/amoxicillin-clavulanate. Clin Infect Dis 38:17-24, 2004.
56. Lew DP, Waldvogel FA: Osteomyelitis. N Engl J Med 336:999-1007, 1997.
57. Senneville E, Yazdanpanah Y, Cazaubiel M, Cordonnier M, Valette M, Beltrand E, Khazarjian A, Maulin L, Alfandari S, Caillaux M, Dubreuil L, Mouton Y: Rifampicin-ofloxacin oral regimen for the treatment of mild to moderate diabetic foot osteomyelitis. J Antimicrob Chemother 48:927-930, 2001.
58. Venkatesan P, Lawn S, Macfarlane RM, Fletcher EM, Finch RG, Jeffcoate WJ: Conservative management of osteomyelitis in the feet of diabetic patients. Diabet Med 14:487-490, 1997.
59. Boulton AJ, Armstrong DG: Whither progress in the diabetic foot--clinical and research? Int J Low Extrem Wounds 3:182-183, 2004.
60. Lipsky BA, Berendt AR, Deery HG, Embil JM, Joseph WS, Karchmer AW, Lefrock JL, Lew DP, Mader JT, Norden C, Tan JS: Diagnosis and treatment of diabetic foot infections. Clin Infect Dis 39:885-910, 2004.
61. Macfarlane RM, Jeffcoate WJ: Factors contributing to the presentation of diabetic foot ulcers. Diabet Med 14:867-870, 1997.
62. Leymarie F, Richard JL, Malgrange D: Factors associated with diabetic patients at high risk for foot ulceration. Diabetes Metab 31:603-605, 2005.

63. Boulton AJ, Kirsner RS, Vileikyte L: Clinical practice. Neuropathic diabetic foot ulcers. N Engl J Med 351:48-55, 2004.
64. Chantelau E, Kushner T, Spraul M: How effective is cushioned therapeutic footwear in protecting diabetic feet? A clinical study. Diabet Med 7:355-359, 1990.
65. Uccioli L, Faglia E, Monticone G, Favales F, Durola L, Aldeghi A, Quarantiello A, Calia P, Menzinger G: Manufactured shoes in the prevention of diabetic foot ulcers. Diabetes Care 18:1376-1378, 1995.
66. Valk GD, Kriegsman DM, Assendelft WJ: Patient education for preventing diabetic foot ulceration. A systematic review. Endocrinol Metab Clin North Am 31:633-658, 2002.
67. Calle-Pascual AL, Duran A, Benedi A, Calvo Mi, Charro A, Diaz JA, Calle JR, Gil E, Maranes JP, Cabezas-Cerrato J: A preventative foot care programme for people with diabetes with different stages of neuropathy. Diabetes Res Clin Pract 57:111-117, 2002.
68. Dalla PL, Faglia E, Caminiti M, Clerici G, Ninkovic S, Deanesi V: Ulcer recurrence following first ray amputation in diabetic patients: a cohort prospective study. Diabetes Care 26:1874-1878, 2003.
69. Thompson P, Langemo D, Hunter S, Hanson D, Anderson J: Offloading diabetic foot ulcers. Adv Skin Wound Care 19:15-6, 19, 2006.
70. Van SC, Ulbrecht JS, Becker MB, Cavanagh PR: Design criteria for rigid rocker shoes. Foot Ankle Int 21:833-844, 2000.
71. Edmonds ME, Blundell MP, Morris ME, Thomas EM, Cotton LT, Watkins PJ: Improved survival of the diabetic foot: the role of a specialized foot clinic. Q J Med 60:763-771, 1986.
72. Larsson J, Apelqvist J, Agardh CD, Stenstrom A: Decreasing incidence of major amputation in diabetic patients: a consequence of a multidisciplinary foot care team approach? Diabet Med 12:770-776, 1995.
73. Frykberg RG: The team approach in diabetic foot management. Adv Wound Care 11:71-77, 1998.
74. Jeffcoate WJ, Lipsky BA, Berendt AR, Cavanagh PR, Bus SA, Peters EJ, et Al. Unresolved issues in the management of ulcers of the foot in diabetes. Diabet Med. 2008 Dec;25(12):1380-9.
75. Sinacore DR. Healing times of pedal ulcers in diabetic immunosuppressed patients after transplantation. Arch Phys Med Rehabil. 1999 Aug;80(8):935-40.
76. Ghanassia E, Villon L, Thuan Dit Dieudonne JF, Boegner C, Avignon A, Sultan A. Long-term outcome and disability of diabetic patients hospitalized for diabetic foot ulcers: a 6.5-year follow-up study. Diabetes Care. 2008 Jul;31(7):1288-92.
77. Preventive foot care in people with diabetes. Diabetes Care. 2000 Jan;23 Suppl 1:S55-6.
78. Boulton AJ, Jude EB. Therapeutic footwear in diabetes: the good, the bad, and the ugly? Diabetes Care. 2004;27(7):1832-3.

79. Arts ML, Waaijman R, de Haart M, Keukenkamp R, Nollet F, Bus SA. Offloading effect of therapeutic footwear in patients with diabetic neuropathy at high risk for plantar foot ulceration. Diabet Med. 2012;29(12):1534-41.
80. Fernandez ML, Lozano RM, Diaz MI, Jurado MA, Hernandez DM, Montesinos JV. How effective is orthotic treatment in patients with recurrent diabetic foot ulcers? J Am Podiatr Med Assoc. 2013;103(4):281-90.
81. Bus SA. Priorities in offloading the diabetic foot. Diabetes Metab Res Rev. 2012;28(Suppl 1):54-9.
82. Bus SA, Ulbrechtb JS, Cavanagh PR. Pressure relief and load redistribution by custom-made insoles in diabetic patients with neuropathy and foot deformity . Clinical biomechanics 2004.volum 19, issue 6, pg 629-638.
83. Wu WL, Rosenbaum D, Su FC. The effects of rocker sole and SACH heel on kinematics in gait. Med Eng Phys. 2004;26(8):639-46.
84. Myers KA1, Long JT, Klein JP, Wertsch JJ, Janisse D, Harris GF. Biomechanical implications of the negative heel rocker sole shoe: gait kinematics and kinetics. Gait Posture. 2006;24(3):323-30.
85. Brown D, Wertsch JJ, Harris GF, Klein J, Janisse D. Effect of rocker soles on plantar pressures. Arch Phys Med Rehabil. 2004;85(1):81-6.
86. Praet SF, Louwerens JW. The influence of shoe design on plantar pressures in neuropathic feet. Diabetes Care. 2003;26(2):441-5.
87. Healy A, Naemi R, Chockalingam N. The effectiveness of footwear as an intervention to prevent or to reduce biomechanical risk factors associated with diabetic foot ulceration: a systematic review. J Diabetes Complications. 2013;27(4):391-400.
88. Mayfield JA, Sugarman JR. The use of the Semmes-Weinstein monofilament and other threshold tests for preventing foot ulceration and amputation in persons with diabetes. J Fam Pract. 2000 Nov; 49(11uppl):S17-29.
89. Reiber GE. The epidemiology of diabetic foot problems. Diabet Med. 1996;13(suppl 1):S6-S11.
90. Prompers L, Huijberts M, Schaper N, Apelqvist J, Bakker K, Edmonds M, et al. Resource utilisation and costs associated with the treatment of diabetic foot ulcers. Prospective data from the Eurodiale Study. Diabetologia. 2008;51(10):1826-34.
91. Yong R, Karas TJ, Smith KD, Petrov O. The durability of the Semmes-Weinstein 5.07 monofilament. J Foot Ankle Surg. 2000 Jan-Feb; 39(1):34-8.
92. Armstrong DG, Lavery LA, Vela SA, Quebedeaux TL, Fleischli JG. Choosing a practical screening instrument to identify patients at risk for diabetic foot ulceration. Arch Intern Med. 1998 Feb 9; 158(3):289-92.

93. Kalani M, Brismar K, Fargrell B, Östergren J, Jörneskog G. "Transcutaneous oxygen tensión and toe blood pressure as predictors for outcome of diabetic foot ulcers". Diabetes Care 22:147-151, 1999.
94. Michaud TC: "Abnormal motion during the gait cycle", in Foot Orthoses and other conservative foot care. Ed by Thomas C. Michaud, p 57-173, Williams & Wilkins. Newton, Massachusetts, 1991.
95. Root ML, Orien WP, Weed JH, Hugues RJ. Exploración Biomecánica del Pie. VOL 1. Ortocen SA. Madrid. 1991.
96. Molines-Barroso RJ, Lázaro-Martínez JL, Aragón-Sánchez FJ, Álvaro-Afonso FJ, García-Morales E, García-Álvarez Y. Forefoot ulcer risk is associated with foot type in patients with diabetes and neuropathy. Diabetes Res Clin Pract. 2016 Jan 14. pii: S0168-8227(16)00057-7. doi: 10.1016/j.diabres.2016.01.008.
97. Bacarin TA, Sacco IC, Hennig EM. Plantar pressure distribution patterns during gait in diabetic neuropathy patients with a history of foot ulcers. Clinics (Sao Paulo). 2009;64(2):113-20.
98. Craig CL, Marshall AL, Sjöström M, Bauman AE, Stand ML, Ainsworth BE, et al. International physical activity ques- tionnaire: 12-country reliability and validity. Med Sci Sports Exerc 2003;35:1381e95.
99. Bakker K, Apelqvist J, Schaper NC. Practical guidelines on the management and prevention of the diabetic foot 2011. Diabetes Metab Res Rev 2012; 28(Suppl 1): 225–231.

ANEXOS

ANEXO 1:

CALZADO TERAPEÚTICO

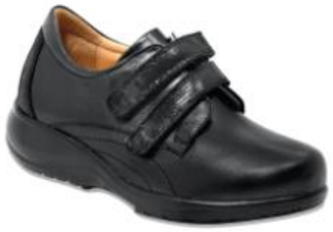

CORTINA GRAN VOLUME

- ✓ Talla: 35-42
- ✓ Volumen extra
- ✓ Sin costuras internas
- ✓ Parte superior autodeformable
- ✓ **Suela semirrígida / rígida**
- ✓ Aspecto clásico

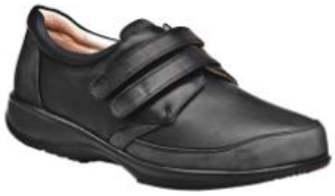

X-DIAB-14

- ✓ Talla: 39-46
- ✓ Volumen extra
- ✓ Sin costuras internas
- ✓ Parte superior autodeformable
- ✓ **Suela semirrígida / rígida**
- ✓ Aspecto clásico

ANEXO 2:

HOJA DE INFORMACIÓN AL PACIENTE Y CONSENTIMIENTO INFORMADO DEL ESTUDIO: "Eficacia clínica del calzado terapéutico con balancín para la prevención de la re-ulceración en los pacientes con Diabetes mellitus y polineuropatía diabética: ensayo clínico prospectivo y aleatorizado".

La Diabetes *mellitus* es una enfermedad metabólica que consiste en el aumento de los niveles de "azúcar" (glucosa) en sangre. Con el paso de los años y debido a un mal control de los niveles de glucosa en sangre, la Diabetes puede producir complicaciones en diferentes zonas del organismo, afectando a órganos como el riñón, el ojo o el corazón. Además de estas complicaciones se pueden producir a nivel de los miembros inferiores, y sobre todo en los pies, degeneraciones en nervios y arterias que disminuyen la sensibilidad y dificultan la circulación en las zonas alejadas del corazón.

Cuando aparecen alteraciones de la sensibilidad en los pies o se disminuye el riego sanguíneo de éstos, se corre el riesgo de sufrir el síndrome denominado "pie diabético", el cuál se caracteriza por la presencia de úlceras o heridas en los pies que normalmente cicatrizan con dificultad, suponiendo una complicación que dependiendo de la gravedad de las misma puede llevar a la amputación.

Con el objeto de prevenir y reducir el número de amputaciones que sufren los pacientes diabéticos es necesario estudiar en profundidad los factores de riesgo que conducen al padecimiento del pie diabético. Es por tanto fundamental definir qué tipos de pies, qué deformidades y qué tipo de calzado se asocian con un mayor riesgo de sufrir una úlcera, para identificar de forma precoz a estos pacientes y someterlos a un tratamiento preventivo y a un seguimiento estrecho que evite el desarrollo de estas heridas en los pies.

Para poder desarrollar este estudio necesitamos pedirle su consentimiento para realizar un análisis biomecánico pormenorizado de los miembros inferiores el cuál consiste en la observación (inspección clínica) de su forma del pie y de las deformidades que pudiera presentar. Así mismo necesitamos analizar el rango de movimiento articular de las principales articulaciones del pie y la posición de diferentes estructuras del pie con respecto a otras. Estas exploraciones clínicas son totalmente indoloras, se realizan con el paciente tumbado en una camilla y en ningún momento se realizan maniobras dolorosas o incómodas para el paciente. Tampoco se utilizan materiales o instrumentos que pudieran herir la piel o provocar heridas en los pies de los pacientes. Todas las maniobras clínicas a

realizar forman parte del protocolo de exploración biomecánica que se realiza a cualquier paciente que acude a la Clínica Universitaria de Podología de la Universidad Complutense de Madrid.

También le someteremos a un análisis computerizado de las presiones plantares del pie. Se trata de caminar sobre una plataforma podobarométrica (que mide la presión) que está conectada a un ordenador, de tal forma que cuando el paciente pisa descalzo sobre ella, el ordenador capta las presiones (el reparto del peso) que recibe el pie durante la marcha.

De igual forma a todos los pacientes incluidos en el estudio se les realiza una valoración del estado neurológico y vascular a nivel de los miembros inferiores. Estas pruebas consisten en la colocación de unos instrumentos calibrados en el pie, para que el paciente informe acerca de su sensibilidad (si los siente o no). Estas pruebas son inocuas y están recomendadas a todos los pacientes diabéticos como parte del seguimiento de su enfermedad, de cara a detectar pérdidas de sensibilidad en los pies que pudieran exponerlo a sufrir heridas.

La exploración vascular consiste en palpar los pulsos del pie (pedio y tibial posterior) y realizar la toma de la Tensión Arterial en el pie mediante la utilización de un doppler. Ambas pruebas son también inocuas para el paciente.

Por último se le prescribirá un calzado terapéutico y dependiendo del grupo de estudio en el que entre de manera aleatoria, dicho calzado podrá ser modificado externamente evaluándose la adaptación al calzado, así como las complicaciones asociadas.

La utilización de los datos que estos estudios aporten tiene como fin exclusivo la investigación de los factores de riesgo de padecer úlcera en los pies de los diabéticos y su tasa de recurrencia. Los resultados se comunicarán en los medios habituales de difusión científica, incluyendo presentaciones orales o en formato póster en Congresos, Jornadas u otras reuniones de interés científico; publicaciones técnicas y otros medios de divulgación profesional, salvaguardando siempre la intimidad del paciente y sus procesos patológicos conocidos. (Ley Orgánica de Protección de Datos (LOPD) 15/1999, de 13 de diciembre).

Así mismo autorizo a los profesionales encargados de este estudio a la realización de fotografías de mis pies y piernas, siempre excluyendo en la imagen mi rostro o cualquier otro matiz que pudiera quebrantar el derecho a mi intimidad y al secreto profesional, y con

el fin único de su uso docente o investigador, no pudiendo las mismas ser vendidas o cedidas a ninguna otra persona o institución sin mi conocimiento y autorización expresa.

Los datos de los pacientes recogidos en el presente estudio serán manejados por un equipo de investigadores cuyo investigador principal es el Prof. Dr. José Luis Lázaro Martínez (Subdirector de la Clínica Universitaria de Podología y Jefe del Servicio de Pie Diabético) de la Universidad Complutense de Madrid. Estos datos están sometidos a la legislación reguladora por Ley Orgánica de Protección de Datos (LOPD) 15/1999, de 13 de diciembre y a ley 41/2002, de 14 de noviembre, básica reguladora de la autonomía del paciente y de derechos y obligaciones en materia de información y documentación clínica.

Así mismo, la legislación vigente en materia de protección de datos (Ley Orgánica 15/1999), reconoce una serie de derechos a los ciudadanos, como son el derecho de acceso, rectificación, cancelación y oposición de sus datos personales. El ejercicio de los mismos es personalísimo, y debe, por tanto, ser ejercido directamente por los interesados ante el responsable/titular de los ficheros, lo que significa que usted puede dirigirse a la Clínica Universitaria de Podología de la Universidad Complutense de Madrid, solicitando información sobre qué datos tienen y cómo los han obtenido (derecho de acceso), la rectificación de los mismos (derecho de rectificación), o en su caso, la cancelación de los datos en sus ficheros (derecho de cancelación). Por último, según el derecho de oposición, los titulares de los datos pueden instar la oposición al tratamiento automatizado de ese tipo de datos, de conformidad con lo previsto en el artículo 6.4 de la Ley Orgánica 15/1999, de 13 de diciembre, que establece: "...En los casos en los que no sea necesario el consentimiento del afectado para el tratamiento de los datos de carácter personal, y siempre que una Ley no disponga lo contrario, éste podrá oponerse a su tratamiento cuando existan motivos fundados y legítimos relativos a una concreta situación personal. En tal supuesto, el responsable del fichero excluirá del tratamiento los datos relativos al afectado."

En todos los casos, deberá dirigirse directamente al responsable del fichero (investigador principal: Prof. Dr. José Luis Lázaro Martínez) en donde se encuentren sus datos personales, utilizando cualquier medio que permita acreditar el envío y la recogida de su solicitud para el ejercicio de sus derechos, acompañando copia de su D.N.I. e indicando el fichero o ficheros a consultar. Si en el plazo de un mes desde la recepción de la solicitud de derecho de acceso en la oficina referida, no ha sido atendida adecuadamente, podrá dirigirse a la Agencia Española de Protección de Datos con copia de la solicitud cursada y de la contestación recibida (si existiera), para que ésta a su vez se dirija a la oficina designada con el objetivo de hacer efectivo el ejercicio de ese derecho. En el caso

expuesto, podrá acceder a la información pretendida si se trata de información sobre sus datos personales, pero no si se trata de información de terceros. Efectivamente, el derecho de acceso no puede ser ejercitado en intervalos inferiores a 12 meses, salvo que se acredite un interés legítimo.

D……………………………………………………….con DNI………………consiento voluntariamente participar en el presente estudio.

D………………………………………………………. con DNI…………………..No Consiento participar en el presente estudio.

En Madrid, a .... de .... de 20..

Fdo………………………….. Fdo. Dr. José Luis Lázaro Martínez

El paciente Investigador Principal

ANEXO 3:

CUESTIONARIO INTERNACIONAL DE ACTIVIDAD FISICA

IPAQ: FORMATO CORTO AUTOADMINISTRADO DE LOS ULTIMOS 7 DIAS

1. PARA SER UTILIZADO CON ADULTOS JOVENES Y DE MEDIANA EDAD(15- 69 años)

Los cuestionarios internacionales sobre actividad física (IPAQ) comprenden una serie de 4 cuestionarios. Las versiones disponibles son: largos (5 campos de actividad sobre los que se pregunta individualmente) y cortos (4 ítems genéricos), para ser utilizados por vía telefónica o autoadministrados. La finalidad de estos cuestionarios es proporcionar instrumentos comunes que puedan usarse para obtener información internacional comparable sobre la actividad física relacionada con la salud.

Antecedentes de IPAQ

El desarrollo de un sistema de medición internacional de la actividad física comenzó en Ginebra en 1998, y continuó con ensayos extensivos de confiabilidad y validación llevados a cabo en 12 países (14 lugares), en 6 continentes durante el 2000. Los resultados finales sugieren que estas mediciones tienen atributos aceptables de medición para aplicar en muchos escenarios y en diferentes idiomas, y son adecuados para los estudios de prevalencia basados en poblaciones nacionales sobre la participación en la actividad física.

El uso de IPAQ

Se alienta el uso de los instrumentos de IPAQ a nivel mundial para fines de monitoreo e investigación. Se recomienda no cambiar el orden o lenguaje de las preguntas, ya que esto afectaría las propiedades psicométricas de los instrumentos.

Traducción del Inglés y Adaptación Cultural

Se apoya la traducción del inglés para facilitar el uso mundial de IPAQ. La información sobre la disponibilidad de IPAQ en diferentes lenguas puede obtenerse en **www.ipaq.ki.se** Si se emprende una nueva traducción, recomendamos fuertemente el uso de los métodos de retro traducción disponibles en el sitio Web. De ser posible, por favor piense en hacer que su versión traducida de IPAQ esté disponible para otros como contribución al sitio Web de IPAQ. Detalles adicionales sobre la traducción y la adaptación cultural pueden descargarse desde el sitio Web.

Desarrollos Adicionales de IPAQ

La colaboración internacional con IPAQ está en marcha, y un ***Estudio Internacional de Prevalencia de Actividad Física*** está en progreso. Para información adicional vea el sitio Web.

Más Información

Información más detallada sobre el proceso de IPAQ y los métodos de investigación utilizados para desarrollar los instrumentos de IPAQ se encuentran disponibles en **www.ipaq.ki.se** y en Booth, M.L (2000). *Assessment of Physical Activity: An International Perspective. Research Quarterly for Exercise and Sport, 71(2):s114-20*. Otras

presentaciones y publicaciones científicas sobre el uso de IPAQ se resumen en el sitio Web.

www.ipaq.ki.se

CUESTIONARIO INTERNACIONAL DE ACTIVIDAD FISICA

Estamos interesados en averiguar acerca de los tipos de actividad física que hace la gente en su vida cotidiana. Las preguntas se referirán al tiempo que usted destinó a estar físicamente activo en los **últimos 7 días**. Por favor responda a cada pregunta aún si no se considera una persona activa. Por favor, piense acerca de las actividades que realiza en su trabajo, como parte de sus tareas en el hogar o en el jardín, moviéndose de un lugar a otro, o en su tiempo libre para la recreación, el ejercicio o el deporte.

Piense en todas las actividades **intensas** que usted realizó en los **últimos 7 días.** Las actividades físicas **intensas** se refieren a aquellas que implican un esfuerzo físico intenso y que lo hacen respirar mucho más intensamente que lo normal. Piense *solo* en aquellas actividades físicas que realizó durante por lo menos **10 minutos** seguidos.

1. Durante los **últimos 7 días**, ¿en cuantos realizó actividades físicas **intensas** tales como levantar pesos pesados, cavar, hacer ejercicios aeróbicos o andar rápido en bicicleta?

______ **días por semana**

☐ Ninguna actividad física intensa → ***Vaya a la pregunta 3***

2. Habitualmente, ¿cuánto tiempo en total dedicó a una actividad física **intensa** en uno de esos días?

______ **horas por día**

______ **minutos por día**

☐ No sabe/No está seguro

Piense en todas las actividades **moderadas** que usted realizó en los **últimos 7 días**. Las actividades **moderadas** son aquellas que requieren un esfuerzo físico moderado que lo hace respirar algo más intensamente que lo normal. Piense *solo* en aquellas actividades físicas que realizó durante por lo menos **10 minutos** seguidos.

3. Durante los **últimos 7 días**, ¿en cuántos días hizo actividades físicas **moderadas** como transportar pesos livianos, andar en bicicleta a velocidad regular o jugar dobles de tenis? **No** incluya caminar.

______ **días por semana**

☐ Ninguna actividad física moderada → ***Vaya a la pregunta 5***

4. Habitualmente, ¿cuánto tiempo en total dedicó a una actividad física **moderada** en uno de esos días?

______ **horas por día**

______ **minutos por día**

☐ No sabe/No está seguro

Piense en el tiempo que usted dedicó a **caminar** en los **últimos 7 días**. Esto incluye caminar en el trabajo o en la casa, para trasladarse de un lugar a otro, o cualquier otra caminata que usted podría hacer solamente para la recreación, el deporte, el ejercicio o el ocio.

5. Durante los **últimos 7 días**, ¿En cuántos **caminó** por lo menos **10 minutos** seguidos?

______ **días por semana**

☐ Ninguna caminata ➡ ***Vaya a la pregunta 7***

6. Habitualmente, ¿cuánto tiempo en total dedicó a caminar en uno de esos días?

______ **horas por día**

______ **minutos por día**

☐ No sabe/No está seguro

La última pregunta es acerca del tiempo que pasó usted **sentado** durante los días hábiles de los **últimos 7 días**. Esto incluye el tiempo dedicado al trabajo, en la casa, en una clase, y durante el tiempo libre. Puede incluir el tiempo que pasó sentado ante un escritorio, visitando amigos, leyendo, viajando en ómnibus, o sentado o recostado mirando la televisión.

7. Durante los **últimos 7 días** ¿cuánto tiempo pasó **sentado** durante un **día hábil**?

______ **horas por día**

______ **minutos por día**

☐ No sabe/No está seguro

ANEXO 4:

REGISTRO DE VARIABLES GENERALES

**SEXO:**	**FECHA NACIMIENTO:**	
**TIPO DM:**<br>□1<br>□2	**EVOLUCIÓN DM (AÑOS):**<br>**HbA1c:** ______________________ %	
TALLA<br>______________________ cm	Peso ______________________________________ Kg	
**IMC:**		
□ **RETINOPATÍA**		
□ **ENF. RENAL**	Estadio:	
	**PIE DERECHO**	**PIE IZQUIERDO**
□ **ANTECEDENTES ÚLCERA**	Localización:	Localización:
**ÚLCERA PLANTAR**	O Si<br>O No<br>O Dedo_____________<br>O Metatarsiano_____	O Si<br>O No<br>O Dedo_____________<br>O Metatarsiano_____
□ **ANTECEDENTES AMPUTACIÓN**	Localización:	Localización:

**EXPLORACIÓN NEUROLÓGICA**	**AFECTACIÓN**		**NO AFECTACIÓN**	
□ MONOFILAMENTO	PD	PI	PD	PI
□ BIOTENSÓMETRO	PD	PI	PD	PI
**EXPLORACIÓN VASCULAR**	**PIE DERECHO**		**PIE IZQUIERDO**	
ITB				
IDB				
PULSO PEDIO	PRESENTE	AUSENTE	PRESENTE	AUSENTE
PULSO TIBIAL POSTERIOR	PRESENTE	AUSENTE	PRESENTE	AUSENTE
**DEFORMIDADES**	**PIE DERECHO**		**PIE IZQUIERDO**	
□Dedo en garra				
□Hallux abductus valgus				
□Taylor bunion				

GONIOMETRIA ARTICULAR

VARIABLE (grados)	GONIOMETRÍA ARTICULAR	
	PIE DERECHO	PIE IZQUIERDO
FD TPA rodilla extensión		
FD TPA rodilla en carga		
FD 1ªMTF en carga		
FD 1ªMTF en descarga		
Inversión ASA		
Eversión ASA		

INDICE POSTURAL DEL PIE

6 CRITERIOS	Índice postural del pie		
		**PD**	PI
**RETROPIE**	**Palpación de la cabeza del astrágalo**		
	**Curvatura supra e infra-maleolar lateral**		
	**Posición del calcáneo en el plano frontal**		
**ANTPIE**	**Prominencia de la región talo-navicular**		
	**Congruencia del arco longitudinal interno**		
	**Abducción / aducción del antepié respecto al retropié**		
**TOTAL**			

a) Pie neutro: 0 a + 5 / b) Pie pronado: + 6 a + 9 / c) Pie altamente pronado: + 10 a + 12. / d) Pie supinado:- 1 a - 4. / e) Pie altamente supinado:- 5 a -12

ANEXO 5:

TABLA DE VARIABLES.

Nombre	Descripción	Tipo	Valores
NUMERO REGISTRO	Número con que se identifica al paciente en el registro del estudio	Cuantitativa	
NUMERO HISTORIA	Número con que se identifica al paciente en el centro de referencia al que acude	Cuantitativa	
NOMBRE	Nombre del paciente	Cualitativa	
APELLIDOS	Apellidos del paciente	Cualitativa	
FECHA NACIMIENTO	Dia/mes/año	Cuantitativa	
EDAD	Tiempo que una persona ha vivido, a contar desde que nació	Cuantitativa	
SEXO	Género humano/ Conjunto de seres pertenecientes a un mismo sexo	Cualitativa	- HOMBRE<br>- MUJER
TIPO DIABETES MELLITUS	Identificación del tipo de DM que tiene el paciente (Tipo 1 o 2 y si es o no insulinodependiente)	Cualitativa	- 1<br>- 2
AÑOS EVOLUCION DIABETES MELLITUS	Tiempo transcurrido desde inicio enfermedad	Cuantitativa	
HEMOGLOBINA GLICOSILADA	Valor medio de los niveles de glucemia en los últimos meses	Cuantitativa	
PESO	Peso en kilogramos (Kg.) del paciente	Cuantitativa	
TALLA	Altura en metros (m) del paciente	Cuantitativa	
ÍNDICE DE MASA CORPORAL	Es un número que pretende determinar, a partir de la estatura y la masa de una persona, si su masa se encuentra en un intervalo saludable. Se utiliza como indicador nutricional y es el método más práctico para evaluar el grado de riesgo asociado con la obesidad. El índice de masa corporal	Cuantitativa	

	resulta de la división de la masa en kilogramos entre el cuadrado de la estatura expresada en metros. IMC= masa (Kg.)/talla $(m)^2$		
ANT. RETINOPATIA	La retinopatía diabética es una complicación ocular de la diabetes, causada por el deterioro de los vasos sanguíneos que irrigan la retina del fondo de ojo. Estos vasos sanguíneos debilitados pueden dejar salir líquido o sangre, formar ramas frágiles en forma de cepillo, y agrandarse en ciertos lugares.	Cualitativa	- NO<br>- SÍ
ANT. NEFROPATIA	Es una complicación de la diabetes y, si una persona la padece, su riñón pierde la capacidad para funcionar apropiadamente. Esta afección se caracteriza por niveles altos de proteína en la orina.	Cualitativa	- NO<br>- SÍ
ANT. ULCERA	Presencia de ulceración previa	Cualitativa	- NO<br>- SÍ
ANT. AMPUTACION	Presencia de amputación previa	Cualitativa	- NO<br>- SÍ
TIPO DE PIE	Determinación del tipo de pie según la afectación vascular o neuropática que presente	Cualitativa	- NEUROPÁTICO<br>- NEUROVASCULAR
MONOFILAMENTO	Prueba de screening neurológico	Cualitativa	- CON AFECTACIÓN<br>- SIN AFECTACIÓN
BIOTENSIÓMETRO	Prueba de screening neurológico	Cualitativa	- CON AFECTACIÓN<br>- SIN AFECTACIÓN
ÍNDICE TOBILLO/BRAZO	Prueba de screening vascular	Cualitativa	- NORMAL 0.9 - 1,1<br>- CALCIFICACIÓN ARTERIAL > 1,1<br>- ISQUEMIA MODERADA 0,5 - 0,9<br>- ISQUEMIA CRÍTICA <0.5

ÍNDICE DEDO/BRAZO	Prueba de screening vascular	Cualitativa	- NORMAL 0.64 - 1,0<br>- CALCIFICACIÓN ARTERIAL > 1,0<br>- ISQUEMIA <0.64
CALCIFICACIÓN ARTERIAL	Se caracteriza por la formación de placas calcáreas, a veces osificadas, en la túnica media especialmente en las arterias de las extremidades. Las placas se disponen a manera de anillos como en la tráquea y pueden palparse al tomar el pulso en la arteria radial	Cualitativa	- NO<br>- SÍ
PULSO PEDIO	Palpación de arteria pedia	Cualitativa	- PALPABLE<br>- NO PALPABLE
PULSO TIBIAL POSTERIOR	Palpación de arteria tibial posterior	Cualitativa	- PALPABLE<br>- NO PALPABLE
PATOLOGÍA DIGITAL GARRA	Deformidad digital caracterizada por la flexión dorsal de la falange proximal, flexión plantar de la falange media y flexión plantar de la falange distal (en cm.)	Cualitativa	- NO<br>- SÍ
PATOLOGÍA DIGITAL MARTILLO	Deformidad digital caracterizada por la flexión dorsal de la falange proximal, flexión plantar de la falange media y flexión dorsal de la falange distal (en cm.)	Cualitativa	- NO<br>- SÍ
PATOLOGÍA DIGITAL HAV	Deformidad digital caracterizada por la desviación en abducción y valgo del primer dedo	Cualitativa	- NO<br>- SÍ
PATOLOGÍA DIGITAL T.BUNION	Es una protuberancia en el quinto hueso metatarsiano, en la base del dedo pequeño del pie	Cualitativa	- NO<br>- SÍ
ÍNDICE DE POSTURA DEL PIE	Determinar patología del pie según su forma	Cualitativa	- ÁLTAMENTE PRONADO<br>- PRONADO<br>- NEUTRO<br>- SUPINADO<br>- ÁLTAMENTE SUPINADO

ARTICULACIÓN TIBIOPERONEA ASTRAGALINA	La valoración articular se realiza con el paciente en decúbito prono sobre la camilla de exploración. La valoración es la **flexión dorsal de la ATPA** con la rodilla en flexión y en extensión. Para ello trazamos la línea media del tercio distal y lateral de la pierna a explorar y otra línea en la zona plantar y lateral del calcáneo y manteniendo la articulación subastragalina neutra, flexionamos dorsalmente el pie y registramos los grados de dorsiflexión de la ATPA.	Cuantitativa	
PRIMERA ARTICULACIÓN METATARSO-FALÁNGICA	La valoración articular se realiza con el paciente en sedestación y en bipedestación. La valoración es la **flexión dorsal de la 1ª AMTF** en carga y en descarga. Para ello trazamos la línea media de la diáfisis del I metatarsiano, marcamos el punto medio de la cabeza del 1er meta, donde colocaremos el fulcro del goniómetro, y finalmente, trazamos la línea media de la diáfisis de la falange proximal del hallux.	Cuantitativa	
ARTICULACIÓN SUBASTRAGALINA	La valoración de la ASA se realiza trazando con el paciente en decúbito prono. La valoración es la **inversión y eversión de la ASA.** Para ello se traza la bisección de la cara posterior del calcáneo a lo largo de los dos tercios proximales manteniendo la ASA en posición neutra y trazando también la línea media del tercio distal y posterior de la pierna	Cuantitativa	

	evitando el Tendón de Aquiles. Partiendo de la posición neutra de la ASA, se coloca el pie en máxima supinación y con un brazo del goniómetro paralelo al bisector calcáneo y otro brazo paralelo a la bisección de la pierna, se mide el ángulo resultante. Se realiza la misma operación con la ASA en máxima pronación.		
