# Supplementary material for: Clinical efficacy of therapeutic footwear with a rigid rocker sole in the prevention of recurrence in patients with diabetes mellitus and diabetic polineuropathy: A randomized clinical trial
Source: PLoS One. 2019 Jul 11;14(7):e0219537. doi: 10.1371/journal.pone.0219537 (PMC6623964; doi:10.1371/journal.pone.0219537)
Supplement: S5 File — (PDF) [file pone.0219537.s005.pdf]

Hospital Clínico San Carlos

Informe Dictamen Protocolo Favorable
Investigación Clínica con Producto Sanitario

C.I. 16/408-P

13 de octubre de 2016

CEIC Hospital Clínico San Carlos

Dra. Mar García Arenillas
Presidenta del CEIC Hospital Clínico San Carlos

CERTIFICA

1º. Que el CEIC Hospital Clínico San Carlos en su reunión con acta 10.2/16 ha evaluado la propuesta del promotor referida al estudio:

Título: ***"Eficacia clínica del calzado terapéutico con balancín para la prevención de la re-ulceración en los pacientes con Diabetes mellitus y polineuropatía diabética: ensayo clínico prospectivo y aleatorizado".***

Código Interno: 16/408-P
Investigador: Dr. José Luis Lázaro Martínez
Versión Protocolo Evaluada: Versión 2 de octubre de 2016
Versión Hoja Información al Paciente Evaluada: GENERAL / versión de 2 de octubre de 2016

2º. Considera que

- El estudio se plantea siguiendo los requisitos establecidos en la legislación vigente en cuanto a Investigación Clínica con Productos Sanitarios, y su realización es pertinente.
- Se cumplen los requisitos necesarios de idoneidad del protocolo en relación con los objetivos del estudio y están justificados los riesgos y molestias previsibles para el sujeto.
- La capacidad de los Investigadores y los medios disponibles son apropiados para llevar a cabo el estudio.
- Son adecuados tanto el procedimiento para obtener el consentimiento informado como la compensación prevista para los sujetos por daños que pudieran derivarse de su participación en el estudio.
- El alcance de las compensaciones económicas previstas no interfiere con el respeto a los postulados éticos.

3º. Por lo que este CEIC emite un **DICTAMEN FAVORABLE**.

Lo que firmo en Madrid, a 13 de octubre de 2016

Hospital Clínico San Carlos
Comité Etico de Investigación Clínica
Madrid

Fdo.: Dra. Mar García Arenillas
Presidenta del CEIC Hospital Clínico San Carlos

Hospital Clínico San Carlos
Doctor Martín Lagos, s/n. Madrid 28040 Madrid España
Tel. 91 330 34 13 Fax. 91 330 32 99 Correo electrónico ceic.hcsc@salud.madrid.org
